# Adaptive thermogenesis enhances the life-threatening response to heat in mice with an *Ryr1* mutation

**DOI:** 10.1038/s41467-020-18865-z

**Published:** 2020-10-09

**Authors:** Hui J. Wang, Chang Seok Lee, Rachel Sue Zhen Yee, Linda Groom, Inbar Friedman, Lyle Babcock, Dimitra K. Georgiou, Jin Hong, Amy D. Hanna, Joseph Recio, Jong Min Choi, Ting Chang, Nadia H. Agha, Jonathan Romero, Poonam Sarkar, Nicol Voermans, M. Waleed Gaber, Sung Yun Jung, Matthew L. Baker, Robia G. Pautler, Robert T. Dirksen, Sheila Riazi, Susan L. Hamilton

**Affiliations:** 1 Department of Molecular Physiology and Biophysics, Baylor College of Medicine, Houston, TX USA; 2 Translational Biology and Molecular Medicine Graduate Program, Baylor College of Medicine, Houston, TX USA; 3 Department of Pharmacology and Physiology, University of Rochester Medical Center, Rochester, NY USA; 4 Department of Anesthesiology, University of Toronto, Toronto, ON Canada; 5 Advance Technology Core, Mass Spectrometry Proteomics Core, Baylor College of Medicine, Houston, TX USA; 6 Department of Pediatrics, Baylor College of Medicine, Houston, TX USA; 7 Department of Neurology, Donders Institute for Brain, Cognition and Behavior, Radboud University Medical Centre, Nijmegen, Netherlands

**Keywords:** Fat metabolism, Calcium and phosphate metabolic disorders

## Abstract

Mutations in the skeletal muscle Ca^2+^ release channel, the type 1 ryanodine receptor (RYR1), cause malignant hyperthermia susceptibility (MHS) and a life-threatening sensitivity to heat, which is most severe in children. Mice with an MHS-associated mutation in *Ryr1* (Y524S, YS) display lethal muscle contractures in response to heat. Here we show that the heat response in the YS mice is exacerbated by brown fat adaptive thermogenesis. In addition, the YS mice have more brown adipose tissue thermogenic capacity than their littermate controls. Blood lactate levels are elevated in both heat-sensitive MHS patients with *RYR1* mutations and YS mice due to Ca^2+^ driven increases in muscle metabolism. Lactate increases brown adipogenesis in both mouse and human brown preadipocytes. This study suggests that simple lifestyle modifications such as avoiding extreme temperatures and maintaining thermoneutrality could decrease the risk of life-threatening responses to heat and exercise in individuals with *RYR1* pathogenic variants.

## Abstract (web summary)

Individuals with malignant hyperthermia susceptibility (MHS) suffer from lifethreatening responses to heat. Here the authors demonstrate that adaptive thermogenesis from brown adipose tissue contributes to this heat sensitivity in a preclinical mouse model of MHS

## Introduction

Malignant hyperthermia (MH) is a life-threatening response to volatile anesthetics, characterized by muscle rigidity, hyperthermia, rhabdomyolysis, acidosis, tachycardia, and, if untreated, death^1^. The prevalence of MH susceptibility (MHS) has been estimated to be as high as 1 in 2000^2–4^. In adults, MH episodes are more frequently reported in males than females^5,6^. Children are more likely than adults to experience an MH episode in response to anesthesia^7^. Most mutations that underlie human MHS are in the skeletal muscle Ca^2+^ release channel (RYR1)^8,9^. Triggers such as volatile anesthetics and/or heat cause the uncontrolled release of Ca^2+^ from the sarcoplasmic reticulum (SR) via RYR1, leading to sustained muscle contractures. Without proper intervention, the mortality rate following an MH episode in patients is extremely high^8^. Administration of dantrolene, currently the only FDA approved intervention for MH^8^, has effectively reduced the deaths from anesthesia in MH susceptible patients^8^.

Some individuals with MHS variants in *RYR1* suffer from myalgia, rhabdomyolysis and muscle cramps, and can experience a life-threatening MH-like response to heat and/or exercise^10–21^. Exercise, fever^22^ and elevated environmental temperatures with high humidity increase the risk of these MH-like episodes^11^. The heat-induced episodes, however, often occur in nonclinical settings where immediate administration of dantrolene is impractical^23^. Both anesthetic MH episodes and non-anesthetic MH-like responses to heat are highly variable with respect to probability of occurrence and disease presentation both for a single patient and among family members with the same mutation^6,19^. This variability in presentation makes prevention of life-threatening responses to heat or anesthetics challenging.

We previously created mice with a mutation in *Ryr1* (Y524S, YS) homologous to an MHS causative variant in humans (Y522S)^24,25^. The YS mutation in RYR1 decreases the stability of the closed state of the channel, leading to a temperature-dependent Ca^2+^ leak from the SR into the cytosol^24,25^. Increased cytosolic Ca^2+^, in turn, increases the sensitivity of RYR1 to opening in response to membrane depolarization or RYR1 activators (e.g., halothane, caffeine) leading to massive Ca^2+^ release and sustained whole body muscle contractures^25^. Volatile anesthetics, elevated environmental temperatures, and exercise trigger lethal MH and MH-like episodes in YS mice^26^. Similar to human MHS, the MH and MH-like responses of the YS mice are characterized by sustained whole-body muscle contractures which lead to hyperthermia, hyperventilation, rhabdomyolysis and, ultimately, death^24^.

Skeletal muscle^27–30^ and brown adipose tissue (BAT)^31–33^ are the two principal sites for adaptive thermogenesis. BAT is a specialized thermogenic tissue that expresses high levels of the mitochondrial uncoupling protein (UCP1)^31^. In infants and young children, BAT has an established role in maintaining body temperature^34^. Functional imaging has demonstrated that adults also have significant amounts of metabolically active brown fat and the amount of BAT positively correlates with metabolic fitness^35–37^. While adaptive thermogenesis in brown fat is a critical component of temperature and energy regulation in both humans and mice, pathological roles of brown fat are not well-known.

In this study, we investigate the role of adaptive thermogenesis in the response of the YS mice to heat. We demonstrate that conditions that activate BAT thermogenesis increase the probability of an MH-like response during a subsequent heat exposure. Conversely, decreasing BAT activation by small increases in housing temperature of the mice decreases the probability of death of the YS mice upon subsequent exposure to heat. These findings suggest that simple lifestyle modifications could decrease the probability of a life-threatening heat response in humans, especially in children, with *RYR1* mutations. Surprisingly, we found that the thermogenic capacity of YS mice is higher than that of their wild-type (WT) littermates. We demonstrate that this increase in BAT thermogenic capacity is at least partially due to the ability of muscle-derived circulating lactate, elevated in the YS mice, to promote brown/beige adipogenesis by upregulating UCP1 expression.

## Results

### Pediatric and male predominance of heat sensitivity in humans and mice with MHS-associated mutations

**Fig. 1:**
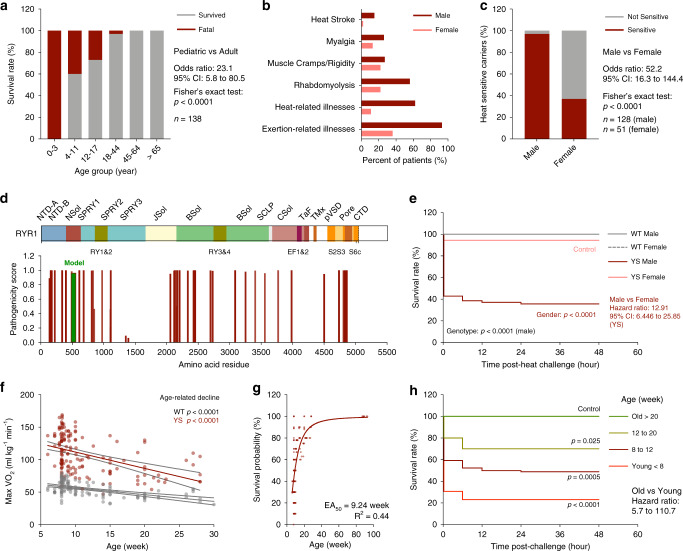
Heat intolerance in patients and mice with *RYR1* pathogenic variants associated with malignant hyperthermia susceptibility (MHS). **a** Survival rate of heat intolerant patients carrying *RYR1* variants associated with MHS among age groups. **b** Symptoms associated with heat intolerance in male and female carriers. **c** Proportion of symptomatic carriers with heat-intolerant symptoms in male and female carriers. **d** Pathogenicity of *RYR1* variants associated with heat-sensitivity. **e** Kaplan–Meier analysis of the survival rate of WT (*n* = 50, male and *n* = 12, female) and YS (*n* = 70, male and *n* = 18, female) mice after acute heat challenge. **f** Relationship of the age of mice and maxVO_2_ during acute heat challenge in WT (*n* = 125) and YS (*n* = 151) mice. **g** Effect of age on the estimated survival probability of YS mice (*n* = 159) after acute heat challenge. **h** Kaplan–Meier analysis of the heat challenge survival rate of YS mice (*n* = 124) grouped by age. All mice were housed at room ambient temperature (20.2 ± 0.4 °C) prior to heat challenge. All mice were within the controlled age range (8.9 ± 0.9 week old) at the time of the study, except for the age-dependent experiments (**f**–**h**). *P* values are indicated as analyzed by Fisher’s exact test (**a**, **c**), Mantel–Cox log-rank test (**e**, **h**), and *F*-test for deviation from zero-slope of linear regression (**f**). All statistical tests are two-sided. *R*^2^ values are indicated to quantify goodness-of-fit to non-linear regression with variable slope (**g**). Data are represented as mean ± 95% confidence intervals (CI) from the linear best-fit line (**f**). Survival probability of each muse is estimated based on the survival rate from ten mice in the respective age subgroups (**g**). Effect of age of the YS mice on survival of the heat challenge was determined by assessing survival probability as a function of age. The EA_50_ is defined as the half-maximal effective age (**g**). The effect of age on survival of individuals (panel **a**) with *RYR1* mutations was also assessed. The pediatric subgroup is defined as 17 years of age and younger, and the adult subgroup is defined as 18 years of age and older. Sample sizes, odds ratios, and 95% confidence intervals (CI) are indicated. Source data are provided as a source data file.

Human *RYR1* mutations that underlie MHS are also frequently associated with heat and/or exercise-induced rhabdomyolysis, exercise intolerance, heat-induced muscle cramps, and death^19,38–40^. We conducted a retrospective cohort study and systematic review on *RYR1* variants, age, gender, clinical symptoms and survival outcomes observed in heat-sensitive individuals with *RYR1* variants associated with MHS. We evaluated the heat sensitivity in human carriers of *RYR1* variants that were referred to the MH Investigation Unit (MHIU) at Toronto General Hospital between 1994 and 2019 (Supplementary Data 1). We also analyzed published case reports and case series of heat and exercise sensitivity associated with *RYR1* mutations (Supplementary Data 2). We found the mortality rate after heat-induced episodes in individuals with *RYR1* variants associated with MHS in the pediatric subgroup was significantly higher compared to that of adults (Fig. 1a). Children (<11 year of age) are the most vulnerable, with a mortality rate of up to 50% following heat-induced episodes. While many children experienced life-threatening responses to heat, adults with similar *RYR1* mutations generally experienced only muscle cramps or exercise intolerance (Supplementary Datas 1 and 2). Similar to previously reported findings for anesthetic-induced MH cases^41,42^, the proportion of heat-sensitive patients observed in male carriers of *RYR1* variants was significantly higher compared to that of the female carriers (Fig. 1b, c). Analysis for the pathogenicity of the heat-sensitivity associated variants in RYR1 using the HumDiv-trained PolyPhen model^43^ confirmed that most of the variants are predicted to be deleterious (Fig. 1d). In particular, highly pathogenic *RYR1* variants associated with heat-sensitivity were frequently found at subunit interfaces near the solenoid structure at the N-terminal domain (Nsol), within the junctional solenoid (JSol), and the bridging solenoid (Bsol) in the cytosolic shell, and at the channel pore domain (Pore) at the C-terminus, based on the previously defined RYR1 domain organization^44^. The heat-sensitivity associated variants mapped to similar domains as the variants associated with pharmacologically induced MH episodes throughout the RYR1 structure with multiple overlapping residues (Supplementary Fig. 1).

We previously reported the heat sensitivity of the mouse model of MH with a *Ryr1*^*Y524S*^ (YS) mutation homologous to a MHS causative mutation^24,28,29^. The YS mice exhibit severe hypermetabolism (elevated maxVO_2_ and maxVCO_2_) and hyperthermia (core body temperature ≥40 °C) during a 15-min exposure to 37 °C (Supplementary Fig. 2), leading to significantly reduced survival within 48 h of the heat exposure^28,29^. Consistent with the male and pediatric predominance in humans, we found that the survival rate for male YS mice was significantly lower than that of female YS mice following the same heat exposure (Fig. 1e). In addition, the heat-induced hypermetabolic response was more severe in young compared to old YS mice (Fig. 1f). While most YS mice less than 8 weeks of age did not survive the heat exposure, we found YS mice over 20 weeks of age were protected from heat-induced death (Fig. 1g, h). Given these findings, we focused our study on defining the underlying mechanisms for the enhanced life-threatening susceptibility to heat within the most vulnerable pediatric male populations. Thus, all experiments discussed here used young (<12 weeks of age) male mice unless otherwise specified. The molecular mechanisms underlying the gender differences in heat sensitivity are currently being explored in a separate study.

MHS has also been associated with a muscular phenotype in human males^41^. While we did not detect significance differences in body weight, lean mass, metabolic rate, energy preference, and activity between YS and WT mice at basal condition, we found a small but significant decrease in overall fat mass in the YS mice, despite increased food consumption (Supplementary Fig. 3).

### Adaptive thermogenesis increases the heat sensitivity of the YS mice by elevating core body temperature

**Fig. 2:**
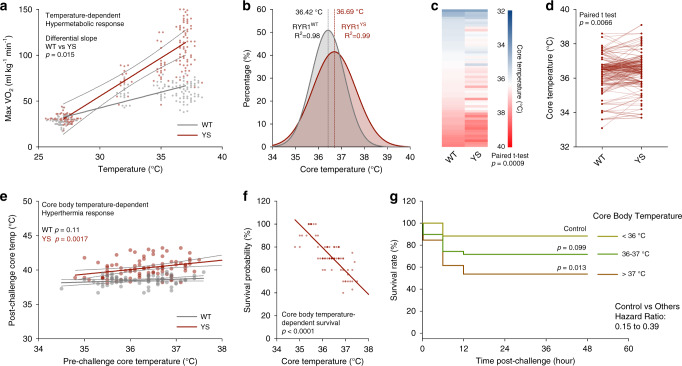
Enhanced heat sensitivity and elevated core body temperature in Y524S mice. **a** Maximum oxygen consumption rate (maxVO_2_) of WT (*n* = 92) and YS (*n* = 115) mice during heat challenge at the indicated temperatures. **b** Distribution of baseline core body temperature of WT (*n* = 175) and YS (*n* = 202) mice at room temperature. **c**, **d** Comparison of baseline core body temperatures of WT and YS mice in littermate pairs analyzed per litter (**c** *n* = 56 pairs) and per individual animals (**d** *n* = 177 pairs). **e** Relationship of baseline core body temperature and hyperthermia response after acute 37 °C heat challenge in WT (*n* = 66) and YS (*n* = 85) mice. **f** Effect of baseline core body temperature on the estimated survival probability of YS mice (*n* = 85) after acute heat challenge. **g** Kaplan–Meier analysis of the heat challenge survival rate of YS mice (*n* = 85) grouped by baseline core body temperature. All mice were housed at room ambient temperature (20.2 ± 0.4 °C) prior to heat challenge. All mice were within the controlled age range (8.9 ± 0.9 week old) at the time of study. *P* values are indicated as analyzed by *F*-test for differential slope of linear regressions (**a**), paired *t* test (**c**, **d**), *F*-test for deviation from zero-slope of linear regression (**e**, **f**), and Mantel–Cox log-rank test (**g**). All statistical tests are two-sided. *R*^2^ values are indicated to quantify goodness-of-fit to Gaussian distribution (**b**). Survival probability of each mouse is estimated based on the survival rate from ten mice in the respective core body temperature (**f**) subgroups. Data are represented as asymmetrical 95% confidence intervals (CI) from linear best-fit line (**a**, **e**). Source data are provided as a source data file.

We have previously demonstrated that the Y524S mutation in RYR1 causes temperature-dependent increases in SR Ca^2+^ leak and muscle basal tension^24,25^. To test the temperature-dependence of the hypermetabolic response in these mice, we exposed YS and WT mice for 15 min to four different temperature conditions and measured maximal VO_2_ using indirect calorimetry (Fig. 2a). The hypermetabolic response is detected at temperatures as low as 32 °C.

Consistent with the previously unexplained observations with multiple mouse model of MHS^45–47^, we detected a small, but significant elevation of baseline core body temperature in the YS mice compared to their WT littermates even in the absence of exposure to heat (Fig. 2b–d). While the core body temperatures of all YS and WT mice were within the physiological range (Fig. 2b), the YS mice had significant elevations in core body temperatures compared to their WT littermates at room temperature prior to heat exposure as assessed with paired *t* tests and analyzed per litter (Fig. 2c) and per individual animal (Fig. 2d). To assess the impact of this small difference in baseline core body temperature on the response to heat, we compared the core body temperature before and after heat exposure (Fig. 2e) and body temperature before heat exposure with survival of the heat challenge (Fig. 2f, g). We found that baseline core body temperature predicts both post-heat exposure body temperature and the survival of the YS mice. These results suggest that small elevations in body temperature prior to heat exposure can sensitize the YS mice to a more severe response to heat and/or heat-induced death. Thus, factors that affect core body temperature such as age, exercise, hormones, stress, and environment, could increase the risk of a life-threatening response to heat in both mice and humans.

**Fig. 3:**
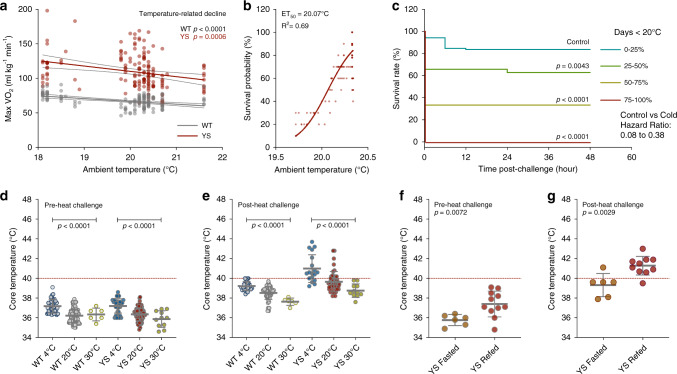
Enhanced heat sensitivity and adaptive thermogenesis in Y524S mice. **a** Relationship of ambient housing temperature and the maxVO_2_ during acute 37 °C heat challenge in WT (*n* = 129) and YS (*n* = 154) mice. **b** Effect of prior median ambient housing temperature on estimated survival probability of YS mice (*n* = 154) after acute heat challenge. **c** Kaplan–Meier analysis of the heat challenge survival rate of YS mice (*n* = 154) grouped by duration of prior ambient housing temperature at lower than 20 °C. **d**, **e** Core body temperature before (**d**) and after (**e**) acute 37 °C heat challenge in WT and YS mice preconditioned for 1 week at 4 °C (WT *n* = 23, YS *n* = 21), room temperature (WT *n* = 35, YS *n* = 40), and 30 °C (WT *n* = 6, YS *n* = 11) ambient environment. **f**, **g** Core body temperature before (**f**) and after (**g**) acute 37 °C heat challenge in YS mice preconditioned at fasted (*n* = 6) and refed state (*n* = 11, post-heat challenge temperature of one refed YS mice was not measured due to full body contracture). All mice were within the controlled age range (8.9 ± 0.9 week old) at the time of study. *P* values are indicated as analyzed by *F*-test for deviation from zero-slope of linear regression (**a**), Mantel–Cox log-rank test (**c**), ordinary one-way analysis of variance (ANOVA) with Dunnett’s multiple comparisons test (**d**, **e**) and Welch’s *t* test (**f**, **g**). All statistical tests are two-sided. *R*^2^ values are indicated to quantify goodness-of-fit to nonlinear regression with variable slope (**b**). Survival probability of each mouse is estimated based on the survival rate from ten mice in the respective ambient temperature (**b**) subgroups. Effect of ambient housing temperature on heat challenge survival is measured by half-maximal effective ambient housing temperature (ET_50_) on the estimated survival probability (**b**). Data are represented as asymmetrical 95% confidence intervals (CI) from linear best-fit line (**a**), or mean ± standard deviation (**d**–**g**). Source data are provided as a source data file.

The correlation of core body temperature prior to heat exposure with survival of the YS mice after heating suggests a possible role for adaptive thermogenesis in sensitizing the YS mice to heat. Adaptive thermogenesis is a bioenergetic process that elevates metabolic rate and generates heat in response to changes in environmental temperature or food intake and is a critical factor for maintenance of the core body temperature in endothermic mammals^48^. Housing temperatures in most animal vivaria are 20–22 °C, which is significantly below thermoneutrality for mice (30–32 °C)^49^. Hence, adaptive thermogenesis at these housing temperatures is required to maintain body temperature in both WT and YS mice. To evaluate the effect of housing temperature on heat response of YS mice, we performed a retrospective study on the relationship between housing temperatures and the response of the YS mice to heat. We found that survival of the heat-exposed YS mice correlated with small fluctuations between 19 and 21 °C in the indoor ambient temperature of the animal housing facility arising from the seasonal changes in air conditioning (Supplementary Fig. 4). The YS mice housed at lower ambient temperatures exhibited more severe responses to the heat exposure (Fig. 3a, b). Notably, while most YS mice did not survive the heat exposure, YS mice housed for longer periods at warmer ambient temperatures, for the most part, were protected from the heat-induced death (Fig. 3c).

To further test the role of adaptive thermogenesis in the sensitivity of the YS mice to heat, we preconditioned mice at controlled cold ambient (4 °C), standard animal facility room temperature (20.2 ± 0.4 °C) or controlled warm ambient (30 °C) for 1 week prior to the heat exposure. Using indirect calorimetry, we detected a marked increase of heat production induced by cold ambient temperatures in both WT and YS mice (Supplementary Fig. 5a–c). This increase in heat production was not associated with changes in cage activity in the YS mice (Supplementary Fig. 5d–f). During the preconditioning in cold, adaptive thermogenesis was adequate to compensate for the decrease in environmental temperature, resulting in no significant change in core body temperature. However, when mice were removed from cold and subjected to warmer room temperature, the adaptive thermogenesis overcompensated to significantly increase core body temperature in both WT and YS mice (Supplementary Fig. 5g–i). The cold-induced adaptive thermogenesis sensitizes the YS mice to heat, leading to severe hyperthermia (≥40 °C) after heat exposure (Fig. 3d, e). In contrast, inhibition of adaptive thermogenesis by preconditioned mice at warm ambient temperature close to thermoneutrality decreased the hyperthermic response in YS mice (Fig. 3d, e).

Food intake also activates adaptive thermogenesis^50^. Food intake following overnight food deprivation (refed) increased both core body temperature (Fig. 3f) and the hypermetabolic response (Fig. 3g) compared to fasted YS mice. Collectively, these results suggest that, while cold and diet-activated thermogenesis is crucial for maintaining core body temperature in WT mice, adaptive thermogenesis can be detrimental to the heat-sensitive YS mice.

BAT, which highly expresses the mitochondrial uncoupling protein (UCP1), is specialized for adaptive thermogenesis. We examined the effects of temperature preconditioning on the morphology of interscapular BAT (iBAT). Consistent with previous reports^27,51^, the iBAT in mice housed at 4 °C displayed multilocular lipid droplets and more compact adipocyte structure (Supplementary Fig. 5j). In contrast, the iBAT in mice exposed to 30 °C had enlarged and unilocular lipid droplets in brown adipocytes, resembling the metabolically dormant white adipocytes (Supplementary Fig. 5j). Consistent with the morphological changes, UCP1 levels were highest in the BAT of both YS and WT mice preconditioned at 4 °C and lowest in these mice preconditioned at 30 °C (Supplementary Fig. 5k, l).

### Genetic and pharmacological modulation of BAT activity alters heat-sensitivity of YS mice

**Fig. 4:**
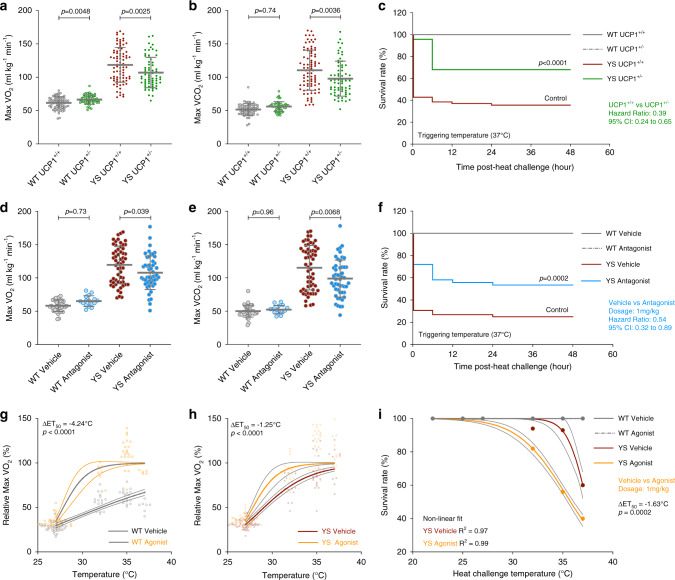
Pharmacological and genetic modulation of adipose tissue thermogenic activity alters heat sensitivity of Y524S mice. **a**, **b** MaxVO_2_ (**a**) and maxVCO_2_ (**b**) of WT (*n* = 108) and YS (*n* = 146) mice with or without heterozygous genetic ablation of the mitochondrial uncoupling protein (Ucp1^WT/null^) during acute heat challenge at 37 °C. **c** Kaplan–Meier analysis of the survival rate of WT (*n* = 108) and YS mice (*n* = 146) with or without *Ucp1*-ablation (heterozygous) after acute heat challenge. **d**, **e** Maximal O_2_ consumption (maxVO_2_, **d**) and CO_2_ production (maxVCO_2_, **e**) rate of WT (*n* = 53) and YS (*n* = 108) mice pretreated with β3-adrenergic receptor (β_3_AR) antagonist (L748337, 1 mg/kg) or vehicle control during acute heat challenge at 37 °C. **f** Kaplan–Meier analysis of the survival rate of WT (*n* = 53) and YS mice (*n* = 108) pretreated with β_3_AR antagonist or vehicle control after acute heat challenge. **g**, **h** Heat sensitivity of WT mice (*n* = 76, **g**) and YS mice (*n* = 106, **h**) pretreated with β_3_AR agonist (BRL37344, 1 mg/kg) or vehicle control as measured by half-maximal effective temperature (ET_50_) on O_2_ consumption rate for mice during acute exposure to various temperatures. **i** Effect of β_3_AR agonist on heat sensitivity of YS mice (*n* = 106) as measured by ET_50_ on survival rate of mice after acute exposures. All mice were within the controlled age range (8.8 ± 1.0 week old) at the time of study. *P* values are indicated as analyzed by ordinary one-way analysis of variance (ANOVA) with Tukey’s multiple comparisons test (**a**, **b**, **d**, **e**), and Mantel–Cox log-rank test (**c**, **f**), and *F*-test for differential ET_50_ of nonlinear regression with variable slope (**g**–**i**). Effects on heat sensitivity alteration in mice are established by comparing littermates of the same genotype with or without the genetic (**a**–**c**) or pharmacological (**d**–**i**) interventions. All statistical tests are two-sided. Data are represented as mean ± standard deviation (**a**, **b**, **d**, **e**), or asymmetrical 95% confidence non-linear best-fit curve (**g**–**i**). Source data are provided as a source data file.

To test the role of UCP1-mediated thermogenesis in the heat response of YS mice, we genetically ablated UCP1 in the YS mice by crossing UCP1-deficient mice (*Ucp1*^−*/*−^) with YS mice to generate YS/*Ucp1*^*+/*−^ mice. We confirmed that UCP1 levels in heterozygous mice were effectively reduced to approximately 65% and 55% of the UCP1-intact WT and YS mice at the protein level in brown fat, respectively (Supplementary Fig. 6a, b). The UCP1 deficiency was associated with reduced maximal VO_2_ (Fig. 4a) and VCO_2_ (Fig. 4b) upon heat challenge of the YS mice and improved their survival of the heat exposure (Fig. 4c) compared to the UCP1-intact YS littermate controls. YS mice completely devoid of UCP1 (YS*/Ucp1*^−*/*−^) also exhibited a significant improvement in survival but were not further protected compared to heterozygous YS*/Ucp1*^*+/*−^ mice (Supplementary Fig. 6c–e). These studies demonstrate that UCP1-dependent thermogenesis contributes to the heat response of the YS mice.

Brown fat is heavily innervated by sympathetic nerves, and responds to cold-sensing mechanisms in the central nervous system via the abundantly expressed β_3_-adrenergic receptors (β_3_AR)^52^. We pharmacologically modulated the UCP1 activity using sympathomimetic agents specific to brown and beige fat selective β_3_AR. We found that acute administration of a β_3_AR antagonist (L748337, 1 mg/kg)^53^ significantly decreased the hypermetabolic response (Fig. 4d, e) and improved the survival of the YS mice compared to vehicle-treated YS littermate controls (Fig. 4f). In contrast, the administration of a β_3_AR agonist (BRL37344, 1 mg/kg)^53^ resulted in a marked sensitization of mice to heat as assessed by changes in maximal VO_2_ in mice exposed at different temperatures (Fig. 4g, h). BAT activation with β_3_AR agonist reduced the half-maximal effective temperature (ET_50_) for the increase in VO_2_ in both WT (Fig. 4g) and YS mice (Fig. 4h) compared to vehicle-treated littermate controls, despite the intrinsically high heat sensitivity of YS mice at baseline. Notably, the β_3_AR agonist significantly decreased ET_50_ for survival of the YS mice after the heat exposure (Fig. 4i). The effects of brown fat activation were also confirmed in YS mice treated with an alternative β_3_AR agonist (CL316243, 1 mg/kg), which showed significantly exacerbated heat-induced hypermetabolic response even at modest sub-triggering temperatures (Supplementary Fig. 6f, g).

To test the possibility that the β_3_AR agonists and antagonists were altering RYR1 activity in skeletal muscle, we tested the effects of a β_3_AR agonist (CL 316,243, 10 µM) and a β_3_AR antagonist (SR 59230A, 10 µM) on heat-induced increases in resting Ca^2+^ levels in *flexor digitorum brevis* (FDB) fibers and on heat-induced increases in basal tension in soleus muscles (Supplementary Fig. 6h–j). Treatment of FDB fibers with either β_3_AR agonist or antagonists did not significantly alter the enhanced temperature-dependent increase in resting Ca^2+^ in FDB fibers from the YS mice (Supplementary Fig. 6h). In addition, neither β_3_AR agonists nor β_3_AR antagonists altered elevation in basal tension at 37 °C in muscle from YS compared to WT mice (Supplementary Fig. 6i, j). These findings suggest β_3_AR modulators alter temperature sensitivity of the YS and WT mice primarily via their effects on brown fat rather than on skeletal muscle.

Recent studies have shown that non-shivering thermogenesis (NST) also occurs in skeletal muscle and involves sarcolipin (SLN) uncoupling of Ca^2+^ uptake via the SR/ER Ca^2+^ ATPases (SERCA) from ATP hydrolysis, leading to increased heat production and decreased Ca^2+^ uptake into the SR^27–29,54,55^. As we have previously demonstrated^56,57^, Ca^2+^ uptake into the SR at room temperature, assessed by rate of return of cytosolic Ca^2+^ to baseline, was not decreased in FDB fibers from YS compared to WT mice, suggesting that the ability of SERCA to pump Ca^2+^ is not inhibited in fibers from YS compared to WT mice. In addition, we found no differences in *Sln* mRNA levels in the soleus or EDL muscles of YS compared to WT mice (Supplementary Fig. 6k). However, consistent with compensatory upregulation of *Sln* in UCP1-deficient mice^54^, *Sln* mRNA was significantly upregulated in the muscles of the WT/*Ucp1*^+/−^ and YS/*Ucp1*^+/−^ mice relative to UCP1-intact littermate controls (Supplementary Fig. 6k). While we cannot eliminate a possible contribution of NST in skeletal muscle, our results suggest that brown fat thermogenesis is a greater contributor to the heat sensitivity of the YS mice.

Adaptive thermogenesis alone is not causative of the heat sensitivity of the YS mice, but rather, further sensitizes the YS mice to heat by increasing basal body temperature. When treated with dantrolene, which blocks SR Ca^2+^ release, the YS mice respond like WT mice and did not die following heat challenge at triggering temperature (37 °C), even if BAT is activated with a β_3_AR agonist (Supplementary Fig. 6l). However, we found a small but significant increase in the β_3_AR agonist-induced hypermetabolic response in YS mice compared to their littermate controls (Supplementary Fig. 6m), suggesting a greater adipose tissue thermogenic capacity in YS mice.

### Increased BAT activity and white adipose tissue browning in YS mice

**Fig. 5:**
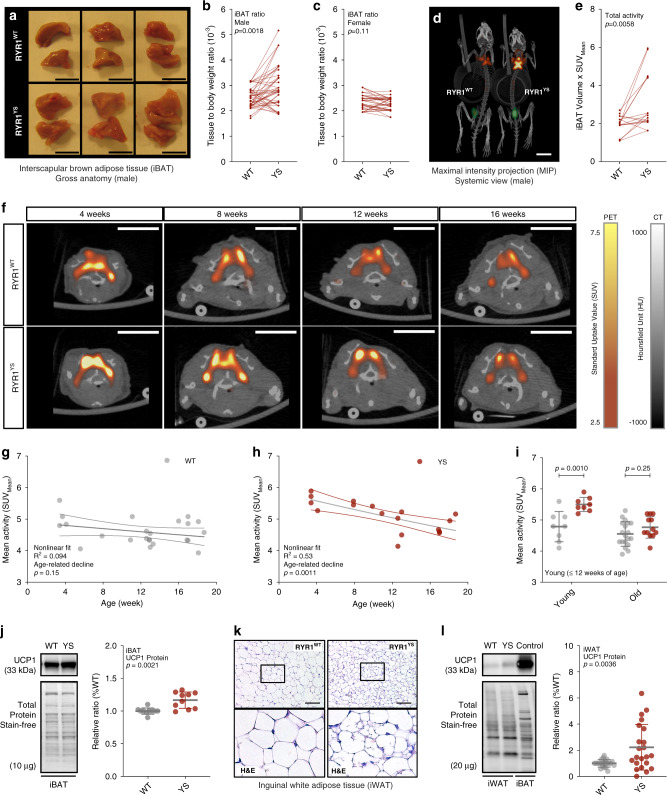
Elevated brown adipose tissue thermogenic capacity and white adipose tissue browning in Y524S mice. **a** Gross anatomy for the interscapular brown adipose tissue (iBAT) from WT and YS littermates. **b**, **c** Tissue to body weight ratio of iBAT resected from littermate pairs of WT and YS in male (*n* = 42 pairs, **b**) and female (*n* = 36 pairs, **c**) mice. **d** Representative PET-CT scan of WT and YS littermate. **e** Quantification of total iBAT activity in WT and YS littermates (*n* = 16 pairs). **f** Representative PET-CT scans from the interscapular region of WT and YS mice from various age groups. **g**–**i** Age related decline of body weight-adjusted total iBAT activity in WT (*n* = 28, **g**) and YS (*n* = 17, **h**) mice, and comparison between WT and YS within age groups (**i**). **j** Relative UCP1 protein levels in iBAT from WT (*n* = 10) and YS (*n* = 10) littermates. **k** H&E stain images of subcutaneous inguinal white adipose tissue (iWAT) from WT and YS littermate. Morphological changes are consistent across three independent littermate pairs. **l** Relative protein levels of UCP1 in iWAT from WT (*n* = 22) and YS (*n* = 22) littermates, using iBAT as a positive control. *P* values are indicated as analyzed by paired *t* test (**b**, **c**, **e**), *F*-test for deviation from zero-rate constant of nonlinear regression (**g**, **h**), and two-way ANOVA with Sidak’s multiple comparisons test (**i**), and Welch’s *t* test (**j**, **l**). All statistical tests are two-sided. *R*^2^ values are indicated to quantify goodness-of-fit to nonlinear regression with variable slope (**g**, **h**). Data are represented as mean ± standard deviation (**i**, **j**, **l**), or asymmetrical 95% confidence intervals (CI) from nonlinear best-fit curve (**g**, **h**). Mice were within the controlled age range (male: 12.0 ± 0.3-week-old (**b**), and female: 11.9 ± 0.3 week old (**c**)) at the time of study, except for the age-dependent experiments (**g**–**i**). Display ranges of standard uptake value (SUV) for PET and Hounsfield unit (HU) for CT are as indicated (**f**). Scale bars are 5 mm from the gross anatomy images (**a**), 10 mm from the PET-CT scans (**d**, **f**), and 100 μm from the histological images (**k**). Source data are provided as a source data file.

We found that male YS mice had significantly greater BAT mass in the interscapular region (iBAT) than their male WT littermates (Fig. 5a, b). In contrast, female YS mice had the same amount of BAT mass as their female WT littermates (Fig. 5c). To quantitatively assess the activity of brown fat in mice, we used integrated positron-emission tomography and computed tomography (PET/CT) functional imaging, which maps the metabolic activity by measuring the uptake of the glucose analog ^18^F-fluorodeoxyglucose (18F-FDG) systemically in tissues. The mice were precooled at 4 °C for 4 h prior to imaging to enable detection of 18F-FDG uptake. The active volume and total activity for 18F-FDG in and around the interscapular region were significantly higher in the YS mice compared to their WT littermate controls (Fig. 5d, e, Supplementary Fig. 7a–d) with no difference in lean mass (Supplementary Fig. 7e). Moreover, as indicated by pharmacokinetic analysis of 18F-FDG uptake (Supplementary Fig. 7f–h), we found a faster rate of decline in the FDG signal in YS mice. Because of the combined effect of precooling, which activates BAT, and isoflurane, a volatile anesthetic which can trigger an MH response, a portion of the YS mice had MH responses and died during the imaging (Supplementary Fig. 7i). Despite isoflurane effects on FDG uptake arising from the MH response of some of the mice, we detected a significant increase of total iBAT activity in surviving YS mice, suggesting that the detected iBAT activity is an underestimate of the baseline level without the isoflurane exposure. Consistent with the pediatric predominance in brown fat activity in humans and the associated enhanced heat sensitivity in pediatric individuals with *RYR1* variants associated with MHS, we found that brown fat metabolic activity was the highest in young YS mice and declined gradually with age in both WT and YS mice (Fig. 5f–i). The increased BAT activity was also consistent with an increase in UCP1 expression in iBAT (Fig. 5j).

Browning of subcutaneous white adipose tissue (WAT) also contributes to thermogenesis in mice and humans^58,59^. We found increased multilocular adipocytes in the subcutaneous inguinal WAT (iWAT) of the YS mice compared to that of WT littermate controls (Fig. 5k), resembling the metabolically more active brown adipocytes. Despite the variable UCP1 expression among mice and the tissue heterogenicity of UCP1 within iWAT (Supplementary Fig. 8a, b), we found the amount of UCP1 was increased in YS compared to WT littermates at both protein and mRNA levels (Fig. 5l, Supplementary Fig. 8c). Browning of the white fat in YS mice was further supported by the significant elevations in PPAR-γ coactivator-1 alpha (PGC1α) and mitochondrial markers, including cytochrome C (CYC), mitofusin-2 (MFN2), sirtuin-3 (SIRT3), and manganese superoxide dismutase (MnSOD) in the iWAT of YS mice compared to that of WT littermates (Supplementary Fig. 8d, e). These results indicate that brown fat activity and white fat browning are both increased in YS mice.

### Circulating lactate is increased in the YS mice

**Fig. 6:**
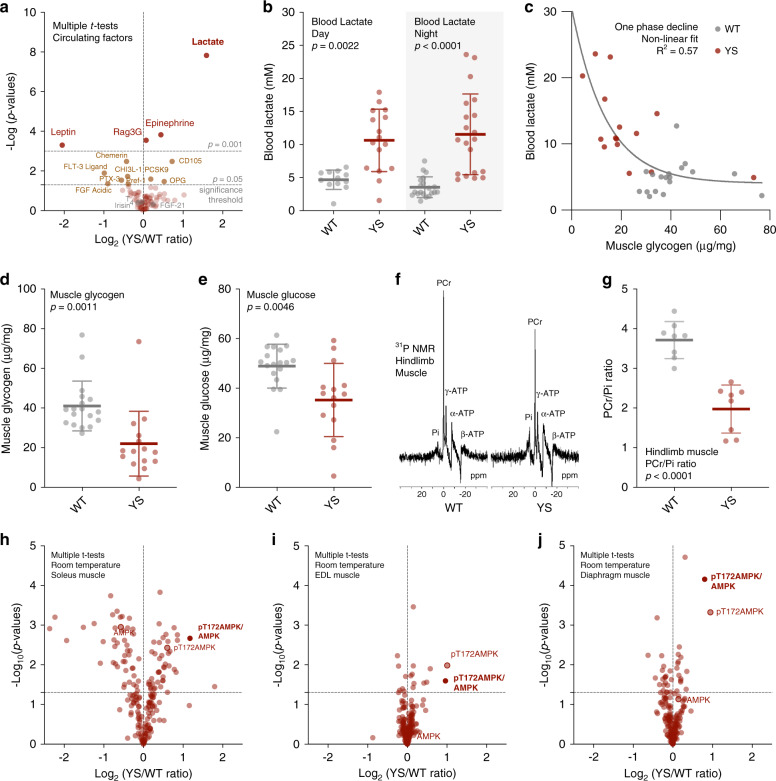
Increased circulating levels of skeletal muscle-derived lactate in the Y524S mice. **a** Volcano plot comparing the levels of circulating factors in WT (*n* = 4) and YS (*n* = 4) littermates. **b** Blood lactate concentration of WT and YS littermates as measured in filtered serum collected during day (WT *n* = 12, YS *n* = 16) and night (WT *n* = 20, YS *n* = 20) time. **c** Relationship of gastrocnemius muscle glycogen and blood lactate levels in WT (*n* = 18) and YS (*n* = 15) mice. **d**, **e** Muscle glycogen (**d**) and glucose (**e**) concentration as measured in gastrocnemius muscle isolated from WT (*n* = 18) and YS (*n* = 15) littermates. **f** Representative ^31^P nuclear magnetic resonance (NMR) spectra of hindlimb muscles measured in vivo from WT and YS littermate. **g** Quantification of phosphocreatine to inorganic phosphate ratio (PCr/Pi) based on analysis of the ^31^P NMR spectra from WT (*n* = 8) and YS (*n* = 8) hindlimb muscles. **h**–**j** Volcano plot comparing the level of proteomic changes in soleus (**h**), EDL (**i**), and diaphragm (**j**) muscle of WT (*n* = 3) and YS (*n* = 3) littermates. *P* values are indicated as analyzed by two-side unpaired *t* test without adjustment for multiple comparisons (**a**, **h**–**j**), ordinary one-way analysis of variance (ANOVA) with Tukey’s multiple comparisons test (**b**), and Welch’s *t* test (**d**, **e**, **g**). All statistical tests are two-sided. *R*^2^ values are indicated to quantify goodness-of-fit to nonlinear regression with variable slope (**c**). Data are represented as mean ± standard deviation (**b**, **d**, **e**, **g**). Mice were within the controlled age range (10.0 ± 1.8 week old) at the time of study. Source data are provided as a source data file.

The skeletal muscle Ca^2+^ release channel (RYR1) is central to muscle excitation–contraction coupling. To explore the mechanism by which a mutation in *Ryr1* alters the amount and activity of BAT, we assessed the levels of a wide range of circulating factors in the serum or urine of YS mice compared to their WT littermate controls. We did not detect significant changes in most known browning factors, including fibroblast growth factor 21 (FGF21), thyroid hormone (T_4_), or irisin (Fig. 6a). However, we found a significant increase in circulating lactate (Fig. 6a). Specifically, we found lactate levels were substantially elevated in the serum of YS compared to WT mice both during the day and the night (Fig. 6b) despite significant circadian fluctuations in tail artery blood lactate levels (Supplementary Fig. 9a–c).

Lactate is the end product of glycolysis and increased circulating levels of lactate are associated with increased muscle glycogen breakdown with exercise^60^. Increased intracellular Ca^2+^ due to a leaky RYR1 channel can activate multiple Ca^2+^ sensitive pathways including glycogenolysis via Ca^2+^-bound calmodulin (CaCaM), which acts as the *δ* subunit of glycogen phosphorylase kinase (PHK) in muscle^61^. To demonstrate that the elevated circulating lactate is the result of skeletal muscle glycogenolysis and glycolysis, we assayed *gastrocnemius* muscle and found that blood lactate inversely correlated with muscle glycogen (Fig. 6c) and both glycogen and glucose levels were significantly decreased in muscle of the YS compared to WT mice in the absence of exercise (Fig. 6d, e). In contrast to the decreased levels of glucose and glycogen in muscle, we detected a significant increase in hepatic glucose levels with no difference in blood glucose or hepatic glycogen in the YS mice, indicating normal hepatic gluconeogenesis following the uptake of circulating lactate into the liver (Supplementary Fig. 9d–g). These results suggest that the primary source of the elevated circulating lactate in YS mice is skeletal muscle glycogenolysis and glycolysis, and not a defect in hepatic lactate clearance.

Increases in cytosolic Ca^2+^ levels also activate the SERCA to restore SR luminal Ca^2+^ levels and actomyosin ATPase for myofilament cross-bridge cycling functions. Glycolysis can be activated by the energy-sensing AMP kinase (AMPK) in response to increased energy demand^62^. To probe muscle energetics in the YS mice, we used ^31^P nuclear magnetic resonance (NMR) spectroscopy to detect changes in phosphocreatine (PCr), ATP, and inorganic phosphate (Pi) in the hindlimbs of WT and YS littermate mice in vivo (Fig. 6f). PCr phosphorylates ADP to ATP to prevent major changes in muscle ATP levels^63^. We found a significant decrease in the PCr/Pi ratio in YS mice (Fig. 6g, Supplementary Fig. 9h, i) in the absence of a net change in ATP (Supplementary Fig. 9j), suggesting that PCr is efficiently replacing utilized ATP. ATP utilization increases AMP/ATP ratios which allosterically enhance the phosphorylation of Thr172 in the AMPKα subunit^64^. The Ca^2+^/calmodulin-dependent protein kinase kinase β (CAMKKβ), activated by increased cytosolic Ca^2+^ and Ca^2+^CaM and by phosphorylation^65^, phosphorylates Thr172 on AMPKα^66–68^. Consistent with the increased resting cytosolic Ca^2+^ levels, CaMKK2 phosphorylation was increased in the muscle of the YS mice (Supplementary Fig. 10a, b). Using reverse phase protein arrays (RPPA), we found a substantial increase in pT172-AMPK/AMPK in soleus, EDL, and diaphragm of the YS compared to WT mice (Fig. 6h–j, Supplementary Data 3) under baseline conditions. AMPK is activated by muscle contraction and exercise^69^. Increased AMPK activation suggests that muscles of the YS mice have increased metabolic activity, possibly due to transient muscle contractures. Such an increase in muscle activity would lead to increased lactate, even in the absence of elevated environmental heat.

Since norepinephrine and epinephrine activate BAT thermogenesis^70^, we measured the levels of epinephrine and norepinephrine output in the urine of the YS and WT mice. Consistent with the positive association between circulating lactate and catecholamine levels in humans^71–73^, we detected a significant increase in the total 24-h excretion of epinephrine in the urine of YS mice compared to that of WT littermates with no difference in norepinephrine (Supplementary Fig. 10c, d). As a sympathetic activator, epinephrine increases both adipose tissue thermogenesis and skeletal muscle force production^74^, thereby amplifying the effect of the *Ryr1* mutation on both lactate production and thermogenesis.

### Lactate increases brown/beige adipogenesis and UCP1 levels in brown and WAT

**Fig. 7:**
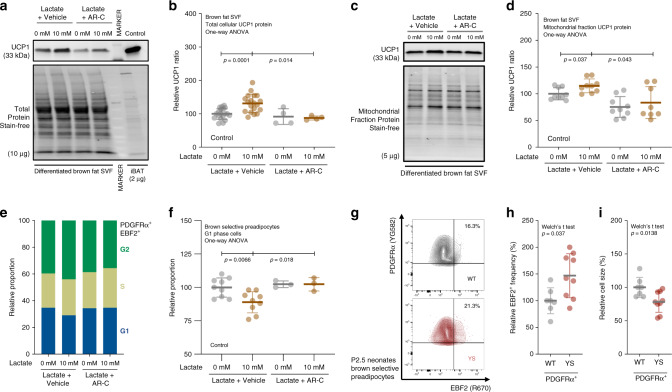
Lactate promotes brown adipogenesis in brown preadipocytes. **a**, **b** Representative immunoblots (**a**) and relative protein levels (**b**) for mitochondrial uncoupling protein (UCP1) in differentiation assay of interscapular brown adipose tissue (iBAT) stromal–vascular fraction (SVF) cells treated with vehicle control (*n* = 19), lactate (*n* = 19), monocarboxylate transporter (MCT1/2) inhibitor (*n* = 4) or both (*n* = 4). **c**, **d** Representative immunoblots (**c**) and relative protein levels (**d**) for UCP1 in mitochondrial fraction of differentiated iBAT SVF cells treated with vehicle (*n* = 9), lactate (*n* = 9), MCT1/2 inhibitor (*n* = 9) or both (*n* = 8). One data point (Lactate + ARC = 182.6% of control) identified as a statistical outlier (more than 2.04*σ* above the group mean) was excluded from the analysis based on the Grubb’s method. **e**, **f** Representative cell cycle distributions (**e**) and relative proportions (**f**) of G1 phase brown-selective preadipocytes in proliferation assay of iBAT SVF cells treated with vehicle (*n* = 9), lactate (*n* = 9), MCT1/2 inhibitor (*n* = 3) or both (*n* = 3). **g** Representative contour plots of platelet-derived growth factor receptor α-positive (PDGFRα^+^) preadipocytes in primary iBAT SVF cells isolated from P2.5 neonates of WT and YS littermate. **h**, **i** Relative early B cell factor-2-positive (EBF2^+^) frequency (**h**) and relative cell size (forward scatter, **i**) in PDGFRα^+^ preadipocytes in primary iBAT SVF cells from P2.5 neonates of WT (*n* = 7) and YS (*n* = 9) littermates. *P* values are indicated as analyzed by ordinary one-way analysis of variance (ANOVA) with Dunnett’s multiple comparisons test (**b**, **d**, **f**), and Welch’s *t* test (**h**, **i**). All statistical tests are two-sided. Data are represented as mean ± standard deviation. Relative protein levels are determined as protein levels normalized to vehicle (**b**, **d**, **f**) treated controls. Relative proportions are determined as proportions normalized to the level of wild-type littermate controls (**h**, **i**). Source data are provided as a source data file.

Both lactate and epinephrine elevate UCP1 levels in adipocytes^75,76^. Exercise also increases adipocytes progenitor populations in brown fat^77^. However, the exact mechanism of brown adipogenesis induced specifically by lactate is not known. To evaluate the effect of lactate on brown adipogenesis, we performed brown adipogenic assays on isolated primary stromal–vascular fraction (SVF) cells from iBAT isolated from pups at post-natal day 10 (P10). These cells were incubated with or without lactate (10 mM) and with and without an inhibitor of monocarboxylate transporters 1/2 (MCT1/2), AR-C155858 (AR-C, 0.1 µM). We found that UCP1 protein levels in differentiated SVF cells were significantly higher in cells treated with lactate compared to vehicle-treated controls, and this increase was prevented by AR-C (Fig. 7a, b). We also detected a significant increase in UCP1 levels in a mitochondrial fraction isolated from differentiated brown SVF cells and this increase was again blocked by AR-C (Fig. 7c, d). Lactate influx also transcriptionally increased brown adipogenic genes including *Ucp1*, *Ppargc1a*, and *Cidea* (Supplementary Fig. 11a–c). These findings suggest that lactate influx via MCTs is crucial for the lactate driven increase in *Ucp1* expression in cells differentiated from preadipocytes from BAT SVF.

As discussed above, YS mice displayed increased mass and volume of interscapular brown fat compared to their WT littermates (Fig. 5, Supplementary Fig. 7). We did not detect a significant difference in adipocyte size (Supplementary Fig. 11d–f), suggesting hyperplasia rather than hypertrophy of brown adipocytes in the YS mice. To determine if lactate was also contributing to hyperplasia of brown fat, we performed cell cycle analysis of proliferating brown fat SVF cells from WT mice by flow cytometry. Because these precursor cells in brown fat SVF could be a mixture of myogenic and non-myogenic origins, we designate them as brown/beige preadipocytes. Brown/beige preadipocytes in SVF were identified as double positive cells for preadipocyte marker platelet-derived growth factor receptor α (PDGFRα) and early B cell factor (EBF2), a selective marker for both brown and beige preadipocytes^78^. We found that brown/beige preadipocytes (PDGFRα^+^/EBF2^+^) were more proliferative than other cells in SVF (Supplementary Fig. 11g, h). Lactate treatment significantly increased the proportion of brown/beige preadipocytes in proliferative S/G2 phase and decreased proportion in quiescence G1 phase, and this proliferative effect of lactate was abolished by the MCT1/2 inhibitor (Fig. 7e, f). Consistent with the proliferative effect of lactate, we found an increase in EBF2^+^ frequency in the PDGFRα^+^ preadipocytes fraction in brown SVF cells from P2.5 YS neonates compared to their WT littermates (Fig. 7g, h). The PDGFRα^+^ preadipocytes from YS mice were also significantly smaller as indicated by the forward scatter (FSC) of flow cytometry, possibly due to active cell division (Fig. 7i). These findings suggest that lactate increases the proliferation of brown/beige preadipocytes in SVF, which is likely to underlie the increase in brown fat mass in the YS mice.

**Fig. 8:**
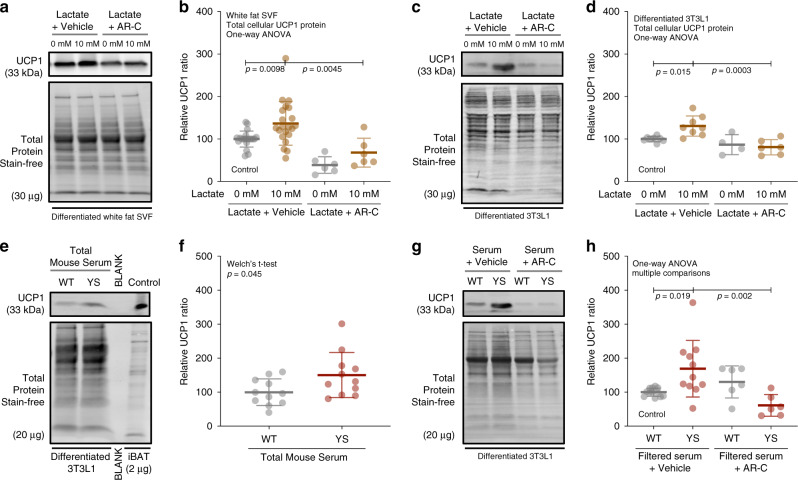
Lactate promotes brown adipogenesis in white preadipocytes. **a**, **b** Representative immunoblots (**a**) and relative protein levels (**b**) for mitochondrial uncoupling protein UCP1 in differentiation assay of inguinal white adipose tissue (iWAT) SVF cells treated with vehicle (*n* = 21), lactate (*n* = 21), MCT1/2 inhibitor (*n* = 6) or both (*n* = 6). **c**, **d** Representative immunoblots (**c**) and relative protein levels (**d**) for UCP1 in differentiation assay of 3T3-L1 preadipocytes treated with vehicle (*n* = 8), lactate (*n* = 8), MCT1/2 inhibitor (*n* = 4) or both (*n* = 6). **e**, **f** Representative immunoblots (**e**) and relative protein levels (**f**) for UCP1 in differentiation assay of 3T3-L1 preadipocytes treated with unfiltered serum (10%) from WT (*n* = 11) or YS (*n* = 11) mice. **g**, **h** Representative immunoblots (**g**) and relative protein levels (**h**) for UCP1 in differentiation assay 3T3-L1 preadipocytes treated with 10 kb-filtered serum (20%) from WT and YS mice, with (WT, *n* = 12, YS, *n* = 11) or without (WT, *n* = 6, YS, *n* = 6) the MCT1/2 inhibitor. *P* values are indicated as analyzed by ordinary one-way analysis of variance (ANOVA) with Dunnett’s multiple comparisons test (**b**, **d**, **h**), Welch’s *t* test (**f**). All statistical tests are two-sided. Data are represented as mean ± standard deviation. Relative protein levels are determined as protein levels normalized to vehicle (**a**, **b**) or wild-type mouse serum (**f**, **h**) treated controls. Source data are provided as a source data file.

As shown in Fig. 5, we also detected an increase in UCP1 levels in subcutaneous white fat of adult YS mice compared to their WT littermates. In addition to the effect of lactate on brown fat SVF cells, we tested the effect of lactate on SVF cells isolated from white fat and cultured 3T3L-1 preadipocytes. We found that lactate upregulated the expression of UCP1 and other browning markers across all systems, despite a variable expression at baseline among different sources of preadipocytes (Fig. 8a–d, Supplementary Fig. 12a–c). Again, the effect of upregulation of UCP1 levels was abolished with MCTs inhibition (Fig. 8a–d). These findings suggest that lactate influx via MCTs induces brown adipogenesis in both brown and subcutaneous WAT.

Finally, to determine if lactate is the primary circulating factor which induces brown adipogenesis in YS mice, we tested serum from WT and YS mice on 3T3L-1 preadipocytes and found that the serum from YS mice significantly increased the expression of UCP 1 compared to serum from WT littermate controls (Fig. 8e, f). To determine if this effect of YS serum on UCP1 expression was primarily due to lactate, we filtered WT and YS serum to remove components larger than 10 kDa, but retain lactate. Only small fragments of muscle proteins were left in the filtered sera, but none were increased in the YS compared to WT filtered serum (Supplementary Fig. 12d). The filtered serum from YS mice enhanced UCP1 expression to a greater extent than the filtered serum from WT littermates. The increase in UCP1 was prevented by MCTs inhibition (Fig. 8g, h). Collectively, these results suggest that skeletal muscle-derived lactate is not only applicable but also central to the increased brown/beige thermogenic capacity in YS mice.

### Elevated blood lactate levels in heat-sensitive MHS patients with RYR1 variants

**Fig. 9:**
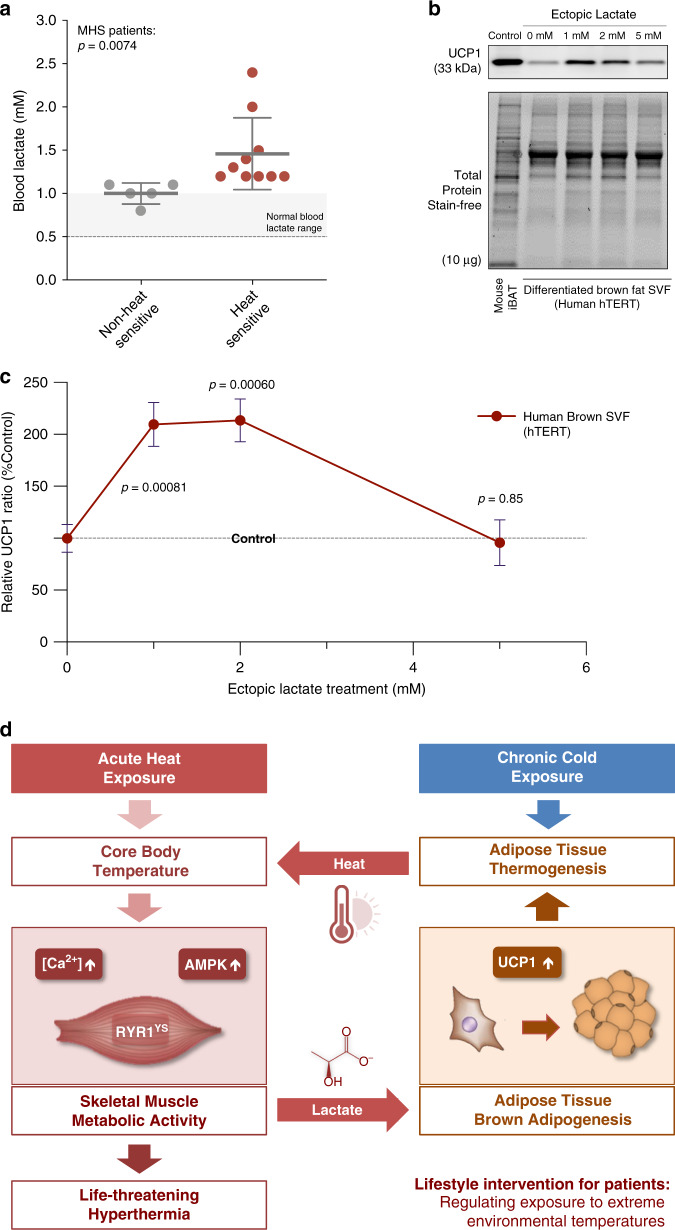
Increased blood lactate in heat-sensitive MHS patients and its effect on human preadipocyte brown adipogenesis. **a** Blood lactate concentration of non-heat sensitive (*n* = 5) and heat-sensitive (*n* = 10) MHS patients carrying RYR1 variants. **b**, **c** Representative immunoblots (**b**) and relative protein levels (**c**) for the mitochondrial uncoupling protein (UCP1) in differentiation assay of immortalized human preadipocytes from stromal–vascular fraction of brown adipose tissue treated with vehicle control and different dose of ectopic lactate (*n* = 5 each). **d** Proposed model of an adaptive thermogenesis enhanced feed-forward cycle of heat-induced heat response in susceptible patients. *P* values are indicated as Welch’s *t* test (**a**, **c**). All statistical tests are two-sided. Data are represented as mean ± standard deviation (**a**) and mean ± standard error of the mean (**c**). Relative protein levels are determined as levels normalized to the level of vehicle-treated controls (**c**). Source data are provided as a source data file.

Our findings with the YS mice raise the question of whether circulating lactate is associated with heat-sensitivity in MHS patients. We compared serum lactate levels in heat-sensitive patients with *RYR1* variants associated with MHS to asymptomatic carriers of the same variants and found that lactate was increased significantly in the serum of the heat-sensitive patients (Fig. 9a). The lactate levels in human serum are much lower than that detected in mice, raising the question of whether the small elevations detected could induce brown adipogenesis by upregulating UCP1 in human preadipocytes. In our attempt to quantify the dosage effect of lactate induced UCP1 expression in vitro, we tested the effects of low concentrations of lactate on UCP1 expression in immortalized human brown preadipocytes (hTERT). We found that lactate optimally increased UCP1 expression in this human cell line at 1–2 mM concentration range (Fig. 9b, c), indicating that even low concentrations of lactate drive UCP1 expression. Additional studies are needed to further evaluate the role of lactate and brown fat thermogenesis in individuals with *RYR1* variants associated with heat sensitivity.

## Discussion

The data presented here support a model in which both skeletal muscle and brown/beige adipose tissue contribute to the enhanced sensitivity of the YS mice to heat (Fig. 9d). The YS mutation in RYR1 increases SR Ca^2+^ leak and cytosolic Ca^2+^, which further sensitizes the RYR1 channel to activators^25^. The magnitude of the SR Ca^2+^ leak is increased by temperature^25,79^. Additional increases in heat can lead to sustained whole body muscle contractures, rhabdomyolysis and death from heart or kidney failure in the YS mice^24,25,80^. Increased cytosolic Ca^2+^ at rest activates Ca^2+^-sensing mechanisms and leads to muscle glycogenolysis and glycolysis, which elevates the circulating lactate. Circulating lactate is partially cleared by the liver and used for gluconeogenesis. However, muscle lactate production is sustained by the defective RYR1 channel via the effects of cytosolic Ca^2+^ on muscle metabolism. Circulating lactate induces brown/beige adipogenesis in fat by upregulating UCP1 levels. This increases the capacity of brown fat for adaptive thermogenesis systemically in the YS mice, especially upon chronic exposure to cold. Activation of BAT thermogenesis by pre-exposure to cold temperature or food intake causes a small elevation in body temperature that increases SR Ca^2+^ leak, creating a maladaptive feed-forward cycle of heat-induced heat response (Fig. 9d).

Pathogenic variants in the gene coding for the skeletal muscle Ca^2+^ release channel (*RYR1*) underlie both MHS and an MH-associated life-threatening response to heat^10–13^. In the most severe cases, both MH and the adverse heat response are characterized by sustained, whole body muscle contractures, leading to rhabdomyolysis and death from heart or kidney failure^77,81^. Responses in older adults with *RYR1* pathogenic variants are frequently in the form of exercise intolerance, myalgia, muscle cramps, and rhabdomyolysis^10–13,18,19,40,82,83^. Life-threatening responses, however, can occur in adults with these *RYR1* variants performing long duration, intense sports/exercise, or military training^84^. In addition, several commonly used medications (e.g., statins) have been associated with incidences of non-anesthetic MH-like responses in these individuals^85–87^. Drugs that cause hyperthermia^88^ could potentially trigger an MH-like episode and hence, body temperature is likely to play a critical role in the susceptibility to these MH-like responses to heat and/or exercise.

We conducted a retrospective cohort study and a systematic review to provide an up-to-date, comprehensive summary of the documented cases. As anticipated from previous reports^7,41,89^, we found that young age and male gender are significant risk factors for heat-sensitivity among individuals with *RYR1* variants associated with MHS. Children with MHS, especially infants, are the most vulnerable to heat-induced death. Children with these pathogenic variants are at higher risk than most children when exposed to high temperatures such as being left in a hot car. However, additional studies, including longitudinal clinical studies and controlled preclinical experiments, are needed to confirm these findings and to elucidate the underlying mechanisms for the pediatric and male predominance. Nevertheless, the identified risk factors should provide insights for prevention and management of the life-threatening response to heat in MHS patients.

Consistent with the potentially lethal heat response in children with MHS, we found that the YS mice display a life-threatening response to heat that is also more severe in young mice and displays male predominance. Since adaptive thermogenesis, the finely regulated bioenergetic process that produces heat in response to changes in environmental temperature or diet^90–92^, plays a crucial role in the regulation of body temperature, we assessed the contribution of adaptive thermogenesis to the heat sensitivity of the YS mice. BAT and skeletal muscle are the principal sites for adaptive thermogenesis. Our rationale to investigate a role for adaptive thermogenesis in the response to heat was that young children have more BAT and rely more on adaptive thermogenesis to maintain body temperature compared to adults^93,94^.

To evaluate the contribution of brown fat thermogenesis to the heat sensitivity of the YS mice, we used both genetic and pharmacological modulation of BAT. While the primary cause of the heat response of the YS mice is a temperature-dependent increase in cytoplasmic Ca^2+^ due to RYR1 Ca^2+^ leak^24,25,79^, we found that activation of brown fat thermogenic activity enhances the heat sensitivity of the YS mice. Under normal housing conditions, the YS mice display a small but significant increase in body temperature compared to their WT littermates. Body temperature before the 37 °C heat exposure correlates with the severity of the response of the mice to the 37 °C heat exposure. Factors that increase body temperature by activating adaptive thermogenesis, such as housing in the cold, food intake, and β_3_AR agonizts increase the probability that the YS mice will die in the heat exposure. In contrast, reducing UCP1 levels or blocking β_3_AR receptors increases the probability that the mice will survive the heat exposure. These data suggest that adaptive thermogenesis from either cold exposure or β_3_AR stimulation increases the sensitivity of the YS mice to heat and UCP1 expression is required for this sensitization.

While adaptive thermogenesis is essential for maintaining body temperature in all mammals, our data with the YS mice suggest that adaptive thermogenesis may be detrimental to MHS individuals with significant heat-sensitivity. Specifically, activation of adaptive thermogenesis can overcompensate when an individual with an *RYR1* mutation who has adapted to inside air conditioning goes outside into the heat. In this type of situation, adaptive thermogenesis could become maladaptive by further sensitizing these individuals with *RYR1* mutations to a heat response. Maintaining thermoneutral conditions and allowing adequate time to adjust to the hot outdoor temperatures before engaging in exercise or sports could decrease the risk of heat response in these individuals. In addition, individuals with these heat-sensitive *RYR1* mutations should take extra precautions to manage other sources of core temperature elevation, such as exercise, stress, infection, and thermogenic drugs.

An unexpected finding of our study was that the male YS mice have more BAT compared to their WT littermates, raising the question of the mechanism underlying the increased BAT. A number of browning factors have been previously identified^95,96^. While we found no increase in norepinephrine, we did detect a small, but significant, increase in epinephrine that could contribute to increased activation of BAT^97^. We also detected a large increase in circulating lactate in YS mice in the absence of heat exposure. Both lactate and epinephrine elevate UCP1 levels in adipose tissue^75,76,98^. To determine if the altered activity of the mutated RYR1 in skeletal muscle was responsible for the increase in circulating lactate, we measured glycogen levels in the muscle of YS and WT mice. We found substantial decreases in muscle glycogen in the muscle of the YS mice, suggesting that glycogen breakdown followed by glycolysis in muscle is the source of the circulating lactate. Our data suggest that glycogen breakdown and increased circulating lactate are being driven by increased muscle energy demands driven by increased cytosolic Ca^2+^. We did not find decreases in glycogen or glucose in the liver, suggesting that the liver is functioning normally.

Lactate plays diverse roles as a metabolic substrate and a signaling molecule^99^ but can also promote tumor growth by serving as an energy source^100,101^. Using primary cells from the stromovascular fraction of BAT from mice and a hTERT cell line, we found that lactate promotes brown adipogenesis by increasing the proliferation and differentiation of preadipocytes, but not by transdifferentiation of mature adipocytes. While the optimal approach to defining the effects of lactate on BAT would be in vivo studies, lactate should be tested during perinatal or even prenatal development when the stem cell population is the most abundant. Moreover, chronic pharmacological MCT inhibition in vivo in adult mice caused severe adverse effects systemically, likely due to the blocking of essential lactate-dependent metabolic processes^102,103^. The short window of opportunity during early development render feasible interventions in post-weaning pups difficult. Nevertheless, our study lays the foundations for future research to explore the effects of lactate on BAT. Development of novel genetic models, such as *Mct1*^*loxp*^ mice could be used to target lactate influx in a tissue specific manner^104^. Maternal delivery of lactate across placenta^105^ during pregnancy could potentially be used to test the effect of lactate in tissues during early development. Finally, 3D cultures of engineered organoids developed from adipose tissue SVF could serve as an intermediate system between in vitro and in vivo studies^106^.

While brown fat thermogenic activity is not the underlying cause of the heat-sensitivity, our findings suggest it is a sensitizing component of the feed-forward cycle of heat-induced hypermetabolic response in YS mice. Additional studies are needed to elucidate the mechanism by which lactate increases BAT in mice and to determine if lactate plays a similar role in individuals with *RYR1* variants associated with heat sensitivity.

In summary, while adaptive thermogenesis is essential for maintaining body temperature in all mammals, our results suggest that it can be detrimental to individuals with *RYR1* variants associated with MHS and heat-sensitivity. Specifically, activation of adaptive thermogenesis in response to the cold overcompensates upon transition to a warmer temperature. This overcompensation can render adaptive thermogenesis maladaptive. The common practices for setting indoor air-conditioning systems are largely geared toward human comfort and tend to overcompensate for elevated outdoor temperatures. For individuals with MHS and heat sensitivity this type of cold to hot temperature transition poses an acute risk. Simple and relatively minor lifestyle modifications such as slightly raising indoor temperatures (>21 °C, 70 °F) at home and not going outside in the summer immediately after eating could reduce the risk of a life-threatening response of children and young adult males with *RYR1* mutations to heat. Since body temperature plays a critical role in the response to heat, this vulnerable population should also be concerned about other factors that increase body temperature such as exercise in warm, humid environments, febrile illness, drugs that elevate body temperature and/or combinations of these factors^13^.

## Methods

### Human subjects

The institutional research ethics board (REB) at the University of Toronto approved this retrospective clinical study and waived the need for informed consent due to the retrospective nature of the study. Patients referred to the MH investigation unit (MHIU) at Toronto General Hospital between 1994 and 2019, who had significant heat sensitivity or heat-induced rhabdomyolysis AND carried a variant in *RYR1* were included. The significant heat sensitivity was defined as per patient’s report as suffering from heat exhaustion, occurrence of heat stroke or heat-induced rhabdomyolysis. Collected data included *RYR1* variants, gender, age at the time of assessment of heat sensitivity, description of symptoms, information on MH reaction if existed (clinical grading scale score), caffeine and halothane contracture results were available, blood lactate levels where available, and previous anesthetic history. The same information from family members, who carried the same genetic variant was collected. Clinical case reports published between 1980 and 2019 were systematically reviewed as previously described^38^ to identify additional eligible patients, using the preferred reporting items for systematic reviews and meta-analysis guidelines. The statement and checklists for the systematic reviews and meta-analysis are provided as Supplementary Notes 1–3. The institutional REB approval and consent wavier are provided as Supplementary Note 4. This waiver covers both consent to participate and consent to publish.

### Mice

The institutional animal care and use committee (IACUC) at Baylor College of Medicine approved all protocols for animal experiments in this study. All studies with mice were preformed according to ethical standards for research using animals. Mice are housed in 14-h light, 10-h dark cycle in a temperature-controlled environment (Supplementary Fig. 4) with 40–60% humidity in a pathogen barrier level 3 facility. The heating, ventilation, and air-conditioning (HVAC) systems of the housing facility are regulated and monitored by the building automation system (BAS). Indoor temperature, humidity, and animal health status were also monitored daily by animal care staff at the center for comparative medicine (CCM). Mice are given access to food and water ad libitum and provided with a nestlets enriched cage environment. PicoLab^®^ Select rodent diet 5V5R (LabDiet) is provided as the mouse normal chow diet, and chlorine dioxide treated water is provided as drinking water. Single housing conditions, noises, vibrations, and excessive handlings are kept at a minimum to reduce stress. Mice are weaned at 3 weeks of age and are tested at the specified age for each experiment. The mice with a heat-sensitive knock in allele in *Ryr1* (*Ryr1*^*Y524S/+*^, MGI: 3619253) were previously created by our lab^24,79^, and maintained on C57BL/6J genetic background. The mice with a cold-sensitive knockout allele in *Ucp1* (Ucp1^−/−^, MGI: 1857471) were obtained from the Jackson Laboratory (JAX^®^ 003124), kindly gifted to our lab by Dr. Ashley Chen from the Department of Pediatrics at Baylor College of Medicine and maintained on C57BL/6J genetic background. All in vivo data were retrieved from at least three independent experimental animal cohorts.

### Metabolic measurements

Energy balance parameters, including locomotor movement, ambulatory activity, feeding, drinking, and calorimetry, were simultaneously assessed by the comprehensive lab animal monitoring systems (CLAMS, Columbus Instruments). Twenty-four hour urine monitoring and collection, and additional monitoring for accumulative food and water intake were assessed in metabolic cages (TECNIPLAST). Lean body mass was assessed with dual-energy X-ray absorptiometry (DEXA, Lunar PIXImus). Baseline body temperatures were assessed with IPTT-300 temperature transponder (BioMedic Data Systems) for repeated measurements, and with RightTemp^®^ rectal thermometer (PhysioSuite^®^, Kent Scientific) for one-time measurements. Temperature acclimation to cold (4 °C) or warm (30 °C) condition was regulated by the environment-controlled enclosures (CLAMS-ENC70, Columbus Instruments). For experiments requiring temperature conditioning, mice were conditioned at the specified temperatures for 1 week prior to assessment for thermogenic capacity. To determine the effects of temperature conditioning on adaptive thermogenesis, core body temperatures were measured between 1 and 2 h after return to room temperature following the conditioning at either 4 or 30 °C. All temperature measurements were performed during the same time each day in the afternoon (2:00–4:00 pm) to reduce influences from circadian regulations.

### Heat response

To assess the heat response of mice, we exposed mice to an elevated environmental temperature at 37 °C (unless specifically indicated) for 15 min in a temperature-controlled enclosure (IITC Life Science). The heat exposure temperature was digitally regulated and additionally confirmed by a scientific mercury thermometer. Experimental mice (drug treated or *Ucp1-ablated*) and littermate control mice (vehicle-treated or *Ucp1-WT*) were exposed to heat in pairs simultaneously. To assess the heat sensitivity of mice over a temperature range, mice within a controlled age range were randomized into separate cohorts, and each cohort was exposed to a specified temperature. Oxygen consumption, carbon dioxide production, respiratory exchange ratio, and heat production were monitored and recorded during each heat exposure by Oxymax system for indirect calorimetry (IDC, Columbus Instruments). Core body temperature was assessed with RightTemp^®^ rectal thermometer (PhysioSuite^®^, Kent Scientific) before and after heat exposure to determine the extent of heat-induced hyperthermic response. For heat exposure experiment following physiological modulation of adaptive thermogenesis, mice were exposed to heat approximately 1–2 h after 1-week temperature conditioning at the specified temperature. To evaluate the effects of pharmacological modulation of β_3_ adrenergic receptors (β_3_AR), β_3_AR agonist, antagonist, or vehicles were injected to mice intraperitoneally 10 min prior to heat exposure. The agonist (BRL37344 and CL316243) and antagonist (L748337) for β_3_AR were reconstituted as stock solutions based on manufactures recommendations (TOCRIS) and diluted in saline to reach dosage of 1 mg/kg per mouse. Signs of stress and survival of mice were continuously monitored during heat exposure, hourly within 6 h, and every 6 h within 48 h after heat exposure by the experimenter. Mice were additionally monitored daily for health status by CCM. All heat exposure experiments were performed during the same time each day in the afternoon (2:00–4:00 pm) to reduce influences from circadian regulations.

### PET/CT imaging

The detection of brown fat activity in mice using a small animal dedicated PET and CT (micro-PET/CT) system with ^18^F-FDG (Cyclotope) has been previously described^107^. Specifically, mice were fasted overnight and exposed to cold temperature in 4 °C environment for 4 h before receiving 300 μCi of ^18^F-FDG via intraperitoneal (i.p.) injection. The mice remained in the cold conditions for an additional hour after FDG injection, for a total of 5 h prior to the imaging. CT scans (5 min) immediately followed by PET scans (30 min) were acquired for mice under anesthesia with isoflurane (2% in oxygen at 1.5 L/min) with the Inveon integrated PET/CT system (Siemens). Mutant mice and WT littermate control mice were imaged in pairs simultaneously. The quantification of brown fat volume and activity based on PET/CT functional imaging has been previously described^108^. Specifically, the acquired PET scans were reconstructed using OSEM3D reconstruction method and registered to the CT scan for attenuation correction and anatomical references. Major BAT was identified near the interscapular region, and adipose depots were confirmed with low-density regions in CT images of −300 to 100 in Hounsfield units. BAT was defined as having a metabolic activity above a threshold of standard uptake value (SUV) for ^18^F-FDG (SUV_LBM_ ≥ 3 g/mL). Total BAT activity was calculated as the product of brown fat volume and mean uptake activity (BAT_activity_ = BAT_volume_ × SUV_mean_). The pharmacokinetic coefficient for each animal is calculated as the slope of the linear regression for brown fat ^18^F-FDG SUV_max_ over time during the 30 min of imaging. Mice that had an MH response and died during imaging were not included in the analysis.

### NMR spectroscopy

Hindlimb muscles from mice were scanned on a Bruker 9.4 T magnetic resonance imaging system to measure in vivo PCr, Pi, and adenosine triphosphate (ATP levels). The ^31^P NMR spectra were collected using an Image Selected in vivo spectroscopy sequence with a TR = 2 s, NA = 192. Free induction decay was zero filled and a line broadening of 20 Hz was applied before the Fourier transformation. Analysis for ^31^P NMR spectra were done using Topspin within the Paravision 5.1 software. The α and β ATP peaks were used for the ATP assessments. Intracellular pH was determined using the equation:

1 $${\rm{pH}} = 6.75 + {\mathrm{log}}\left[ {\frac{{\left( {{\rm{CS}} - 3.27} \right)}}{{\left( {5.69 - {\rm{CS}}} \right)}}} \right],$$

where CS is the chemical shift between PCr and Pi.

### Muscle basal tension

Soleus muscle isolation and basal tension assessment were performed as described previously^24,25^. Briefly, intact soleus muscles were isolated from mice, mounted to a force transducer, and placed in a chamber containing oxygenated Krebs–Ringer buffer. A series of electrical stimulations for single twitches (train rate: 1 tps, train duration: 200 ms/train, stimulation duration: 0.2 ms/pulse, voltage: 20 V, resistance: 25 Ω) was applied to muscle at varying mounting length to adjust mounting to the its optimal length. Optimal length is defined as the length of muscle at which it generates the strongest isometric tension in response to twitch stimulation. To test the temperature-dependent increase in basal tension, temperature of the buffer was warmed from room temperature to 37.0 °C. Isometric basal tension was monitored by the force transducer for 15 min at 37.0 °C without electrical stimulations to identify maximum tension during the measurement period. Following the basal tension measurement, muscle length and muscle weight were measured to estimate physiological cross-sectional area (CSA) of the muscle, as:

2 $${\rm{CSA}} = \frac{m}{{l_o}} \times \rho,$$

where *m* is the measured muscle mass (*g*), *l*_*o*_ is the measured muscle optimal length (cm), and *ρ* is the mammalian muscle density constant (1.06 g/cm^3^)^109^]. Basal tension was calculated and expressed as specific stress based on maximum tension and CSA of the muscle, as:

3 $$S = \frac{F}{{\rm{CSA}}},$$

where *S* is the specific stress (N/cm^2^), *F* is the measured maximal tension (*N*), and CSA is the calculated muscle physiological CSA (cm^2^).

### Histology

Adipose tissues were paraffin-embedded, sectioned, and stained with Hematoxylin–Eosine (H&E) staining by the pathology and histology core at BCM. For immunofluorescent staining of mitochondrial uncoupling protein (UCP1), paraffin sections of inguinal fat from littermate mice were rehydrated, blocked in PBST (0.1 M phosphate buffer, pH 7.4 with 0.5 M NaCl and 0.5% Triton X-100) with 5% bovine serum albumin (PBSTB), and treated anti-UCP1 antibody (Abcam) at a dilution factor of 1:400 in the PBSTB. After washing twice with PBST for 10 min each, sections were treated with Alexa-Fluor 488 (Invitrogen) at a dilution factor of 1:200 at RT for 90 min followed by washing with PBST. At the final washing, DAPI (Invitrogen, 0.3 µM) was used for nuclei staining. Imaging was performed with the fluorescent microscope (Olympus IX81). Interscapular BAT from mice were embedded in OCT compound (Tissue-Tek^®^), frozen in 2-methylbutane precooled in liquid nitrogen, and frozen-sectioned with SHANDON cryostat microtome (Thermo Electron Corporation). These sections were blocked in the PBSTB, treated with anti-CD36 (NOVUS Biologicals) and anti-FATP1 (Santa Cruz, 1:400) The remaining steps are the same as above except for the fluorescent secondary Ab combinations: Alex Fluor 488 for FATP1 and Alexa Fluor 594 (Invitrogen) for CD36. Secondary antibodies were diluted at a dilution factor of 1:200 in PBS. The remaining procedures are the same with histology of paraffin sections. Microscopy was performed with image acquisition software for fluorescent microscopy (IX81, Olympus) and bright-field microscopy (NIS, Nikon).

### Preparation of primary SVF cells

Adipose tissue from interscapular or inguinal region were excised from mice for brown or white SVF, minced, and digested with 0.2% collagen type II (Worthington Biochemical) in SVF primary cells isolation buffer (100 mM HEPES, pH 7.4, 123 mM NaCl, 5 mM KCl, 1.3 mM CaCl_2_, 5 mM glucose, 1× penicillin/streptomycin, and 4% fraction V bovine serum albumin (Sigma)) for 30 min in shaking incubator set at 37 °C/150 rpm. During enzyme digestion in the shaking incubator, tubes were vortexed for 10 seconds every 5 min. After enzyme digestion, tubes with enzyme-digested solution were put on ice for 30 min to allow the mature adipocytes and lipid droplets to float. Clear part of enzyme-digested solution filtered through 70 µm cell strainer (BD Bioscience) and centrifuged at 700 × *g* for 10 min to pulldown SVF primary cells. After washing cell pellet with DMEM, SVF primary cell pellet was resuspended with prewarmed growth medium (DMEM with high glucose, 20% fetal bovine serum (FBS), and antibiotics) and plated on the cell culture dishes.

### Cell culture

The mouse preadipocyte cell line (3T3L1) and human immortalized BAT SVF cells (hTERTA41hBAT-SVF) (ATCC^®^) were cultured and maintained in the growth medium, high glucose DMEM (Gibco) with 10% FBS (ATCC^®^ for 3T3L1 and GenDEPOT (Opti-Gold) for human BAT SVF) and antibiotics (Gibco). For differentiation assays, cells were differentiated to induce UCP1 as described previously^110^. Confluent cells were treated with induction medium (differentiation medium (DMEM with 10% FBS) supplemented with dexamethasone (Sigma, 2 µg/ml), IBMX (Sigma, 0.5 mM), insulin (Sigma, 5 µg/ml), Rosiglitazone (Sigma, 1 µg/ml), and T_3_ (Sigma, 50 nM) for 2 days followed by changing into differentiation medium (DMEM with 10% FBS, IBMX, insulin, Rosiglitazone, and T_3_ up to indicated time period. Differentiating cells were treated with sodium-l-lactate (Sigma-Aldrich) at the indicated concentration, mouse serum of YS and WT littermates (10% instead of FBS), deproteinized plasma of YS and WT littermates (20% instead of FBS) and/or AR-C155858 (MedChemExpress, 0.1 µM) in the differentiation medium for 24–48 h. Cells were then lysed in RIPA buffer and loaded on 12% sodium dodecyl sulfate polyacrylamide gel electrophoresis (SDS-PAGE) gel for the Western blotting against UCP1 as an adipocyte marker. For proliferation assays, proliferating SVF cells were maintained in the growth medium and harvested prior to complete confluence, without adipogenic induction.

### Flow cytometry

Flow cytometry analysis of cells in primary SVF and cultured SVF cells in the iBAT and has been previously described^78,111^. For primary SVF cells, adipose tissue from interscapular region was excised from neonates at P2.5, minced in phosphate-buffered saline (PBS), digested with collagenase, and filtered through 70 µm cell strainer. For cultured SVF cells, adherent cells in dish were harvested by gentle scrapping without use of trypsin. Collected cells were immediately resuspended in standard flow cytometry (FC) staining buffer (containing 0.1% sodium azide, Sigma). Directly conjugated antibodies (anti-PDGFRα/CD140a-PE, 1:400, eBioscience) was added to cell pallet to stain surface antigens in dark at 4 °C for 45 min. After washing the cell pallets with FC staining buffer, 100 µl of 4% paraformaldehyde was added to fix the cells for 15 min, and cells were subsequently permeabilized with 100 µl 0.2% Triton X-100 (Sigma). After additional washing with FC staining buffer, directly conjugated antibodies (anti-EBF2-AlexaFluor647, 1:50, Bioss) and DAPI (1 µg/mL, BioLegend) was added to cell pallet to stain nuclear targets in dark at 4 °C for 45 min. After final washing, FC data for stained cells were acquired on the flow cytometer (LSRFortessa, BD) using FC acquisition software (FACSDiva, BD) within 24 h. Cell populations were screened for single cells by FSC, side scatter and DAPI gating, and analyzed for frequencies and fluorescent intensities (FlowJo, BD). Cell cycle analysis was performed based on the quantitative DAPI intensity for DNA content by the default univariate cell cycle model without constrains. A figure exemplifying gating strategy is provided in the Supplementary Fig. 15.

### Immunoblot analysis

Snap-frozen mouse tissues were homogenized with tissue homogenizer (Precellys) at 4 °C in a radioimmunoprecipitation assay (RIPA) buffer (150 mM NaCl, 1% NP-40, 0.5% sodium deoxycholate, 0.1% SDS, and 50 mM Tris pH 8.0) with freshly added protease inhibitor and phosphatase inhibitor cocktails (GenDEPOT). Adipose tissue samples were kept at 4 °C for additional 1 h, centrifuged at 4 °C for 20 min at 12,000 × *g*, and the aqueous lysates were collected to determine protein concentration with Pierce BCA protein assay (Thermo Scientific). Samples were diluted with 5× SDS sample buffer to 1× (2% SDS, 1% 2-mercaptoethanol, 10% glycerol, 0.004% bromophenol blue, and 50 mM Tris-HCl) and denatured at 95 °C for 5 min. Two to ten microgram of iBAT, 30 μg of iWAT, and 60 μg of muscle and cell lysates were resolved by SDS-PAGE, transferred to PVDF and blotted with specific primary antibodies at dilutions specified by manufacturer: anti-UCP1 (Abcam, AB10983, 1:1000) and anti-Tubulin (DSHB, 6G7, 1:500), anti-CYC (Cell Signaling, 1:1000), anti-PGC1α (Santa Cruz, 1:500). Fluorescent secondary antibodies were used to visualize protein bands on the Odyssey CLx Imager (LI-COR) and/or ChemiDoc^MP^ Imaging System (Bio-Rad), and protein band intensities were quantified by Image J. All antibodies used are listed in Supplementary Table 1.

### Gene expression analysis

Total RNA was extracted from mouse tissues using TRIzol reagent (Life Technologies). Total RNA (1 μg) was reverse transcribed into cDNA using the iScript cDNA Synthesis Kit (BIO-RAD). Gene expression assay was performed with the iQ SYBR Green Supermix (BIO-RAD) on ViiA 7 Real-Time PCR System (Applied Biosystems). Samples were run in technical triplicates, and specificity of the amplified products was confirmed by melting curve analysis. The slope of standard curves is used to estimate the PCR amplification efficiency. Relative gene expression of target genes was determined based on the reaction cycle threshold values calibrated for PCR efficiency using the Pfaffl method as^112^

5 $${\mathrm{Relative}}\,{\mathrm{expression}}\,{\mathrm{ratio = }}\frac{{\left( {{E}_{{\mathrm{target}}}} \right)^{\Delta {\mathrm{CT}}_{{\mathrm{target}}}{\mathrm{(control - sample)}}}}}{{\left( {{E}_{{\mathrm{reference}}}} \right)^{\Delta {\mathrm{CT}}_{{\mathrm{reference}}}{\mathrm{(control - sample)}}}}},$$

where *E*_target_ is the amplification efficiency of the target gene (*Sln*), Ereference is the amplification efficiency of the reference gene (*Rn18s*), and ΔCT is the cycle threshold deviation of the control (WT)—sample (mutant) for each gene. The following primer sets were used:

*Sln* 5′-GCACTAGGTCCTTGGCATGT-3′ (forward);

*Sln* 5′-ACTCAAGGGACTGGCAGAGA-3′ (reverse);

*Ucp1* 5′-AGGCTTCCAGTACCATTAGGT-3′ (forward);

*Ucp1* 5′-CTGAGTGAGGCAAAGCTGATTT-3′ (reverse);

*Ppargc1a* 5′-TATGGAGTGACATAGAGTGTGCT-3′ (forward);

*Ppargc1a* 5′-CCACTTCAATCCACCCAGAAAG-3′ (reverse);

*Cidea* 5′-TGACATTCATGGGATTGCAGAC-3′ (forward);

*Cidea* 5′-GGCCAGTTGTGATGACTAAGAC-3′ (reverse);

*Rn18s* 5′-GTAACCCGTTGAACCCCATT-3′ (forward);

*Rn18s* 5′-CCATCCAATCGGTAGTAGCG-3′ (reverse).

### Circulating factors

The MILLIPLEX^®^ (MMYOMAG-74K, EMD Millipore) mouse myokine assay was used to assess the serum level of a panel of myokines quantitatively, including brain-derived neurotrophic factor, interleukin 6 (IL6), interleukin 15 (IL15), fibroblast growth factor 21 (FGF21), fractalkine, follistatin-like protein 1 (FSTL1), myostatin (MSTN), irisin, leukemia inhibitory factor, oncostatin M, osteocrin, osteonectin. MILLIPLEX assay was performed with data acquisition platform (Luminex, EMD Millipore). Proteome Profiler^TM^ antibody arrays were used to assess the relative level of an additional panel of 111 soluble proteins in mouse serum (ARY028, R&D Systems). In addition, enzyme-linked immunosorbent assays were used to quantitatively determine the serum levels of IL6 (M60000B, R&D Systems) and total urinary excretion of epinephrine and norepinephrine (KA1877, Abnova). Clinical chemistries for thyroid hormone (T_4_) and other diagnostic mouse blood factors were assessed by the comparative pathology laboratory at BCM.

### Blood lactate assay

Blood was collected from the mice by retro-orbital bleeding using heparinized capillary tubes under anesthesia and centrifuged at 2000 × *g* at 4 °C for 10 min. The clear phase of plasma was filtered through a 10 kDa cut-off column and then measured for lactate by the commercial kit (BioVision) according to the manufacturer’s instruction. Briefly, the deproteinized plasma was diluted in the assay buffer in 1 to 100 on a 96-well plate, followed by adding reaction mixture. The plate was incubated at room temperature for 30 min in the dark. The optical density was measured in a microplate reader (BioTek) at 570 nm wavelength.

### Tissue glycogen and glucose assays

*Gastrocnemius* and liver were analyzed for glycogen and glucose levels using a commercial kit (BioVision). The assay was performed according to the manufacturer’s protocol. Briefly, each muscle was homogenized in 500 μl of deionized water with a bead beater (Bertin instruments). The homogenates were boiled for 10 min and centrifuged at 18,000 × *g* for 10 min at 4 °C. The clear supernatant was diluted in the hydrolysis buffer in 1–5 on a 96-well plate and incubated with hydrolysis enzyme mixture at room temperature for 30 min in the dark. The optical density at 570 nm wavelength was measured in a microplate reader (BioTek). The glycogen and glucose levels were normalized to the protein concentrations.

### RPPA

RPPA of skeletal muscle tissues were performed at the Functional Proteomics RPPA Core Facility the University of Texas MD Anderson Cancer Center. The following full description of RPPA protocol can be found at the core facility (https://www.mdanderson.org/research/research-resources/core-facilities/functional-proteomics-rppa-core/education-and-references.html): “Cellular proteins were denatured by 1% SDS (with Beta-mercaptoethanol) and diluted in five 2‐fold serial dilutions in dilution lysis buffer. Serial diluted lysates were arrayed on nitrocellulose-coated slides (Grace Bio Lab) by Aushon 2470 Arrayer (Aushon BioSystems). Total 5808 array spots were arranged on each slide including the spots corresponding to serial diluted: (1) Standard Lysates; (2) positive and negative controls prepared from mixed cell lysates or dilution buffer, respectively. Each slide was probed with a validated primary antibody plus a biotin‐conjugated secondary antibody. Only antibodies with a Pearson correlation coefficient between RPPA and western blotting of greater than 0.7 were used for RPPA. Antibodies with a single or dominant band on western blotting were further assessed by direct comparison to RPPA using cell lines with differential protein expression or modulated with ligands/inhibitors or siRNA for phospho- or structural proteins, respectively. The signal obtained was amplified using a Dako Cytomation–Catalyzed system (Dako) and visualized by DAB colorimetric reaction. The slides were scanned, analyzed, and quantified using a customized‐software to generate spot intensity. Each dilution curve was fitted with a logistic model (“Supercurve Fitting” developed by the Department of Bioinformatics and Computational Biology in MD Anderson Cancer Center, “http://bioinformatics.mdanderson.org/OOMPA”). This fits a single curve using all the samples (i.e., dilution series) on a slide with the signal intensity as the response variable and the dilution steps are independent variable. The fitted curve is plotted with the signal intensities both observed and fitted on the y-axis and the log2-concentration of proteins on the *x*-axis for diagnostic purposes. The protein concentrations of each set of slides were then normalized for protein loading. Correction factor was calculated by: (1) median centering across samples of all antibody experiments; and (2) median centering across antibodies for each sample.”

### Mass spectrometry

Peptides from the filtered serum samples were analyzed at the Mass Spectrometry Proteomics Core at BCM. Serum filtered through an Amicon 10 K centrifugal filter device (20 µl) was digested by trypsin (20:1 trypsin to peptide ratio) overnight at 37 °C. The digestion was stopped with 0.1% formic acid. Samples (4 µl) were analyzed using a nano-HPLC 1200 (Thermo Scientific) coupled to an Orbitrap Fusion^TM^ Lumos^TM^ Tribrid^TM^ (Fusion, Thermo Scientific) mass spectrometer. Samples were enriched using a 2 cm × 100 µm i.d. trap column with 1.9 µm Reprosil-Pur Basic C18 beads (Dr. Maisch HPLC GmbH, Germany) and separated with an 5 cm × 150 µm capillary column packed with 1.9 µm Reprosil-Pur Basic C18 beads. A 45-min discontinuous gradient of 2–24% acetonitrile and 0.1% formic acid at a flow rate of 800 nl/min was applied to column then electro-sprayed into the mass spectrometer. The MS1 spectrum was acquired in the Orbitrap with a resolution of 120,000 and *m/z* range of 300–1400. HCD fragmented MS2 spectra were acquired in ion-trap with rapid scan mode operated under the control of Xcalibur (software ver 4.1.50, Thermo Fisher Scientific) in data-dependent mode, acquiring fragmentation spectra of the top 30 ions. The MS/MS spectra were searched against target-decoy NCBI mouse RefSeq database (release January 2019, containing 48,156 entries) in Proteome Discoverer 2.1 interface (Thermo Fisher) with Mascot algorithm (Mascot 2.4, Matrix Science). Dynamic alterations in acetylation of the N-terminal amino acid and oxidation of methionines were allowed. The precursor mass tolerance was confined within 20 ppm with fragment mass tolerance of 0.5 Dalton and a maximum of two missed cleavages was allowed. Assigned peptides were filtered with 1% false-discovery rate using Percolator validation based on *q*-value. Shared peptides were split proportionally to corresponding unique peptide ratios. Peptides were grouped into gene products and shared peptides are split proportionally to corresponding unique peptide ratios as previously described^113^. Briefly, peptides were mapped to gene products (collapsing all protein isoforms that originate from the same genetic locus). Gene products are quantified by iBAQ^114^, with modification. For peptides that map to more than one gene product, peptide peak area is distributed in a weighted procedure based on unique peptide ratios. For each peptide shared across multiple gene products, the peptide peak area assigned to gene product is defined as:

6 $$s_A = \frac{{{\sum} {u_A} }}{{{\sum} {u_A} + {\sum} {u_B} + \ldots }},$$

where *u*_*i*,*A*_ is one of each unique-to-gene peptide for gene product *A*, and (*A*, *B*…,) are all gene products to which shared peptide *s* maps. For cases where no unique-to-gene peptides exist, shared peptide quantities are divided evenly across all gene products. Shared peptides were split proportionally to corresponding unique peptide ratios.

### Resting calcium in FDB fibers

Flexor digitorum brevis fibers (FDB) fibers were loaded with 5 µM Fura-2 AM (Thermo Scientific, Waltham, MA) for 30 min at room temperature in control Ringer’s solution (146 mM NaCl, 5 mM KCl, 2 mM CaCl2, 1 mM MgCl2, 10 mM HEPES, pH 7.4). Fibers were then treated for 30 min with dye-free Ringer’s solution supplemented with either 10 µM agonist (CL316243) or 10 µM antagonist (SR 59230 A). Fibers were then placed in a temperature-controlled chamber (Dagan, Corp., Minneapolis, MN) on the stage of an inverted epifluorescence microscope (Nikon Inc., Melville, NY) and alternatively excited at 340 and 380 nm (30 ms exposure per wavelength and 2 × 2 binning) using a Polychrome V monochromator-based illumination system (TILL Photonics, Munich, Germany). Fluorescence images were captured (510 nm emission) using a high speed, digital SENSICAM-QE CCD camera (Cooke, Romulus, MI). Ratio images (*R* = F340/F380) were generated using TILL Vision software (TILL Photonics, Pleasanton, CA) and mean resting values calculated using ImageJ (NIH) for data recorded at both 25 and 37 °C. For each condition, resting ratio images were first collected from several fibers in the dish while the temperature was held at 25 °C. The temperature of the bath was then equilibrated to 37 °C for 10–15 min and then the same fibers were re-imaged at the higher temperature. Fura-2 fluorescence ratios (*R* = F340/F380) were converted to free Ca^2+^ concentrations using an in situ calibration approach described previously^79^ and the following equation^115^

7 $$\left[ {{\mathrm{Ca}}^{2 + }} \right]{\mathrm{i}} = {K_{\mathrm{d}}} \, \times \, \beta \, \times \, \left( {\left( {{R} - {R_{\mathrm{min}}}} \right)/\left( {{R_{\mathrm{max}}} - {R}} \right)} \right),$$

where *K*_d_ is the Ca^2+^ affinity of Fura-2, *β* is the ratio of the 380-nm emission recorded under Ca^2+^-free and Ca^2+^ saturating conditions, *R*_min_ is the emission ratio under Ca^2+^-free conditions, and *R*_max_ is the emission ratio under Ca^2+^ saturating conditions. The values of *β*, *R*_min_, and *R*_max_ were determined experimentally. The *K*_d_ used was taken from the in vitro calibration of Fura-2 in the presence of 27 mg ml^−1^ of aldolase (428 nM^116^) and was assumed to be independent of the temperature in intact cells as shown previously^117^.

### Bioinformatics

The pathogenicity scores of the *RYR1* variants in patients with heat sensitivity are predicted with polymorphism phenotyping (PolyPhen) bioinformatics as described previously^43^. Mutant amino acid residues were aligned onto the linear residue spans of RYR1 subdomains as designated previously^44^, and visualized on the three-dimensional structure of the rabbit RYR1 homolog based on data from protein data bank (PDB ID: 5T15) with the Chimera program.

### Statistical analysis

Appropriate statistical analyses were applied as specified in the figure legends using Prism 8 (GraphPad Software). Sample sizes were determined based on power analysis of preliminary results or estimated based on similar previous studies when preliminary results were not available. All experiments were assessed in distinct samples, and the sample sizes (*n*) indicate the number of biological replicates. No samples were excluded from the statistical analysis unless specifically stated otherwise. Values were presented as mean ± standard deviation. *P* < 0.05 was required to be considered as statistically significant. All statistical tests are two-sided.

The following figures contain analysis of multiple parameters measured from the same experiments: Heat response: maxVO_2_, maxVCO_2_, core body temperature, and survival rate analysis (Figs. 1–4, SF.2); CLAMS study: VO_2_, VCO_2_, RER, heat, and activities (SF.3). Baseline core body temperature: core body temperature distribution and littermate pair analysis (Figs. 2 and 3); PET/CT imaging: brown fat volume, brown fat activity, and brown fat total volume analysis (Fig. 5, SF.7); Adipose tissue browning: UCP1, PGC1α, and other mitochondrial markers in immunoblot analysis (Fig. 5, SF.8); NMR imaging: PCr, Pi, and ATP analysis (Fig. 6, SF.9). High-throughput arrays: analysis for circulating factors from the cytokine array panel, myokine array panel, and clinical chemistry panel, analysis for muscle proteomics RPPA panels (Fig. 6, SF.10 and SF.12); High-throughput FC: cell cycle analysis for cell frequencies in G1, S, and G2 phases (Fig. 7, SF.11). Analysis of heat response for age dependence (Fig. 1), ambient/body temperature dependence (Figs. 2 and 3), and analysis of heat sensitivity (Fig. 4) are based on measurements from distinct animal subjects at the indicated conditions, and the same subjects were NOT heat-exposed and measured repeatedly.

### Reporting summary

Further information on research design is available in the Nature Research Reporting Summary linked to this article.

## Supplementary information

Supplementary Information

Description of Additional Supplementary Files

Supplementary Data 1

Supplementary Data 2

Supplementary Data 3

Supplementary Data 4

Supplementary Data 5

Reporting Summary

## Data Availability

All data generated or analyzed during this study are included in this published article and its supplementary files. Source data are provided with this paper. The mass spectrometry data have been deposited via the Mass Spectrometry Interactive Virtual Environment (MassIVE) repository. Source data are provided with this paper and open for the public: MSV000085803. Diagnostic MH mutations are obtained from European Malignant Hyperthermia Group (www.emhg.org) and are publicly available. Structures of RYR1 are generated based on data from protein data bank (PDB ID: 5T15) and are publicly available. Source data are provided with this paper.

## Source data

Source Data

## Code availability

This paper does not report custom code. Source data are provided with this paper.
