# Supplementary material for: Adaptive thermogenesis enhances the life-threatening response to heat in mice with an Ryr1 mutation
Source: Nat Commun. 2020 Oct 9;11:5099. doi: 10.1038/s41467-020-18865-z (PMC7547078; doi:10.1038/s41467-020-18865-z)
Supplement: Supplementary file 1 — Supplementary Information [file 41467_2020_18865_MOESM1_ESM.pdf]

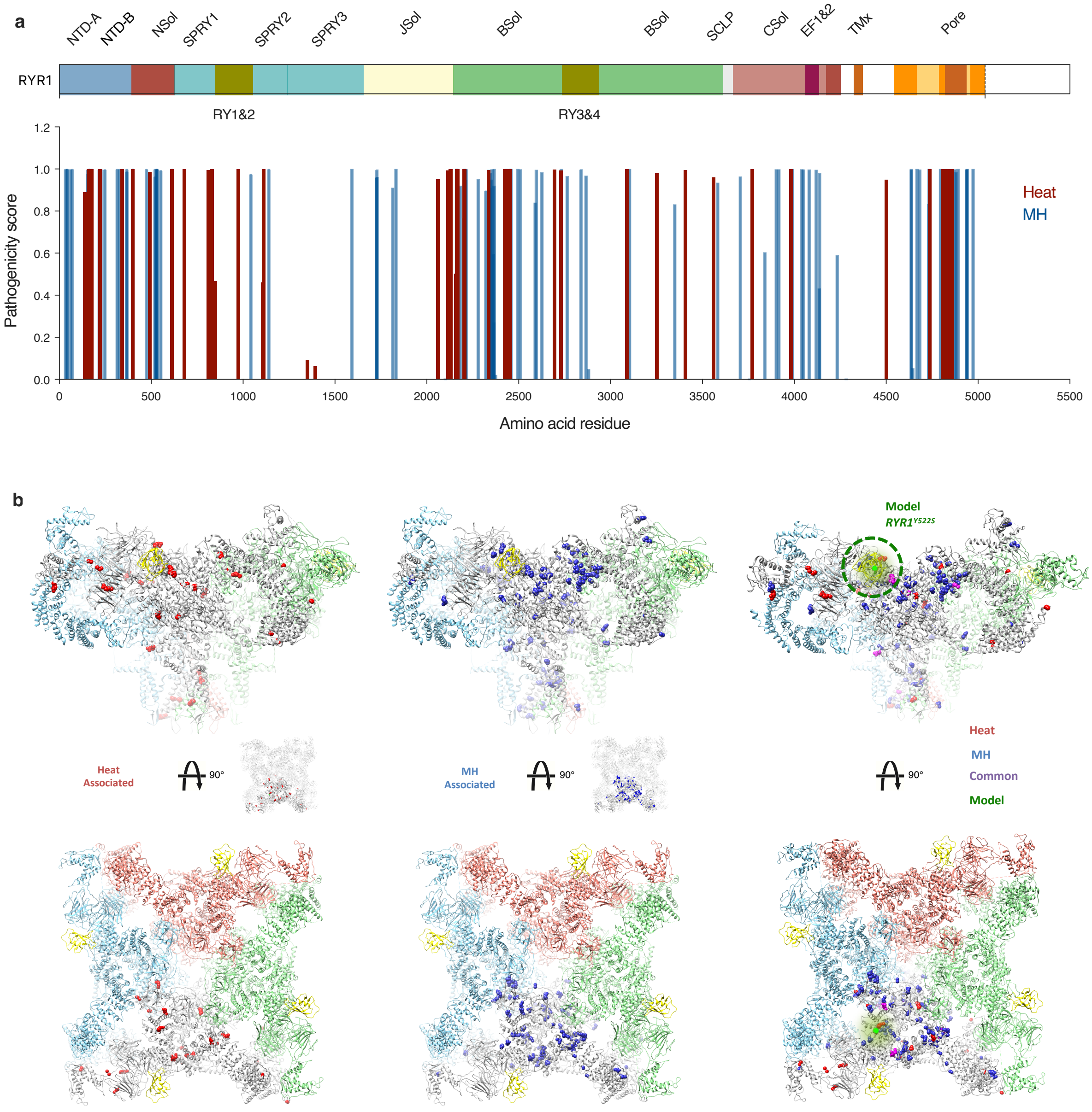

Supplementary Figure 1. Pathogenicity of *RYR1* variants associated with MH and heat-sensitivity. (**a**) The positions of mutant amino acid residues associated with anesthetic-induced malignant hyperthermia susceptibilities (MHS) and MH-like heat sensitivities in 2-dimensional RYR1 structural domains. (**b**) Positions of mutant amino acid residues associated with MHS and heat sensitivities in 3-dimensional structure of the rabbit homologue of RYR1. Residue spans of RYR1 subdomains are defined as previously designated [PMID: 27662087]: N-terminal domains A (NTD-A), N-terminal domains B (NTD-B), N-terminal solenoid (NSol), SP1a/ryanodine receptor domain 1 (SPRY1), RYR repeats 1 and 2 (RY1&2), SP1a/ryanodine receptor domain 2 (SPRY2), SP1a/ryanodine receptor domain 3 (SPRY3), junctional solenoid (JSol), bridging solenoid (BSol), RYR repeats 3 and 4 (RY3&4), shell-core linker peptide, CaM and JSol binding sites (SCLP), core solenoid (CSol), EF-hand pair (EF1&2), auxiliary transmembrane helices (TMx), pseudo voltage sensor domain (pVSD), helical-bundle domain between S2 and S3 (S2S3), channel pore domain (Pore), cytoplasmic extension of S6 (S6c), and C-terminal domain (CTD). Diagnostic MH mutations are obtained from European Malignant Hyperthermia Group (*www.emhg.org*). Structures of RYR1 are generated based on data from protein data bank (PDB ID: 5T15). Source data are provided as a Source Data file.

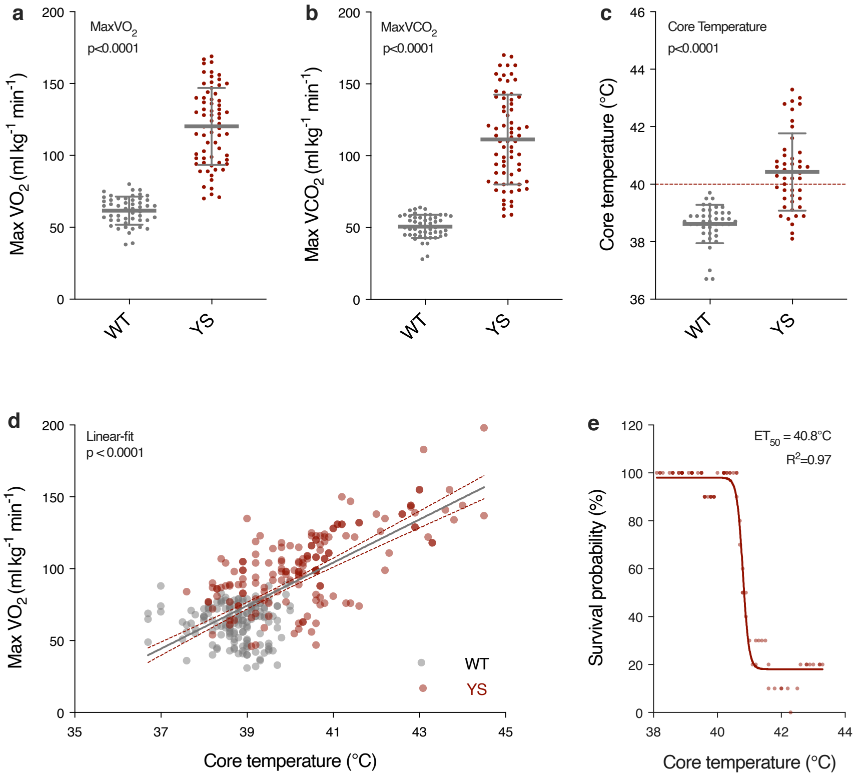

Supplementary Figure 2. Heat-induced hypermetabolic response leads to death in Y524S mice. (**a,b**) Maximum O_2 consumption ($MaxVO_2$, **a**) and CO_2 production ($MaxVCO_2$, **b**) rate of WT (n=50) and YS (n=70) during the acute environmental heat exposure for 15 minutes at 37°C. (**c**) Core body temperature of WT (n=41) and YS (n=45) mice after acute heat exposure. (**d**) Relationship of post-heat challenge core body temperature and $MaxVO_2$ during acute 37°C heat challenge in WT (n=67) and YS (n=213) mice. (**e**) Effect of post-heat challenge core temperature on estimated survival probability of YS mice (n=77) after acute heat challenge. All mice were within the controlled age range (8.9 ± 0.9-week-old) at the time of study. All mice were housed at room ambient temperature (20.2 ± 0.4°C) prior to heat challenge. P values are indicated as analyzed by Welch's t-test (**a-c**), and F-test for deviation from zero-slope of linear regression (**d**). All statistical tests are two-sided. R^2 values are indicated to quantify goodness-of-fit to non-linear regression with variable slope (**e**). Survival probability of each mouse is estimated based on the survival rate from 10 mice in the respective core body temperature (**e**) subgroups. Effect of post-heat challenge core body temperature on heat challenge survival is measured by half-maximal effective core body temperature (ET_{50}) on the estimated survival probability (**e**). Data are represented as mean ± standard deviation (**a-c**), or asymmetrical 95% confidence intervals (CI) from linear best-fit line (**d**). Source data are provided as a Source Data file.

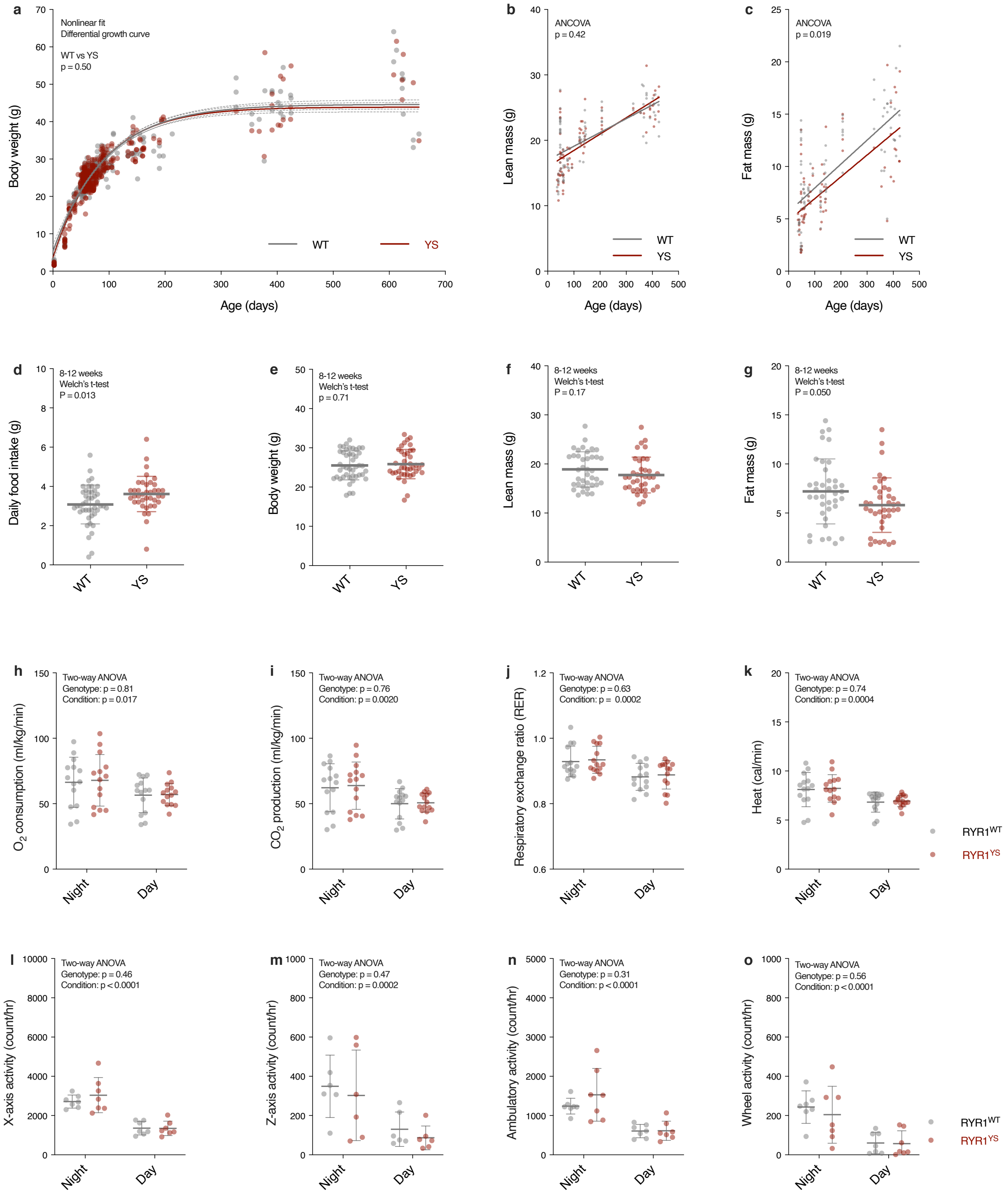

Supplementary Figure 3. Basal energy metabolism in Y524S mice. (a) Body weight of WT (n=391) and YS (n=452) mice from various ages. **(b,c)** Lean mass (**b**) and fat mass (**c**) of WT (n=91) and YS (n = 77) mice from various ages. **(d,e)** Daily food intake (**d**) of WT (n = 44) and YS (n = 39) mice and their body weight (**e**) from controlled age range. **(f,g)** Lean mass (**f**) and fat mass (**g**) of WT (n=38) and YS (n=37) mice from controlled age range. **(h-k)** Indirectly calorimetry measured by comprehensive laboratory animal monitoring system (CLAMS) for WT (n=14) and YS (n=14) littermates in oxygen consumption (**h**), carbon dioxide production (**i**), respiratory exchange ratio (**j**) and heat production (**k**). **(l-o)** Locomotor movements of WT (n=7) and YS (n=7) mice monitored by the CLAMS for horizontal (**l**) and vertical (**m**) axis activities, as well as ambulatory (**n**) and voluntary running wheel activities (**o**). Mice were within the controlled age range (9.3 ± 0.3-week-old) at the time of the study, except for the age-dependent analyses (**a-c**). All mice were housed at controlled room ambient temperature (20.2 ± 0.4°C). P values are indicated as analyzed by F-test for deviation from zero-rate constant of non-linear regression (**a**), analysis of covariance (ANCOVA) for linear regression (**b,c**), Welch's t-tests (**d-g**), two-way analysis of variance with Sidak's multiple comparisons tests (**h-o**). All statistical tests are two-sided. Data are represented as 95% confidence intervals (CI) from the non-linear best-fit curve (**a**), or mean ± standard deviation (**d-o**). The estimated gross energy content of mouse normal chow diet (NCD) used is 4.31 kcal/g based on the supplier's description. Source data are provided as a Source Data file.

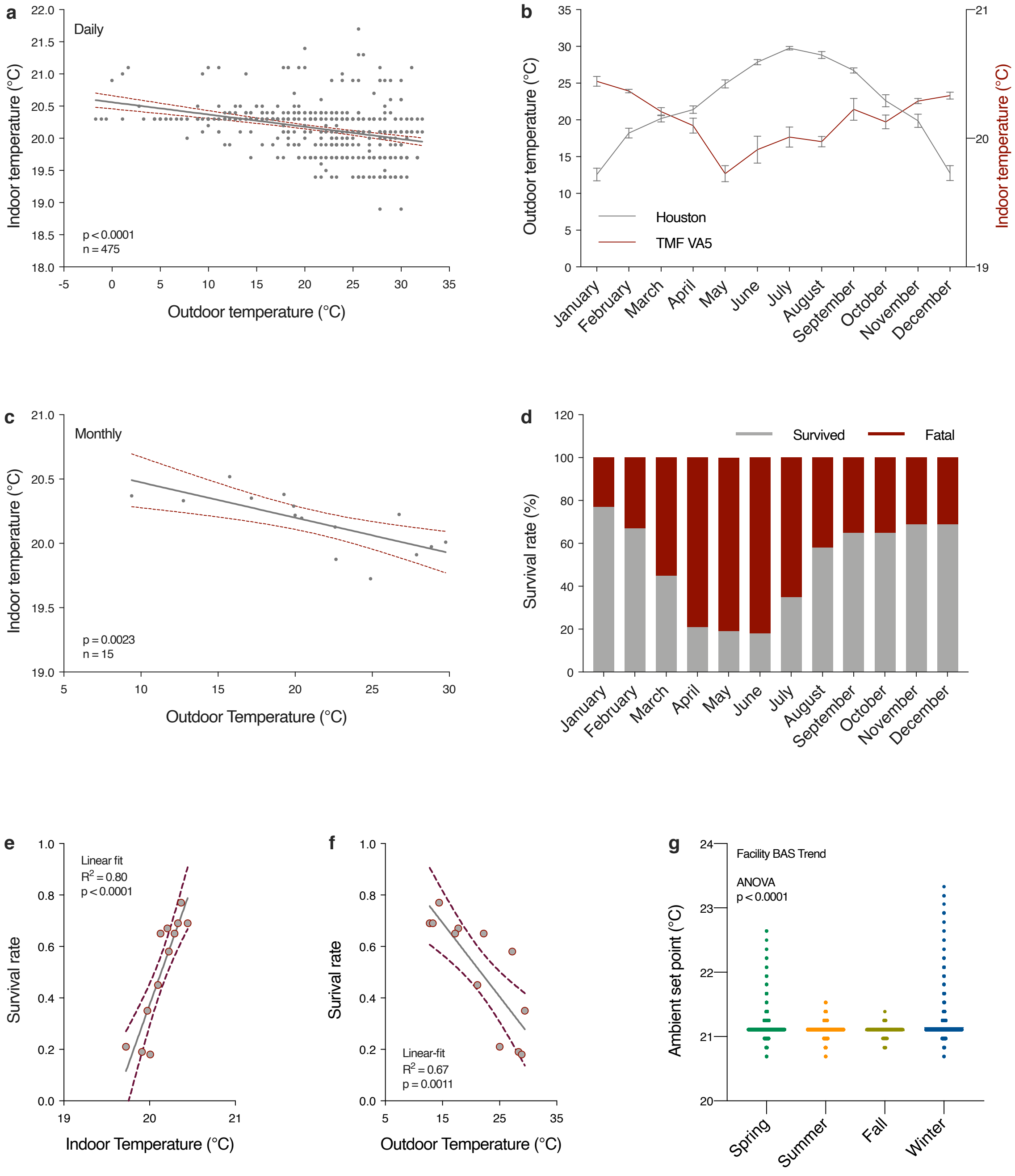

Supplementary Figure 4. Seasonal variations in post-heat exposure survival of Y524S mice. (**a-c**) Seasonal variation and the relationship of outdoor (Houston, Texas) and indoor (transgenic mouse facility, TMF) temperatures analyzed by daily (**a**) and monthly averages (**b,c**). (**d**) Seasonal variation of survival rate of YS mice after the acute 37°C acute heat exposure based on monthly averages from historical data between 2012 and 2019, with the majority of data collected from 2016 to 2018. (**e,f**) Relationship of average indoor (**e**) and outdoor (**f**) ambient temperature and survival rate of YS mice after the 37°C heat challenge in each month of the year. (**g**) Seasonal variation of the animal housing facility ambient temperature trend data determined by the building automation system (BAS). All mice were housed at controlled room ambient temperature (20.2 ± 0.4°C). Mice were within the controlled age range (9.3 ± 0.3-week-old) at the time of the study. P values are indicated as analyzed by F-test for deviation of zero-slope of linear regression (**a,c,e,f**). All statistical tests are two-sided. Data are represented as 95% confidence intervals (CI) from the linear best-fit line (**a,c,e,f**), or mean ± standard deviation (**b**). The scale for indoor ambient temperature is enlarged to show subtle but significant seasonal variation (**b**). The data for outdoor temperature in Houston was obtained from the National Weather Service of the national oceanic and atmospheric administration (NOAA) at *www.weather.gov* for Houston, Texas, United States (Latitude: 29.64°N, Longitude: 95.28°W, Elevation: 14.02 m at KHOU). Source data are provided as a Source Data file.

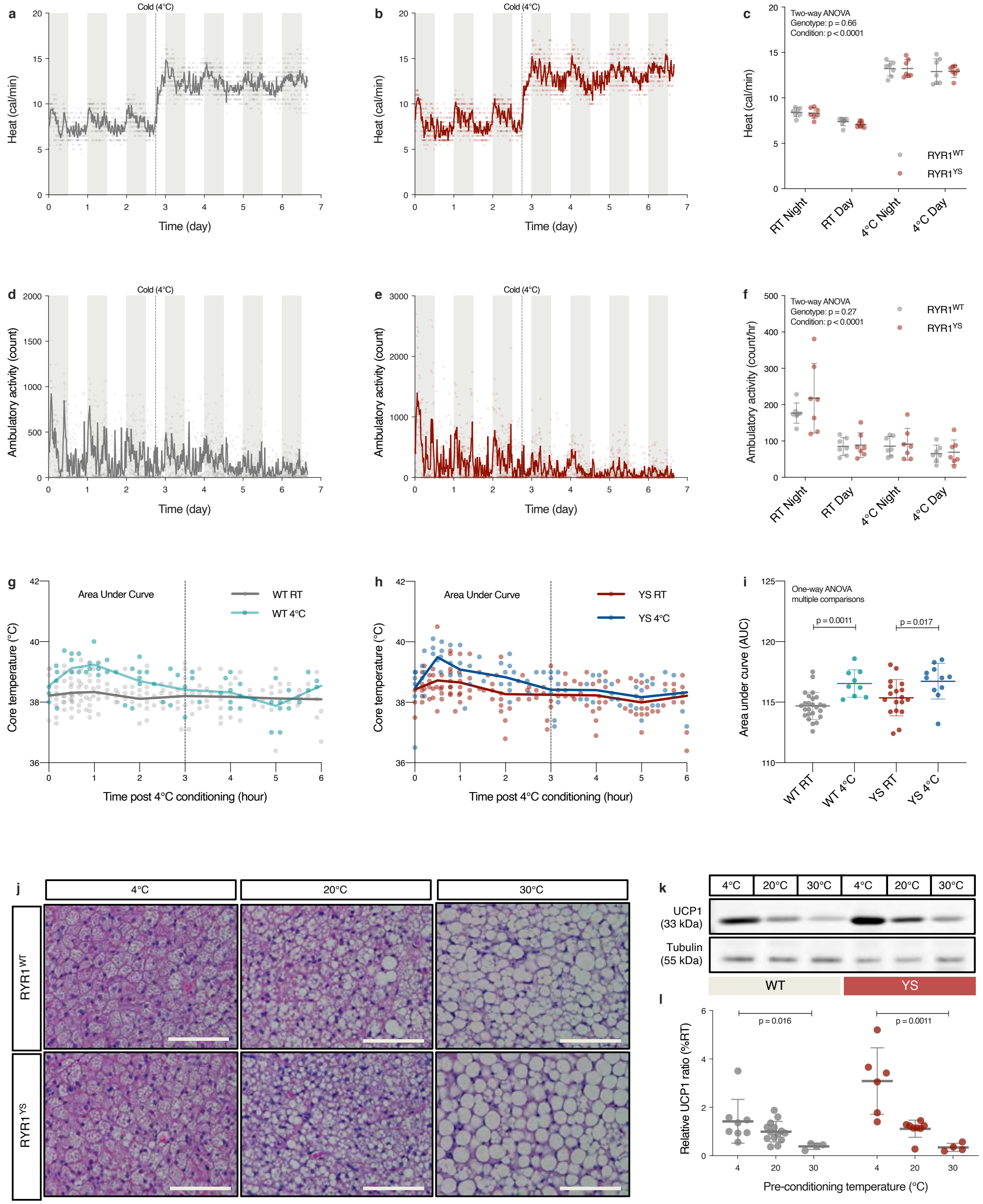

Supplementary Figure 5. Brown adipose tissue mediates cold-induced adaptive thermogenesis in mice. (**a-c**) Heat production of WT (n=7, **a**) and YS (n=7, **b**) mice at room temperature or 4ºC (**c**). (**d-f**) Ambulatory activity of WT (n=7, **d**) and YS (n=7, **e**) mice at room temperature or 4ºC (**f**). (**g-i**) Core body temperature of WT (**g**) and YS (**h**) mice *after* 1 week of preconditioning at 4°C monitored over time compared to room temperature controls (**i**). (**j**) Hematoxylin and eosin (H&E) stain images of interscapular brown adipose tissue (iBAT) from WT and YS littermates preconditioned chronically for 1 week at 4°C, room temperature, and 30°C ambient temperatures. (**k,l**) Representative immunoblots (**k**) and relative protein levels (**l**) for mitochondrial uncoupling protein (UCP1) in interscapular brown adipose tissue of WT and YS littermates preconditioned for 1 week at 4°C (WT n=8, YS n=6), room temperature (WT n=11, YS n=4), and 30°C (WT n=4, YS n=4) ambient temperature. Mice were within the controlled age range (9.3 ± 0.3-week-old) at the time of the study. P values are indicated as analyzed by two-way analysis of variance (ANOVA) with Sidak's multiple comparisons tests (**c,f**), one-way ANOVA with Sidak's multiple comparison tests (**i**) and one-way ANOVA with post-test for linear trend (**l**). All statistical tests are two-sided. Data are represented as mean ± standard deviation (**c,f,i,l**). Shaded areas indicates periods of darkness in the light-dark cycles (**a,b,d,e**). Scale bars are 100 µm for histological images (**j**). Source data are provided as a Source Data file.

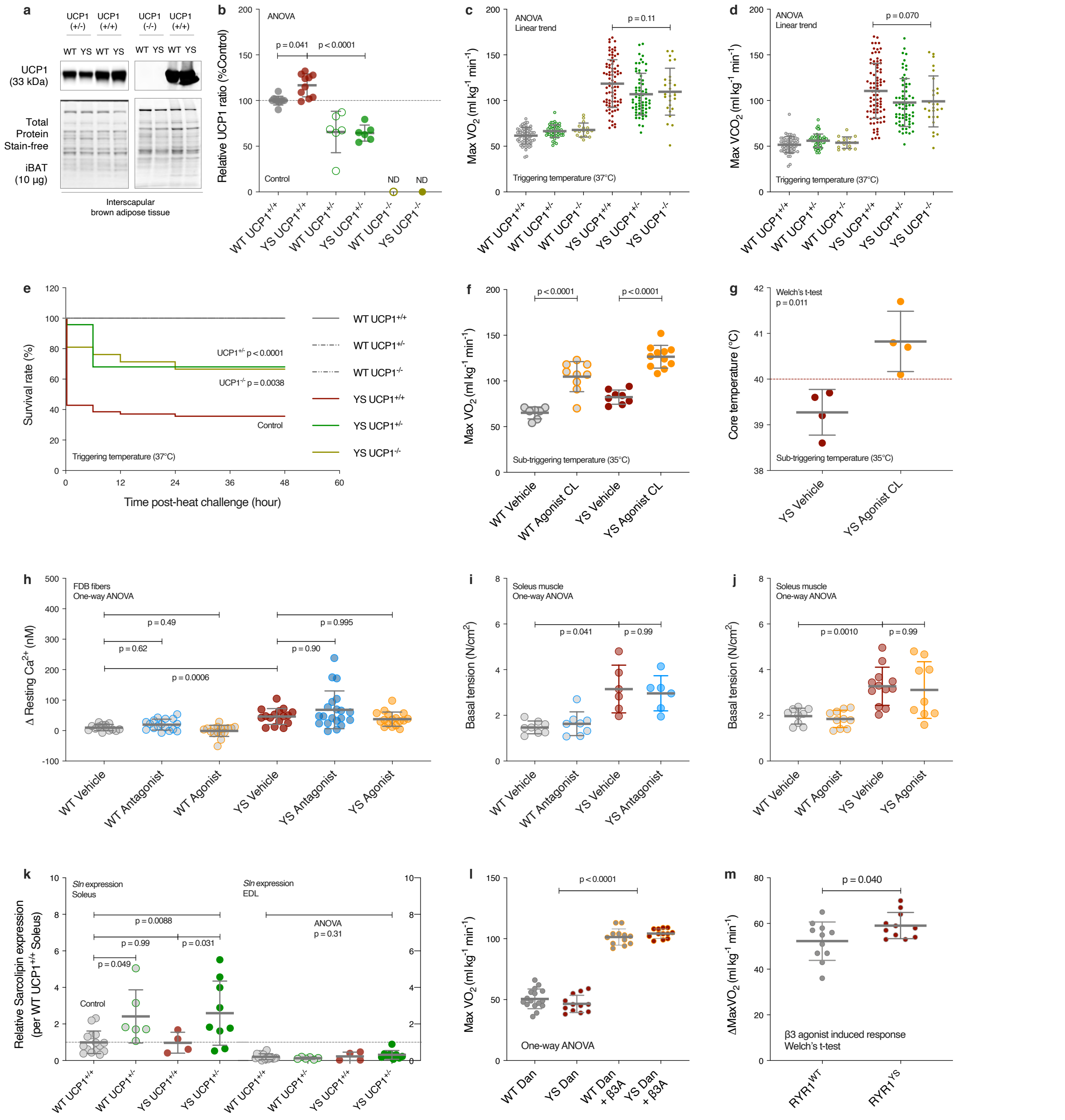

Supplementary Figure 6. Pharmacological and genetic modulation of brown fat on heat sensitivity of Y524S mice. (**a,b**) Representative immunoblots (**a**) and relative levels (**b**) for the mitochondrial uncoupling protein (UCP1) in the interscapular brown adipose tissues (iBAT) of WT and YS mice with wildtype-UCP1 (n=10 each) and heterozygous-UCP1 (n=6 each), using UCP1-ablated (UCP1$^{-/-}$) iBAT as negative controls. **(c,d)** Maximal O_2 consumption (MaxVO_2, **c**) and CO_2 production (MaxVCO_2, **d**) rate of WT (n = 124) and YS (n = 173) mice with or without homozygous genetic ablation of the mitochondrial uncoupling protein (WT/UCP1$^{-/-}$ n = 16, YS/UCP1$^{-/-}$ n = 27) after acute 37°C acute heat exposure. (**e**) Kaplan-Meier analysis of the survival rate of WT and YS mice with or without UCP1 homozygous genetic ablation (WT/UCP1$^{-/-}$ n = 16, YS/UCP1$^{-/-}$ n = 27) after acute environmental heat challenge. Data from mice with heterozygous UCP1 ablation (UCP1$^{+/-}$) are included in determining the gene dosage effect of UCP1 on heat response of the mice (**c-e**). (**f**) MaxVO_2 of WT (n=7 vehicle, n=9 CL) and YS (n=9 vehicle, n=11 CL) mice pre-treated with β3AR agonist (CL316243, 1mg/kg) or vehicle control during acute environmental heat challenge at 35°C. One data point (YS vehicle MaxVO_2 = 161 ml/kg/min) identified as a statistical outlier (more than 2.57 σ above the group mean) was excluded from the analysis based on Grubb's method. (**g**) Core body temperatures of WT (n = 4) and YS (n = 4) mice pre-treated with β3AR agonist or vehicle control after an acute heat challenge. (**h**) Effects of β3AR antagonist or agonist on temperature-dependent increases in resting Ca^{2+} in flexor digitorum brevis (FDB) myofibers from WT (n=14 each) and YS mice (n=16 each). (**i, j**) Effects of β3AR antagonist (**i,** WT n=8 pairs, YS n=6 pairs) or agonist (**j,** WT n=9 pairs, YS n=9 pairs) on temperature-dependent increases in basal tension of soleus muscle from mice. (**k**) Gene expression of sarcolipin (*Sln*) in soleus and EDL muscle of WT (n=15 wildtype-UCP1, n=6 heterozygous-UCP1) and YS (n=4 wildtype-UCP1, n=9 heterozygous-UCP1) mice with or without UCP1 ablation. (**l**) MaxVO_2 of dantrolene pretreated WT (n=11) and YS (n=11) mice before and after β3AR agonist treatment. (**m**) Differential β3AR agonist-induced hypermetabolic response (ΔMaxVO_2) between WT (n=11) and YS (n=11) mice. All mice were within the controlled age range (8.8 ± 1.0-week-old) at the time of the study. All mice were housed at controlled room ambient temperature (20.2 ± 0.4°C). P values are indicated as analyzed by ordinary one-way analysis of variance (ANOVA) with Sidak's multiple comparisons test (**b,i,j**), one-way ANOVA with post-test for linear trend (**c,d**), Mantel-cox log-rank test (**e**), one-way ANOVA with Tukey's multiple comparisons test (**f,l**), Welch's t-test (**g,m**), one-way ANOVA with Dunnett's multiple comparisons test (**h,k**). All statistical tests are two-sided. Data are represented as mean ± standard deviation (**b-d,f-m**). Source data are provided as a Source Data file.

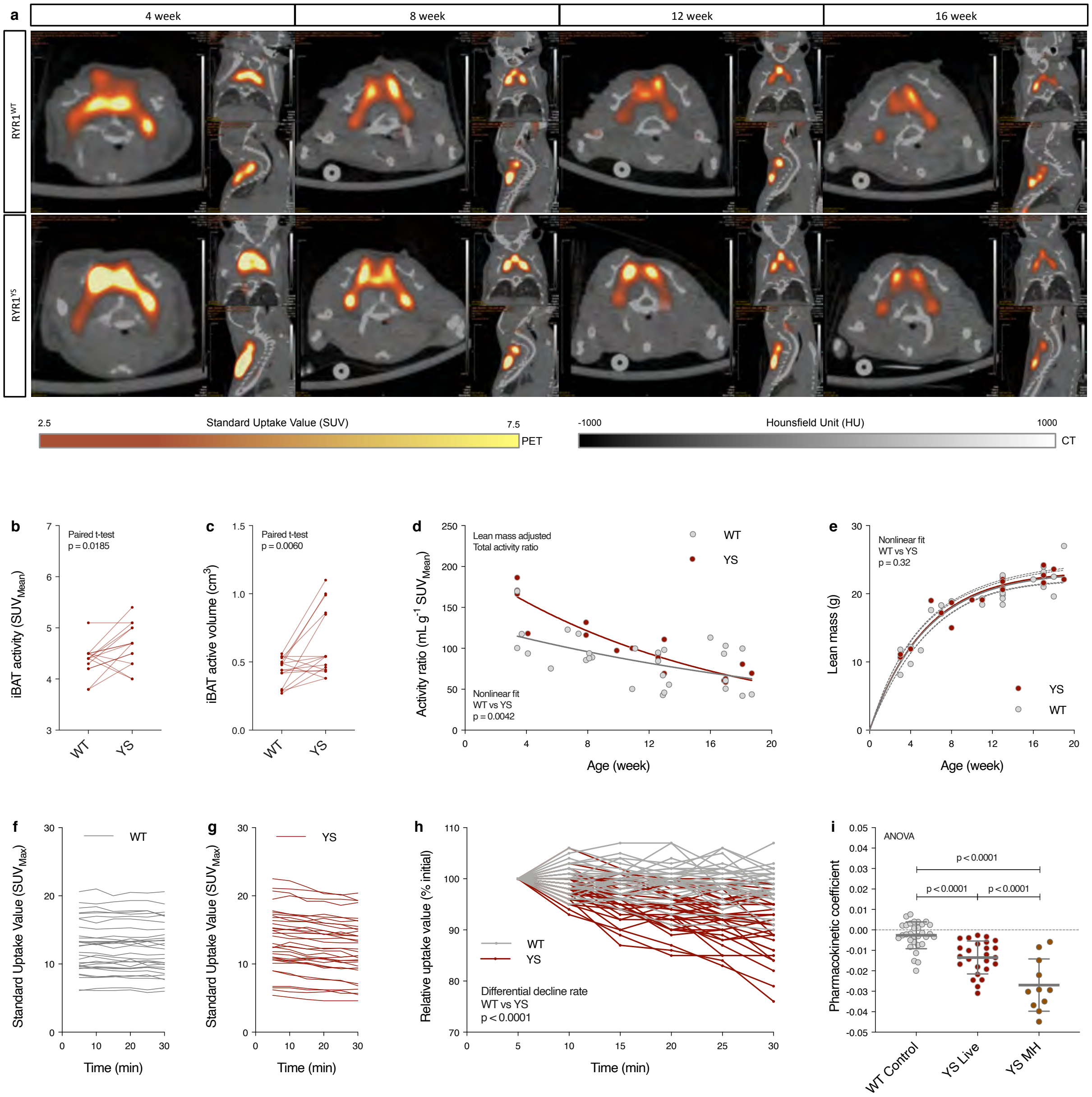

Supplementary Figure 7. Micro-PET/CT functional imaging in Y524S mice. (**a**) Representative positron-emission tomography and computed tomography (PET/CT) scans from the interscapular regions of WT and YS littermate in axial, coronal and sagittal views from various age groups. (**b,c**) Comparison of iBAT mean activities (**b**) and active volumes (**c**) of WT and YS mice in littermate pairs (n=15). (**d**) Age-related decline of lean body mass adjusted total interscapular brown adipose tissue (iBAT) activity in WT (n=28) and YS (n=17) mice. (**e**) Lean mass of WT (n=28) and YS (n=17) mice analyzed for PET/CT. (**f-h**) Pharmacokinetic analysis of ^{18}F-fluorodeoxyglucose (FDG) uptake activity during the 30 minutes of PET/CT imaging in WT (n = 31, **f**) and YS (n = 36, **g**) mice for standard uptake value (SUV) and normalized uptake value to initial (%initial, **h**). (**i**) Pharmacokinetic coefficient of FDG uptake in iBAT for each animal as grouped by malignant hyperthermia (MH) status following isoflurane exposure during imaging (WT control n = 31, YS live n = 25, YS MH n = 11). All mice were housed at controlled room ambient temperature (20.2 ± 0.4°C). P values are indicated as analyzed by paired t-test (**b,c**), F-test for the differential rate constant of non-linear regression (**d,e**), F-test for the differential slope of linear regression (**h**), and ordinary one-way analysis of variance (ANOVA) with Tukey's multiple comparisons test (**i**). All statistical tests are two-sided. Data are represented as 95% confidence intervals (CI) from the non-linear best-fit curve (**e**), or mean ± standard deviation (**i**). Display ranges of standard uptake value (SUV) for and Hounsfield unit (HU) for CT are as indicated (**a**). The scale of PET/CT scans for pups at four weeks of age are enlarged to facilitate visual comparison with adult mice for iBAT size as a proportion to body size. Original scales of PET/CT scans are embedded in each image. Source data are provided as a Source Data file.

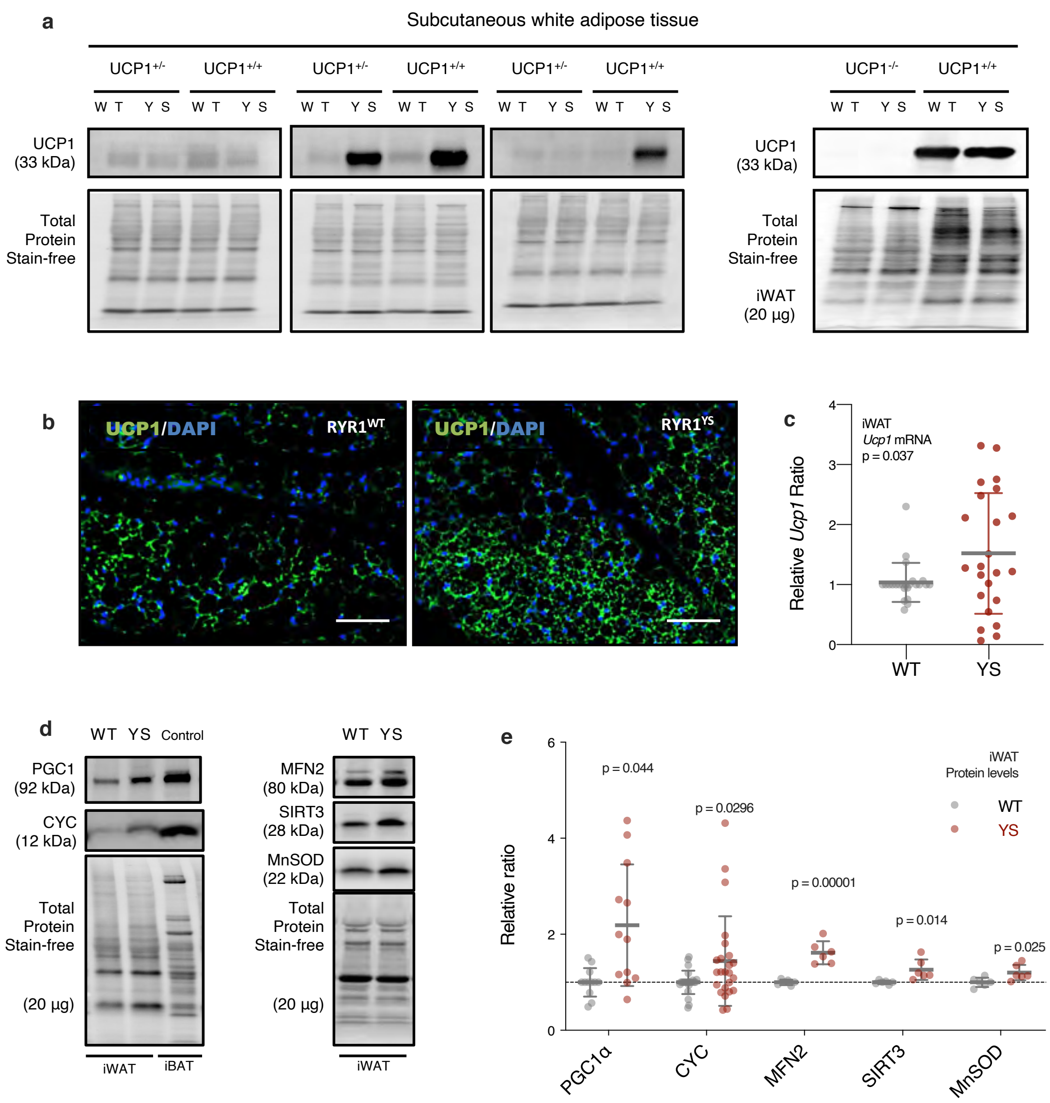

Supplementary Figure 8. Enhanced browning of subcutaneous inguinal white adipose tissue (iWAT) in Y524S mice. (**a**) Representative immunoblots for the mitochondrial uncoupling protein (UCP1) in the inguinal white adipose tissue (iWAT) of WT and YS mice, with UCP1-ablated (UCP1$^{-/-}$) iBAT as negative controls. (**b**) Representative immunofluorescence staining of UCP1 in iWAT of WT and YS littermate. (**c**) Relative expression of *Ucp1* mRNA in iWAT of WT (n=24) and YS (n=23) littermate. (**d,e**) Representative immunoblots (**d**) and relative protein levels (**e**) of adipose tissue browning markers in iWAT, including proliferator-activated receptor γ coactivator-1α (PGC1α, WT n=12, YS n=12), cytochrome C (CYC, WT n=24, YS n=24), mitofusin-2 (MFN2, WT n=6, YS n=6), sirtuin-3 (SIRT3, WT n=6, YS n=6), and manganese superoxide dismutase (MnSOD, WT n=6, YS n=6). Mice were within the controlled age range (10.0 ± 1.8-week-old) at the time of the study. All mice were housed at controlled room ambient temperature (20.2 ± 0.4°C). P values are indicated as analyzed by Welch's t-test (**c,e**). All statistical tests are two-sided. Data are represented as mean ± standard deviation (**c,e**). Scale bars are 100 µm from the histological images of iWAT (**b**). Cell nuclei are labeled with 4',6-diamidino-2-phenylindole (DAPI) fluorescence staining (**b**). Source data are provided as a Source Data file.

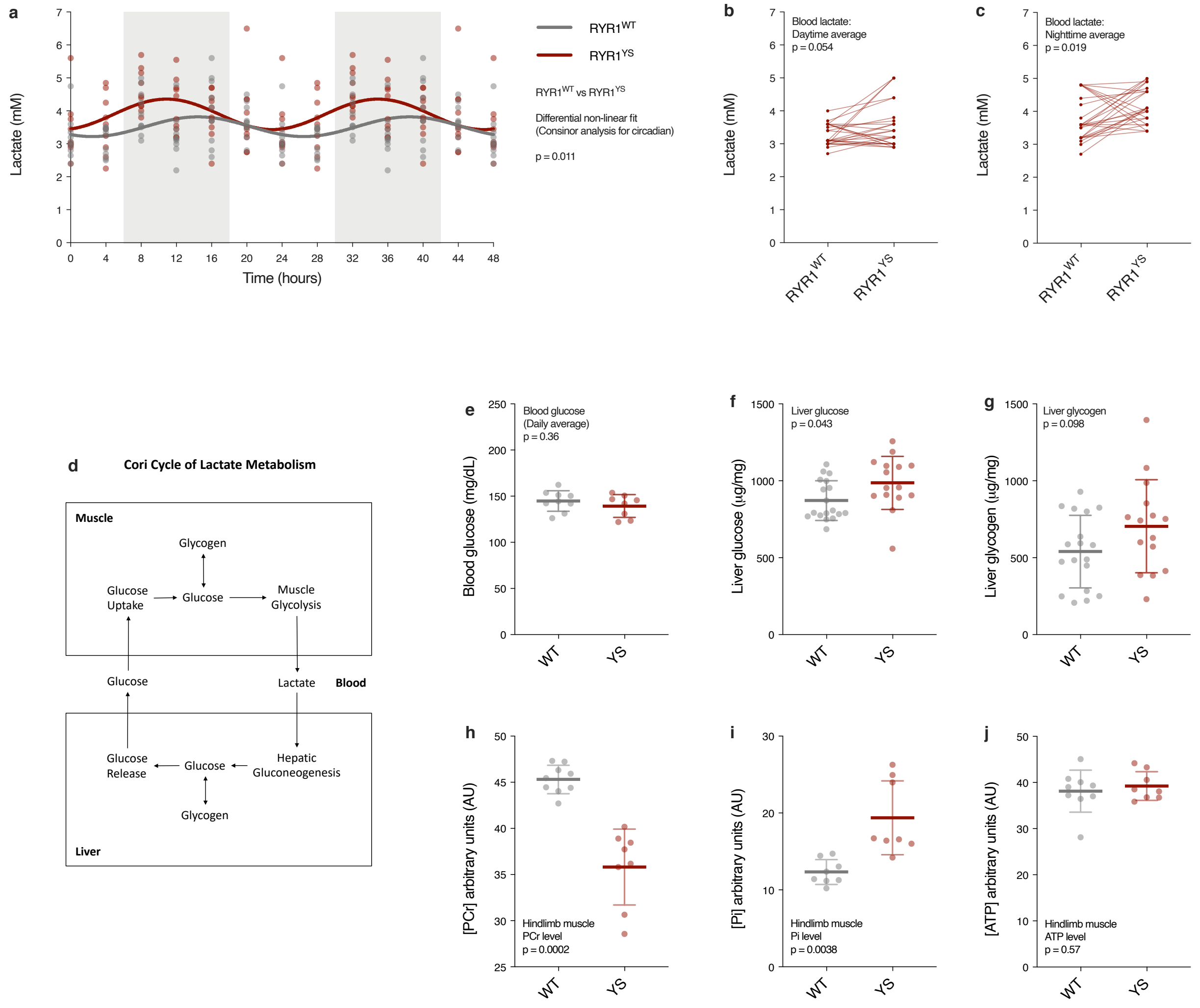

Supplementary Figure 9. Lactate metabolism in Cori Cycle and increased muscle metabolic activities. (**a**) Circadian pattern of circulating lactate levels of WT (n=10) and YS (n=10) mice measured in tail artery blood. Two-day averages of the same time points are plotted twice to better visualize rhythmicity. (**b,c**) Littermate comparison of tail artery blood lactate concentration between WT and YS mice in day (**b**) and night (**c**). (**d**) Schematic diagram of muscle and liver lactate metabolism in a classical Cori Cycle. (**e**) Blood glucose concentration of WT (n=8) and YS (n=8) littermates. (**f,g**) Glucose (**f**) and glycogen (**g**) concentration as measured in liver isolated from WT (n=18) and YS (n=15) littermates. (**h-j**) Quantification of phosphocreatine (PCr, **h**), inorganic phosphate (Pi, **i**) and adenosine triphosphate (ATP, **j**) levels based on analysis of the ^{31}P NMR spectra from WT (n=9) and YS (n=8) hind limb muscles. One data point (WT [Pi] = 21.94 a.u.) identified as a statistical outlier (more than 2.41 σ above the group mean) was excluded from the analysis based on Grubb's method. Mice were within the controlled age range (10.6 ± 1.8-week-old) at the time of the study. All mice were housed at controlled room ambient temperature (20.2 ± 0.4°C). P values are indicated as analyzed by F-test for the differential amplitude and baseline of the COSINOR-based circadian rhythmometry (**a**), paired t-test (**b,c**), Welch's t-test (**e-j**). All statistical tests are two-sided. Data are represented as mean ± standard deviation (**e-j**). Source data are provided as a Source Data file.

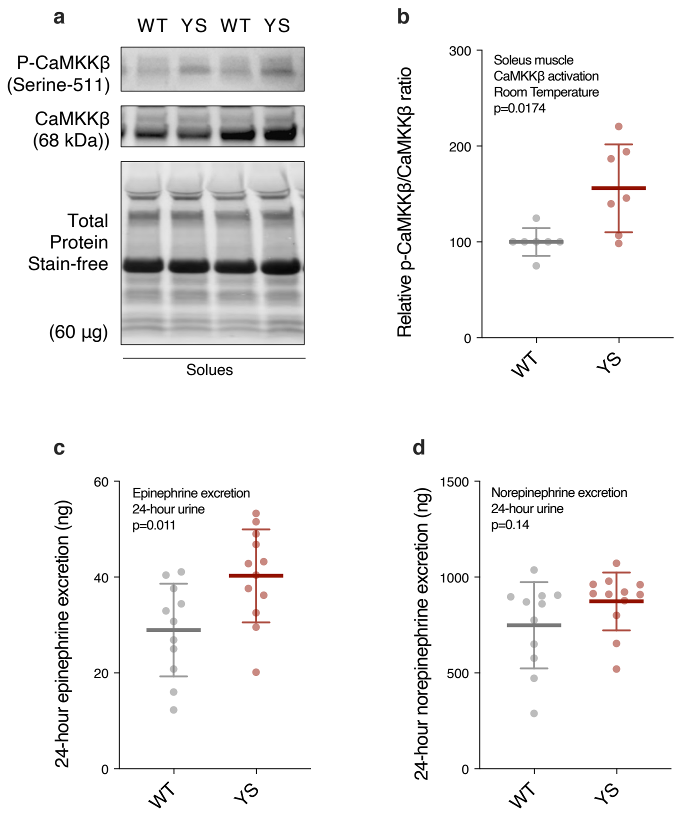

Supplementary Figure 10. Activation of Ca^{2+}/calmodulin kinase kinase (CaMKKβ) and circulating catecholamine levels. (**a,b**) Representative immunoblot (**a**) and relative level of phosphorylation for CaMKKβ in WT (n=7) and YS (n=7) soleus muscle. (**c,d**) Total excretion of epinephrine (**c**) and norepinephrine (**d**) of WT (n=11) and YS (n=12) mice as measured in 24-hour urine. All mice were within the controlled age range (10.0 ± 1.8-week-old) at the time of the study. All mice were housed at controlled room ambient temperature (20.2 ± 0.4°C). P values are indicated as analyzed by Welch's t-test (**b,d**). All statistical tests are two-sided. Data are represented as mean ± standard deviation (**b-d**). Source data are provided as a Source Data file.

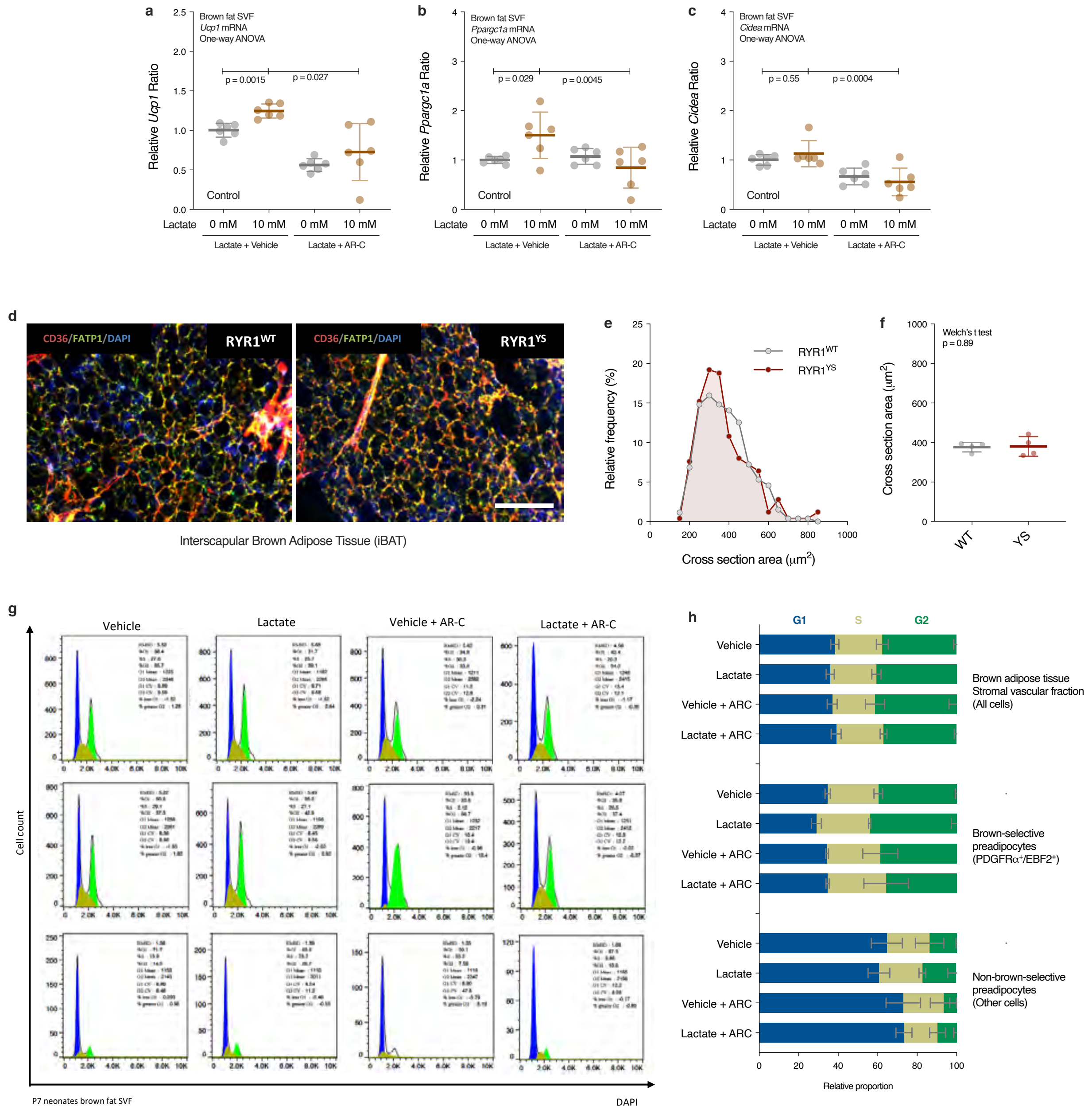

Supplementary Figure 11. Lactate enhances brown adipogenesis in Y524S mice. (**a-c**) Relative expression of brown adipogenic related genes, including *Ucp1* (**a**), *Ppargc1a* (**b**) and *Cidea* (**c**) in differentiated iBAT SVF cells treated with vehicle control (n=6), lactate (n=6), monocarboxylate transporter (MCT1/2) inhibitor (n=6) or both (n=6). (**d**) Representative immunofluorescence staining of adipocytes membrane markers, including fatty acid translocase cluster of differentiation (CD36) and fatty acid transport protein-1 (FATP1), as well as 4',6-diamidino-2-phenylindole (DAPI) cell nuclei staining in iBAT of WT and YS littermates. (**e,f**) Distribution (**e**) and quantification (**f**) of adipocytes cross sectional area in iBAT of WT (n=4) and YS (n=4) littermates. (**g**) Representative histograms for cell cycle analysis by DNA content, in proliferating iBAT SVF cells treated with vehicle control, lactate, monocarboxylate transporter (MCT1/2) inhibitor or both. (**h**) Summary analysis for relative proportion of cells in G1, S, and G2 phases in overall iBAT SVF cells (top), brown-selective preadipocytes fraction (middle), and all other cells (bottom). P values are indicated as analyzed by ordinary one-way analysis of variance (ANOVA) with Dunnett's multiple comparisons test (**a-c**), and Welch's t-test (**f**). All statistical tests are two-sided. Data are represented as mean ± standard deviation (**a-c,f**) and mean ± standard error of the mean (h). DNA content of cells are determined by DAPI fluorescent intensity quantitatively. Scale bar is 100 μm from the histological images (**d**). Source data are provided as a Source Data file.

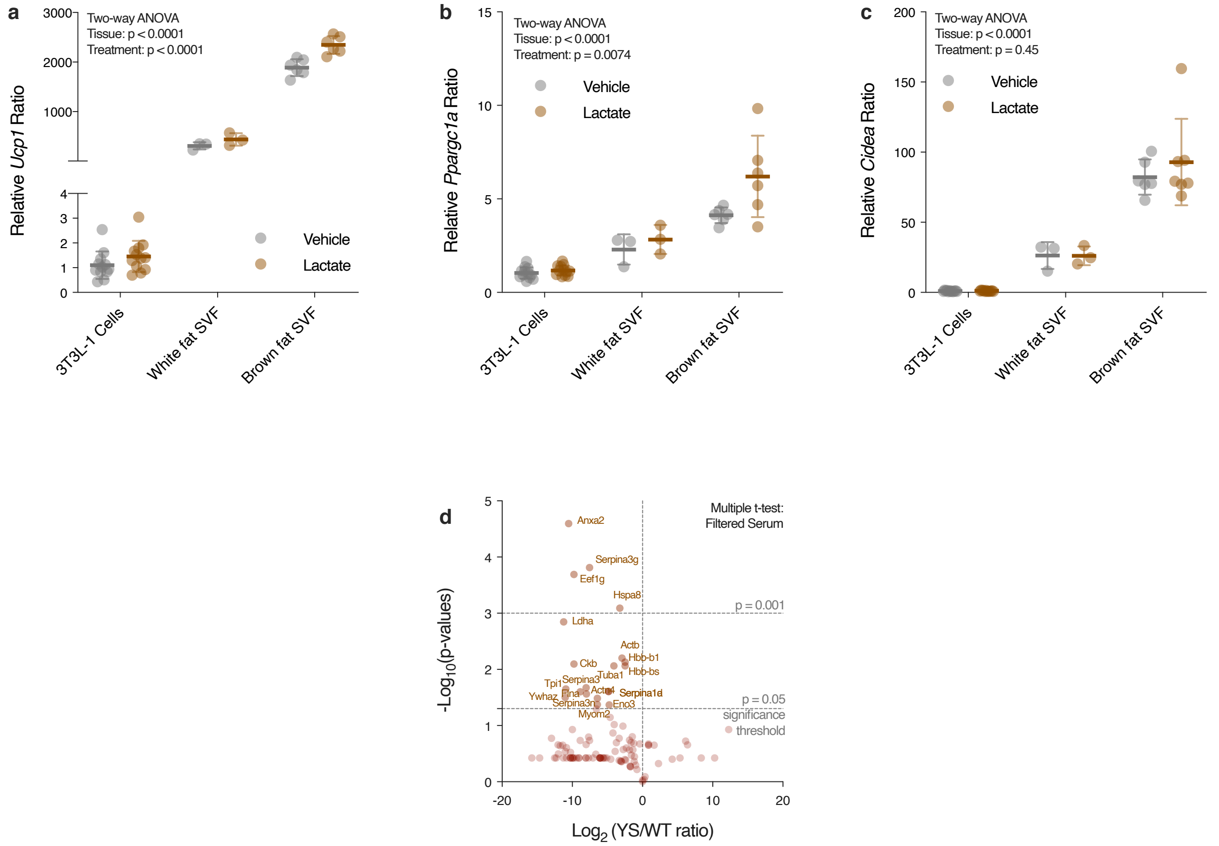

Supplementary Figure 12. Circulating lactate enhances brown adipogenesis in both brown and white fat. (**a-c**) Relative expression of brown adipogenic related genes, including *Ucp1* (**a**), *Ppargc1a* (**b**) and *Cidea* (**c**) in differentiated 3T3L1 preadipocytes (n=12), iWAT SVF cells (n=3), and iBAT SVF cells (n=6) treated with vehicle control or lactate. (**d**) Volcano plot for the level of proteomic changes in filtered serum samples of YS (n=3) mice relative to the WT (n=3) control littermates, of which were used in the preadipocytes differentiation experiments. P values are indicated as analyzed by two-way analysis of variance (ANOVA) (**a-c**), and two-sided unpaired t-test without adjustment for multiple comparisons (**d**). All statistical tests are two-sided. Data are represented as mean ± standard deviation (**a-c**). Source data are provided as a Source Data file.

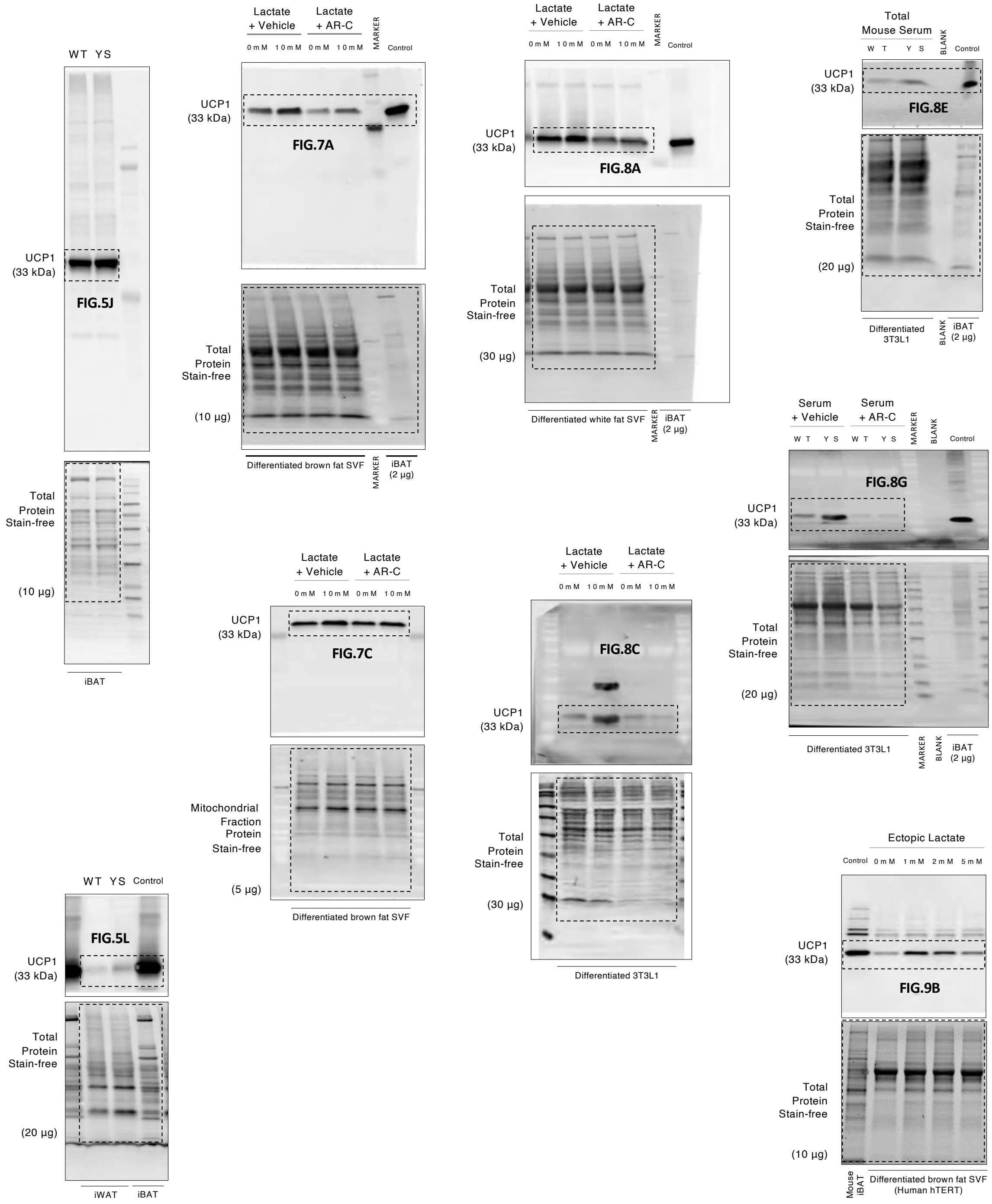

Supplementary Figure 13. Uncropped version of the immunoblots presented in the main figures. Antibody target and identifying information were indicated for each panel. Anti-UCP1 antibody for mouse were used for UCP1 detection from mouse samples in **Figure 5, Figure 7** and **Figure 8**. Anti-UCP1 antibody for mouse/human were used for UCP1 detection from human samples in **Figure 9**.

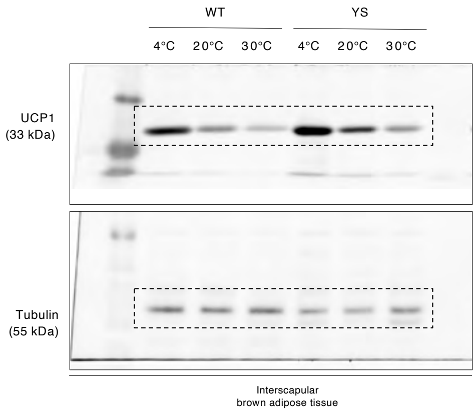

SUPPLEMENTARY FIG.5K

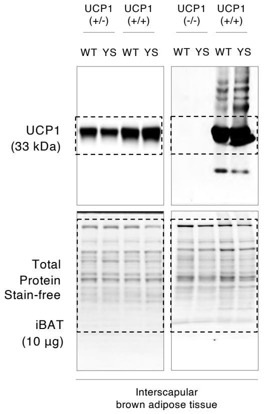

SUPPLEMENTARY FIG.6A

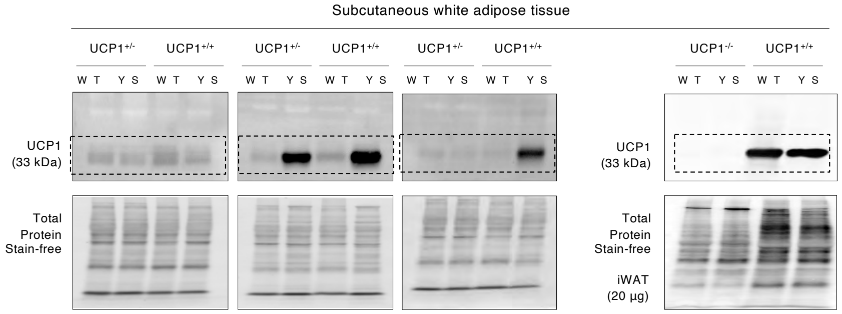

SUPPLEMENTARY FIG.8A

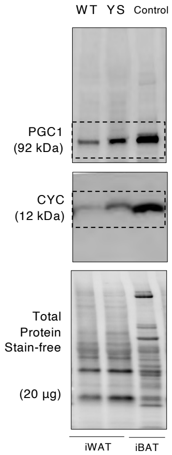

SUPPLEMENTARY FIG.8D (Left)

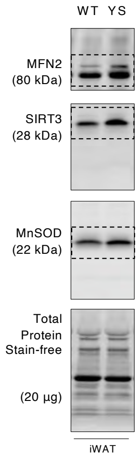

SUPPLEMENTARY FIG.8D (Right)

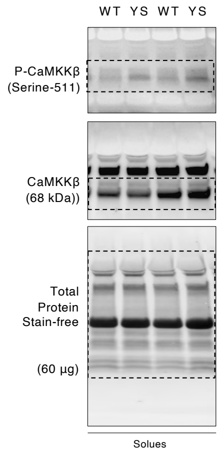

SUPPLEMENTARY FIG.10A

Supplementary Figure 14. Uncropped version of the immunoblots presented in the supplementary figures. Antibody target and identifying information were indicated for each panel. Anti-UCP1 antibody for mouse were used for UCP1 detection from mouse samples in **Supplementary Figure 5, Supplementary Figure 6, and Supplementary Figure 8.**

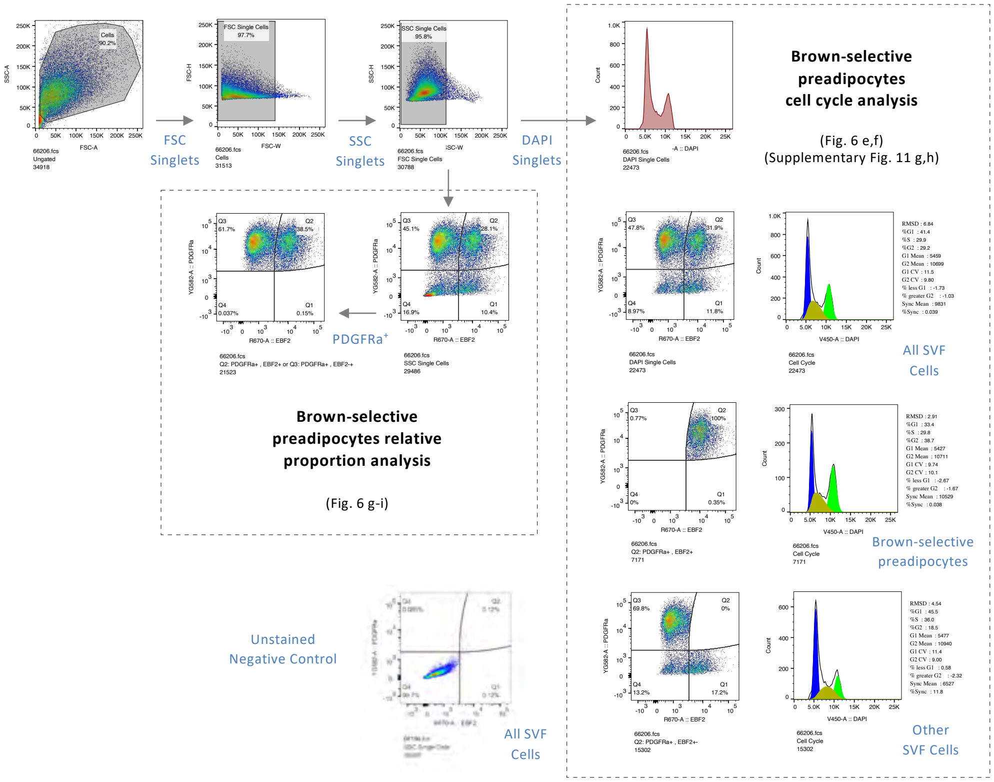

Supplementary Figure 15. Gating strategy for flow cytometry analysis. Forward scatter (FCS) and side scatter (SSC) gates of starting cell population, gates for singlets screening, and threshold for positive and negative populations are indicated. Identical gating threshold was applied to all samples within the experiment. Cell cycle analysis was performed based on the quantitative DAPI intensity for DNA content by the default univariate cell cycle model without constrains.

Supplementary Table 1 | Antibodies

Antibody	Supplier	WB Dilution	IF Dilution	FC Dilution	Catalogue No.
UCP1 (Mouse)	Abcam	1:1000	1:400	NA	AB10983
UCP1 (Human/Mouse)	Invitrogen	1:1000	NA	NA	PA1-24894
EBF2	Bioss	NA	NA	1:50	BS-11740R-A647
VDAC	Cell Signaling	1:1000	NA	NA	4866S
SIRT3	Cell Signaling	1:1000	NA	NA	5490S
MFN2	Cell Signaling	1:1000	NA	NA	9482S
CYC	Cell Signaling	1:1000	NA	NA	4280
Tubulin	DSHB	1:500	NA	NA	6G7
CD140a	eBioscience	NA	NA	1:400	12-1401-81
PGC1a	NOVUS Biologicals	1:1000	NA	NA	NBP1-04676
CD36	NOVUS Biologicals	NA	1:200	NA	NB110-59724
MnSOD	Santa Cruz	1:500	NA	NA	SC-133254
CaMKK-beta	GeneTex	1:1000	NA	NA	GTX108305
p-CaMKK-beta (Ser511)	Cell Signaling	1:1000	NA	NA	12818S
FATP1	Santa Cruz	NA	1:100	NA	SC-25541

WB, Western blotting
IF, immunofluorescence staining
FC, Flow cytometry

Abcam (Cambridge, MA, United States)
Invitrogen (Carlsbad, CA, United States)
Bioss (Woburn, MA, United States)
Cell Siganling (Danvers, MA, United States)
DSHB (Iowa City, IA, United States)
eBioscience (San Diego, CA, United States)
NOVUS Biologicals (Littleton, CO, United States)
BD Biosciences (San Jose, CA, United States)
Santa Cruz Biotechnology (Dallas, TX, United States)
GeneTex (Irvine, CA, United States)

Supplementary Note 1

Heat-sensitivity in patients with malignant hyperthermia susceptibility (MHS): A retrospective cohort study and systematic review

Hui J. Wang[1,2], Sheila Riazi[3], Susan L. Hamilton[1,2]

1. Department of Molecular Physiology and Biophysics, Baylor College of Medicine
2. Translational Biology and Molecular Medicine Graduate Program, Baylor College of Medicine
3. Department of Anesthesiology, University of Toronto

Abstract Summary

Background. Pathogenic variants in the skeletal muscle calcium release channel (RYR1) underlie both malignant hyperthermia susceptibility (MHS) to triggering pharmacological agents and the MHS-associated life-threatening hypermetabolic response to heat. To further assess the characteristics associated with heat-sensitivity in MHS patients, we conducted a retrospective cohort study and systemic review on *RYR1* variants, age, gender, clinical symptoms and survival outcome observed in heat-sensitive MHS patients.

Methods. A retrospective cohort study for patients referred to malignant hyperthermia investigation unit (MHIU) in Toronto during 1994-2019 was performed under the guidelines from STrengthening the Reporting of OBervational studies in Epidemiology (STROBE). Additionally, a systematic review of case reports published during 1980-2019 on heat-sensitivity in patients with MHS was performed under the guidelines from Preferred Reporting Items for Systematic reviews and Meta-Analysis (PRISMA). The data reported from both the systematic review and the retrospective cohort study were combined to complete the statistical analysis.

Results. Heat strokes, myalgia, muscle cramps, muscle rigidity, and rhabdomyolysis are the common reported symptoms associated with sensitivity to environmental or exertional heat. The proportion of heat-sensitive patients observed in male carriers of *RYR1* variants was significantly higher compared to that of the female carriers ($p <$ 0.0001, n = 179, Risk ratio: 2.6, 95% CI: 1.9 to 3.8, Fisher's exact test). The mortality rate after heat-induced episodes in heat-sensitive MHS patients reported in pediatric subgroup was significantly higher compared to that of the adult ($p <$ 0.0001, n = 138, Risk ratio: 14.4, 95% CI 4.6 to 45.4, Fisher's exact test). Additionally, the *RYR1* variants associated with heat-sensitivity were found in RYR1 domains known to harbor MHS mutations.

Conclusion. The retrospective cohort study and systematic review showed a male and pediatric predominance in significant heat-sensitivity among MHS patients with *RYR1* pathogenic variants. Further research, including additional longitudinal clinical studies and controlled preclinical experiments, is needed to confirm this finding and identify interventions for the prevention and management of the life-threatening heat-sensitivity in MHS patients.

Introduction

Malignant hyperthermia susceptibility (MHS) is a life-threatening pharmacogenetic disorder of the skeletal muscle calcium regulation [PMID: 20301325]. Malignant hyperthermia (MH) manifests when susceptible patients are exposed to certain commonly used volatile anesthetics and depolarizing muscle relaxants, characterized by an uncontrolled massive release of calcium stores from the sarcoplasmic reticulum, which leads to sustained skeletal muscle contracture, hypermetabolism, and hyperthermia [PMID: 26238698]. The reported incidence of anesthetic MH episodes is rare, estimated to be 1:10,000 to 1:250,000 anesthetics [PMID: 4014731, PMID: 14501352]. However, the prevalence of MHS is estimated to be as high as 1:2000 to 1:3000 [PMID: 16732084, PMID: 12411788]. Majority of MHS is predisposed by mutations in the skeletal muscle calcium release channel, or the ryanodine receptor type I (RYR1), with *RYR1* pathogenic variants identified in up to 70% to 80% of confirmed MHS patients [PMID: 15731587, PMID: 16835904, PMID: 16917943, PMID: 21455645]. Without prompt treatment with dantrolene, the mortality following an MH episode is extremely high [PMID: 25268394].

MHS mutations in *RYR1* have also been demonstrated to cause enhanced sensitivity to heat-induced sudden death in preclinical studies [PMID: 16284304, PMID: 18394989, PMID: 22231556, PMID: 17122579]. This enhanced heat-sensitivity is characterized as MH-like responses to a short period of environmental or exertional temperature elevation, independent of exposure to triggering pharmacological agents [PMID: 22231556, PMID: 28465322]. Similar to the pharmacologically induced MH response, the MH-like response to heat is potentially life-threatening. In contrast, the heat-induced episodes often occur in non-clinical settings where immediately administration of dantrolene is impractical [PMID: 28430550]. Identifying the risk factors in susceptible individuals is therefore critical, as the management of MHS patients relies primarily on preventive measures, especially for heat-sensitive MHS patients.

Numerous incidences of heat-induced episodes in MHS patients have been observed in clinics, and many of which have been previously published and reviewed in case reports and case series [PMID: 23848295, PMID: 28326467]. To provide an updated summary on the documented cases, we conducted a retrospective cohort study on patients referred to the malignant hyperthermia investigation unit (MHIU) at Toronto General Hospital during 1994-2019 and a systematic review on case reports published between 1980-2019. By assessing the *RYR1* mutations, age, gender, clinical symptoms and survival outcome observed in heat-sensitive MHS patients, we aimed to identify the significant risk factors associated with the life-threatening heat-response in MHS patients with *RYR1* variants.

Methods

Registration

Protocol adapted from PMID: 28326467.

Retrospective Study

Patients referred to the malignant hyperthermia investigation unit (MHIU) at Toronto General Hospital between 1994 and 2019 were included in the retrospective cohort study performed under the STrengthening the Reporting of OBervational studies in Epidemiology (STROBE) guidelines.

Eligibility Criteria. MHS Patients who had significant heat-sensitivity symptoms and carried a variant in *RYR1* were included. The significant heat sensitivity was defined as per patient's report as suffering from heat exhaustion, the occurrence of heatstroke or heat-induced rhabdomyolysis. Family members of patients who carried the same *RYR1* variants were also included.

Variables. Collected data included *RYR1* variants, gender, age at the time of assessment of heat sensitivity, description of symptoms, information on MH reaction if existed, such as clinical grading scale score (CGS), caffeine and halothane contracture results if available, blood lactate levels where available, and previous anesthetic history. The same information from family members, who carried the same *RYR1* variants, was collected.

The institutional research ethics board (REB) at the University of Toronto approved this retrospective study and waived the need for informed consent.

Systematic Review

Clinical case reports published between 1980 and 2019 were systematically reviewed to identify eligible patients, following the preferred reporting items for systemic reviews and meta-analysis (PRISMA) guidelines.

Eligibility Criteria. Clinical case reports and case series with individual patients data on heat-sensitivity in MHS patients were included. Significant heat-sensitivity was defined as suffering from rhabdomyolysis, muscle rigidity, muscle cramps, myalgia, heatstrokes, or other related symptoms after exposure to environmental heat, exertional heat, or fever. As the scope of this systematic review is defined to focus on heat-induced MH-like response, MH episodes due to exposure of triggering volatile anesthetics or depolarizing muscle relaxant within 24 hours were excluded. As the review primarily focuses on MHS patients with *RYR1* variants, patients found with causal MHS variants other than *RYR1*, such as p.Arg1086His and p.Arg174Trp in *CACNA1S*, were excluded. Additionally, bibliographies of case report reviews were also screened to identify additional relevant case report and duplicated individual patients data found in multiple reviews other than the original case reports were removed.

Identifying Studies. Literature published from 1980 to 2019 were searched with Google Scholar search engine with the following medical subject headings (MeSH) or text terms: (1) ryanodine receptor calcium release channel or *RYR1*, (2) heat exhaustion, heat stress disorders, heatstroke, or extreme heat, and (3) death. Relevant clinical case reports and case series were accessed to identify eligible patients via OneSearch at the Texas Medical Center (TMC) library. The core resources available at the TMC library include the following bibliographic databases but not limited to: PubMed, CINAHL Plus, Scopus, EMBASE, ClinicalKey, Psyclnfo, JoVE, VisualDx, AccessMedicine, BrowZine, Cochrane Library, Mediline, RefWorks, Micromedex, Stat!Ref and Nursing Reference Center. Date of the last search: July 15, 2019.

Variables. As an expanded database for heat-sensitivity in patients identified in the MHIU retrospective cohort study, the variables to be collected during the systemic review were chosen based on matching variables collected during the retrospective cohort study. Relevant clinical case reports and case series containing the following information were eligible for inclusion: (1) diagnosis of MHS via *in vitro* muscle contracture testing (IVCT), molecular genetic detection, or both, (2) genetic characterization of *RYR1* sequence variants, including description

of the variants at the genome and protein level and co-segregation of the variants with the disease in the family history when available, (3) description of clinical symptoms related to heat-sensitivity, and (4) description of relevant patient information such as gender, age, and survival outcome when applicable. Age of patients includes both (1) age at the time of initial symptom observation and (2) age at the time of last survival observation. Patients are defined based on the status of MHS diagnosis, regardless of whether or not heat-sensitivity related symptoms were reported. Patients identified as carriers of *RYR1* variants, but reported no heat-related symptoms at the time of symptom observation were regarded as "non-sensitive" or "asymptomatic". Patients identified as carriers of *RYR1* variants, but were not found fatal at the time of last survival observation were regarded as "non-fatal" or "survived".

Data Collection. The above data was manually searched within eligible case reports, collected into the database designated as "heat-sensitivity in patients with *RYR1* variants", and managed under per individual patient bases. Patient-specific information, such as *RYR1* genetics, gender, and time at disease onset or death, were used to distinguish individual patients in the database. Meanwhile, protected health information (PHI) of the individual patient remains de-identified. Studies with aggregated data of the above information but were unable to extract for individual patient data were not included.

Combined Analysis

Risk of Bias. Descriptive individual patients data collected, such as status of MHS diagnosis, characterization of *RYR1* genetics, list of clinical symptoms, and description of most patient characteristics, were included but not subject to statistical analysis. However, other parameters were quantitatively analyzed for statistical significance and subject to baseline imbalance due to reporting bias. Inclusion of patients of various ages, based on reported age of patients at the time of symptom or survival observation, was subject to reporting bias for any particular age groups. To assess the risk of reporting bias for age, the distribution of reported age of all patients was determined for goodness-of-fit to Gaussian distribution, and compared to the expected median age of global population with Wilcoxon signed rank test for any significant discrepancy with the expected age. The expected median age of the global population is defined at 30.2 years old, based on 2013 estimates from the World Health Organization (*www.who.int*). Additionally, the inclusion of patients of either gender was also subject to reporting bias for a particular gender. To assess the risk of reporting bias for gender, the gender ratio of all patients was compared to the expected equal human sex-ratio with binomial test for any significant discrepancy with expected ratio.

Outcomes. The proportion of heat-sensitive patients and their survival rate after heat-episodes, reported between different genders and among various age groups, are compared as pre-specified primary outcomes. Descriptions of heat-sensitive symptoms and characterization of *RYR1* variants reported in heat-sensitive patients are included as secondary outcomes. The percentage of patients with each symptom among all patients was used to describe the prevalence of symptoms. A pathogenicity score of each *RYR1* variant reported in patients was determined to predict the probability of the mutation being benign or damaging, using the HumDiv-trained PolyPhen model.

Statistics. Fisher's exact tests and X^2 tests were used to determine the significance of differential proportions between gender or age groups from the contingency matrix. Relative risk ratio between male and female, or between pediatric and adult patient groups, was generated with Koopman method as the principal measures of effects. The odds ratio between patient groups was generated with Baptista-Pike method as additional descriptive statistics.

Results

Retrospective Cohort Study

Among the malignant hyperthermia susceptible (MHS) patients referred to MH investigation unit (MHIU) between 1994 and 2019, at least 443 of 783 MHS patients (56.6%) complained of having one or more symptoms of heat-sensitivity. In contrast, only 26 out of 1008 MH negative (MHN) patients (2.5%) reported any heat-sensitivity symptoms, and none displayed significantly heat-sensitivity symptoms. In this retrospective cohort study, we focused on 87 MHS patients from 33 families, consisting of 48 patients who had one or more significant heat-sensitivity symptoms as well as their 34 asymptomatic and five mildly symptomatic family members who are carriers of the same *RYR1* variants. Symptoms of heat-sensitivity include profuse sweating, heat cramps, intolerance to hot temperature, heat exhaustion, heat-induced rhabdomyolysis, and heatstroke. Significant heat-sensitivity symptoms were defined as suffering from heat exhaustion, heat-induced rhabdomyolysis, or occurrence of heatstroke. All patients included in the MHIU cohort are provided with complete age, gender, symptom, and survival outcome information. The clinical and genetic information for all patients identified in the MHIU cohort is presented in **Supplementary Table 1**.

Systematic Review

Among the literature published between 1980 and 2019, we identified at least 3050 articles with the pre-specified search terms. In this systematic review, we focused on 38 clinical case reports and case series studies of 116 MHS patients suffering from one or more symptoms of significant heat-sensitivity, defined as suffering from heat exhaustion, heat-induced rhabdomyolysis, or occurrence of heatstroke. Most individual patients data included in the published cohort are provided with complete age, gender, symptom, and survival outcome information. However, complete information on all variables searched was not reported for all patients. We included 86 patients with complete age, symptom, and survival outcome information, and 90 patients with complete gender and symptom information, in the respective age and gender analysis for survival and symptomatic rate. The clinical and genetic information for all patients identified in the published cohort is presented in **Supplementary Table 2**.

Patient Characteristics

We identified a combined total of 203 patients from the retrospective cohort study and systemic review. The demographic characteristics of patients are summarized as distributions for age and gender of all patients (**Figure 1**).

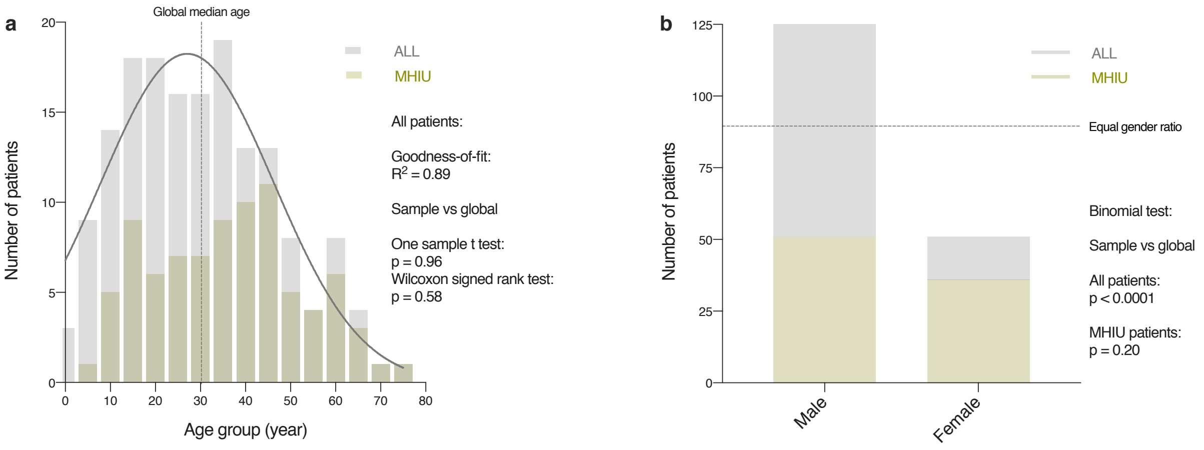

Figure 1. Age and gender distribution of MHS patients reported in MHIU and published cohorts. (**a**) Distribution of age reported in MHS patients of the combined cohort. (**b**) Distribution of gender reported in MHS patients of the combined cohort. R^2 values are indicated to quantify goodness-of-fit to Gaussian distribution. Age of patients refers to the age at admission, or age at the time of initial symptom observation. The

expected median age of the global population is defined at 30.2 years old, based on 2013 estimates from the World Health Organization (*www.who.int*). The expected sex-ratio is defined as an equal ratio of 1:1 human sex-ratio. Statistical test and the number of patients are indicated.

To assess the risk of reporting bias for any particular age groups, we analyzed the reported age of patients at admission, or the age at the initial symptom observation. The distribution of reported age of all patients in the combined cohort was determined for goodness-of-fit to Gaussian distribution, and compared to the expected median age of global population for any significant discrepancy with the expected age. We found that the reported ages of all patients in the combined cohort are normally distributed, and the median age of the patients is comparable to the expected median age of global population. Additionally, the reported age of patients in the MHIU subset is also normally distributed near the global median. This result suggests that the inclusion of patients is randomized for age groups, and is expected to be representative for the median age of the global population. However, we did not determine if the mid-age patients in the combined cohort are overrepresented relative to the global population. To reduce error due to potential bias for the reported incidences in the possibly over-represented mid-age groups, we included both Fisher's exact test and X^2 test to assess proportions of reported incidences in the respective age-dependent contingency analyses.

To assess the risk of reporting bias for gender, we compared the gender ratio of all patients to the expected equal human sex-ratio with binomial test for any significant discrepancy with expected ratio. We found that the reported male-to-female ratio in the combined cohort is significantly higher than the expected equal 1:1 human sex ratio. While the same trend exists, this difference is not significant for the sex ratio in the MHIU subset, which included both symptomatic and asymptomatic MHS patients. We acknowledge this over-representation of males in the combined cohort, and reasoned that this is likely a result of reporting bias for symptomatic patients in the published subset. To reduce error due to the potential bias for reported incidences in the over-represented male groups in the combined cohort, we conducted separate analyses for the MHIU subset, and included both Fisher's exact test and X^2 test to assess proportions of reported incidences in the gender-dependent contingency analyses.

Heat-sensitivity Symptoms

We found that heat strokes, myalgia, muscle cramps, muscle rigidity, and rhabdomyolysis are the common reported symptoms associated with sensitivity to environmental or exertional heat among MHS patients. Other heat-related symptoms, including idiopathic fever, extreme sweating, and muscle weakness, have also been reported. The heat-sensitivity symptoms frequently reported among MHS patients in the combined cohort are summarized as proportions for each condition in male and female patients (**Figure 2**).

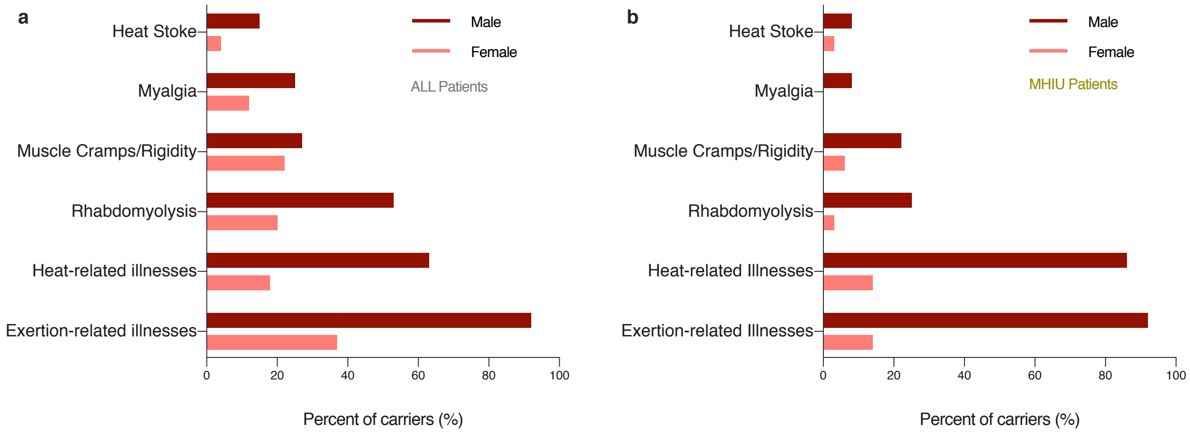

Figure 2. Significant heat-sensitivity related symptoms reported among MHS patients in the combined cohort and MHIU subset. (**a**) Heat-sensitivity related symptoms reported among MHS patients in the combined cohort (n = 179). (**b**) Heat-sensitivity related symptoms reported among MHS patients in the MHIU cohort (n = 87). The percentage of patients with each symptom among all patients in each gender was used to describe the gender prevalence of the symptom.

It has been previously reported that MH episodes, due to exposures to triggering volatile anesthetics or depolarizing muscle relaxants, occur more frequently in male MHS patients than females [PMID: 19104175, PMID: 24974921].

We found a similar significant male predominance for the reported incidence of heat-sensitivity among MHS patients in both the combined cohort and the MHIU subset (**Figure 3**).

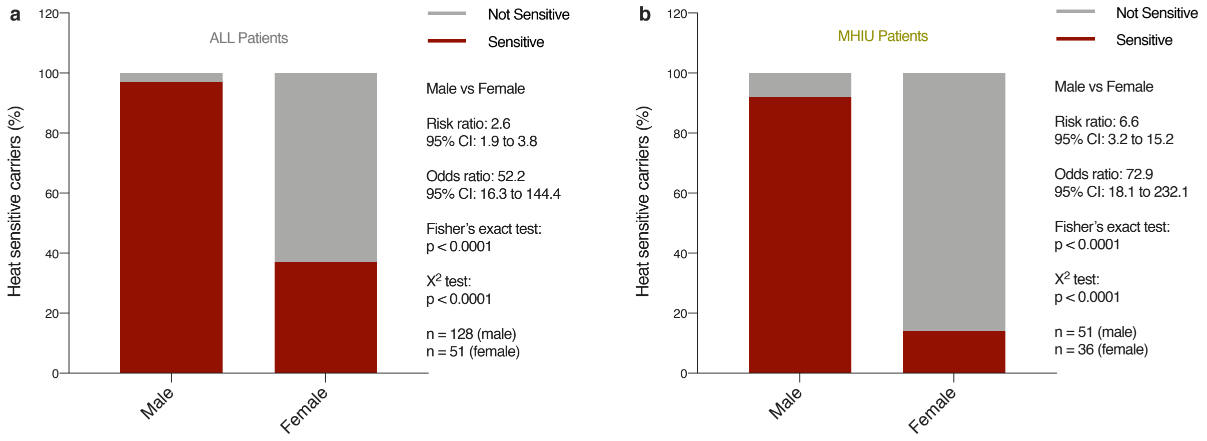

Figure 3. Gender distribution of heat-sensitivity reported among MHS patients in the combined cohort and MHIU subset. (**a**) Gender distribution of patients reported as heat-sensitive among MHS patients in the combined cohort. (**b**) Distribution of gender of patients reported as heat-sensitive among MHS patients in the MHIU subset. Risk ratio, odds ratio, statistical test, and the number of patients are indicated.

Based on the combined cohort, we estimated that the risk of having heat-sensitivity symptoms is about 2.6 times higher for male compared with female among MHS patients. To avoid reporting bias for the symptomatic patients in the published subset, we conducted a separate analysis for the MHIU subset, which included both symptomatic patients and asymptomatic family members carrying the same *RYR1* variants. In the MHIU subset, heat-sensitivity symptoms were reported in over 90% male MHS patients from 33 unrelated families. In contrast, less than 15% of the female family members carrying the same *RYR1* variants reported as heat-sensitive. We estimated that the risk of having heat-sensitivity symptoms is about 6.6 times higher for male compared with female MHS patients from the MHIU subset. As a conservative estimate, we conclude that the risk ratio of at least 1.9 between male and female MHS patients, based on the 95% confidence interval of the estimate from the combined cohort.

Survival Rate

In the retrospective MHIU cohort, we found that many MHS patients had symptoms of significant heat-sensitivity at a younger age, which was changed to milder symptoms such as muscle cramps as they grew older. To further describe the age-related severity of heat-sensitivity, we compared the age distribution of survival rates in MHS patients suffered from heat episodes to the age distribution of which published for patients suffered from MH episodes due to exposure to triggering volatile anesthetics or depolarizing muscle relaxant (**Figure 4**).

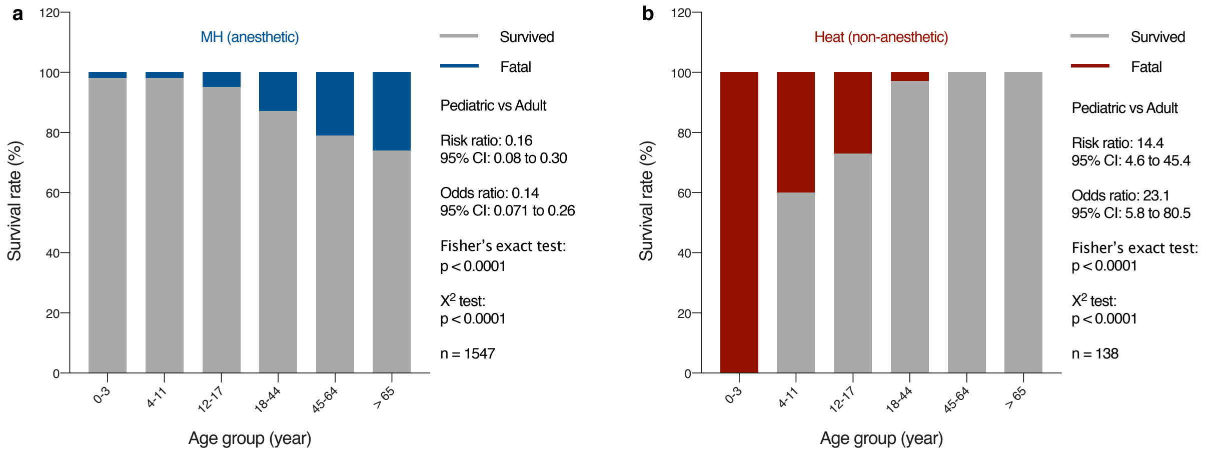

Figure 4. Age distribution of survival rate in MHS patients after MH episodes and heat episodes. (**a**) Age distribution of survival rate in MHS patients after MH episodes as reported by Salazar et al [PMID: 24974921]. (b) Age distribution of survival rate in MHS patients after heat episodes as reported in this study. Risk ratio, odds ratio, statistical test, and the number of patients are indicated.

As reported by Salazar et al., [PMID: 24974921] mortality rate following MH episodes is the highest in elderly MHS patients who are 65 of age and elder. Based on the reported mortality rate of 2.9% in pediatric subgroups (< 17 years of age) and 18.2% in adult, we estimated that the risk ratio between pediatric and adult MHS patients following MH episodes is 0.16 or lower. In contrast, we found a significant predominance in pediatric mortality rate following heat episodes based on analysis from the combined cohort. In particular, patients in the infant and toddler subgroups (0 to 3 years of age) were found to be the most vulnerable to heat. Additionally, the significant pediatric predominance for the reported incidence of heat-sensitivity among MHS patients was found similar between combined cohort and the MHIU subset (**Figure 5**).

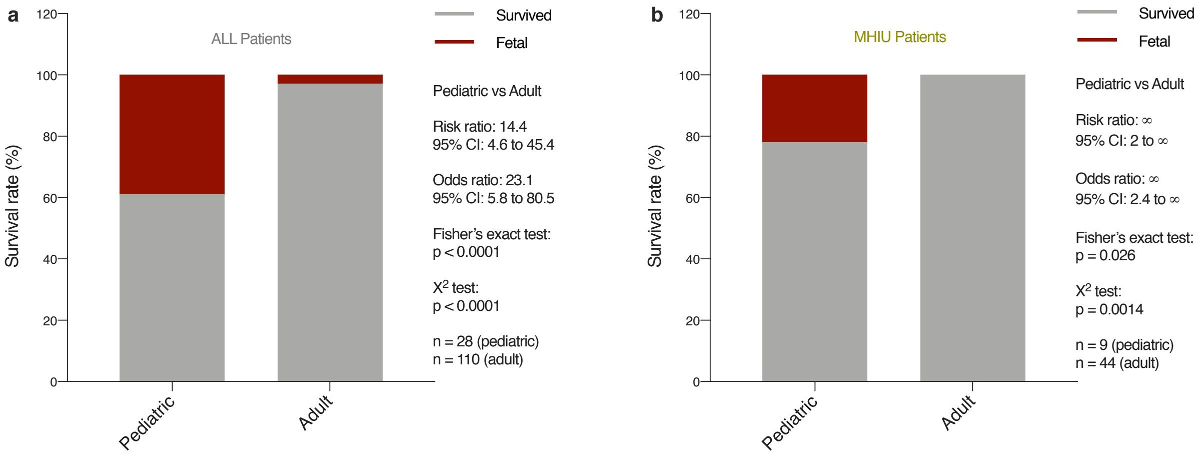

Figure 5. The survival rate of pediatric and adult patients reported among MHS patients in the combined cohort and MHIU subset. (**a**) The survival rate of pediatric and adult patients reported among MHS patient in the combined cohort. (**b**) The survival rate of pediatric and adult patients reported among MHS patient in the MHIU subset. The pediatric subgroup was defined as 17 years of age and younger, and adult subgroup is defined as 18 years of age and older. Risk ratio, odds ratio, statistical test, and the number of patients are indicated.

Based on the combined cohort, we estimated that the risk of death after heat episodes is about 14.4 times higher for pediatric MHS patients compared with adult patients. To avoid potential reporting bias for pediatric patients in the published cohort, we conducted a separate analysis for the MHIU subset. While we found that some pediatric MHS patients did not survive heat episodes, no heat-related mortality was reported among adult patients. Due to the statistical power limited by the patient number in the MHIU subset, we are unable to fairly estimate the risk ratio between pediatric and adult patient as mortality was exclusively reported for pediatric group (risk ratio of infinity). As a conservative estimate, we conclude that the risk ratio of at least 4.6 between the pediatric and adult MHS patients, based on the 95% confidence interval of the estimates from the combined cohort.

RYR1 Genetics

To understand the impact of *RYR1* variants identified in heat-sensitive patients on the structure and function of RYR1, we assessed pathogenicity scores of *RYR1* variants reported in patients. Analysis for the pathogenicity of the heat-sensitivity associated variants in RYR1 using the HumDiv-trained PolyPhen model confirmed that the majority of the variants are predicted deleterious (**Figure 6**).

In particular, highly pathogenic *RYR1* variants associated with heat-sensitivity were frequently found at subunit interfaces near the solenoid structure at the N-terminal domain (Nsol), within the junctional solenoid (JSol) and the bridging solenoid (Bsol) in the cytosolic shell, and at the channel pore domain (Pore) at the C-terminus, based on the previously defined RYR1 domain organization [PMID: 27662087]. Relative to the variants associated with the pharmacologically induced MH episodes, the heat-sensitivity associated variants were mapped to similar domains throughout the RYR1 structure with multiple overlapping residues. The results suggested that dysregulated skeletal

muscle calcium handling due to defects in RYR1 is the fundamental mechanism underlying the pathogenesis of MHS-associated life-threatening response to heat.

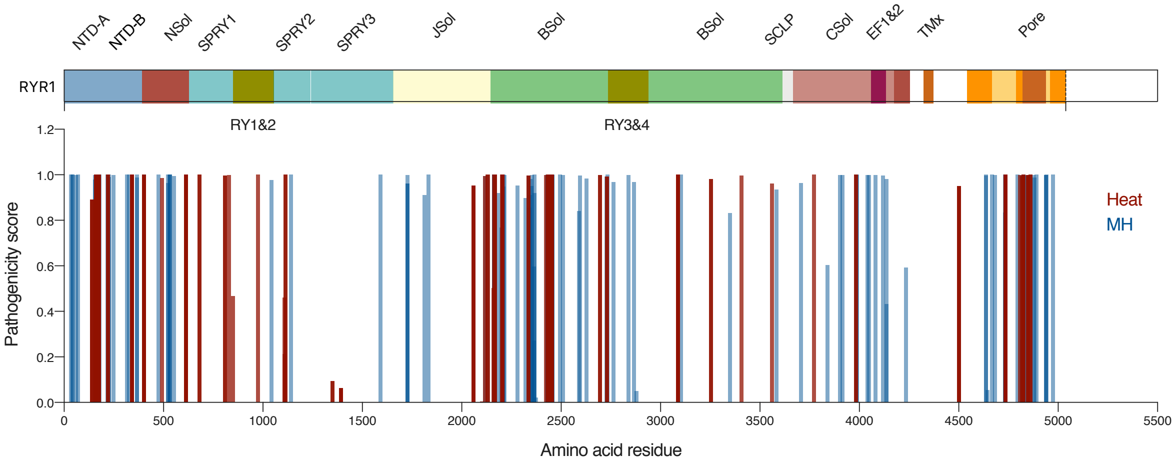

Figure 6. Pathogenicity of *RYR1* variants associated with MH and heat-sensitivity. Residue spans of RYR1 subdomains are defined as previously designated: N-terminal domains A (NTD-A), N-terminal domains B (NTD-B), N-terminal solenoid (NSol), SP1a/ryanodine receptor domain 1 (SPRY1), RYR repeats 1 and 2 (RY1&2), SP1a/ryanodine receptor domain 2 (SPRY2), SP1a/ryanodine receptor domain 3 (SPRY3), junctional solenoid (JSol), bridging solenoid (BSol), RYR repeats 3 and 4 (RY3&4), shell-core linker peptide, CaM and JSol binding sites (SCLP), core solenoid (CSol), EF-hand pair (EF1&2), auxiliary transmembrane helices (TMx), pseudo voltage sensor domain (pVSD), helical-bundle domain between S2 and S3 (S2S3), channel pore domain (Pore), cytoplasmic extension of S6 (S6c), and C-terminal domain (CTD) [PMID: 27662087]. Diagnostic MH mutations are obtained from European Malignant Hyperthermia Group (*www.emhg.org*).

Conclusion

Pathogenic variants in *RYR1* underlie both MH susceptibility to triggering pharmacological agents and the MHS-associated life-threatening hypermetabolic response to heat. In this retrospective cohort study and systematic review, we analyzed the characteristics of MHS patients reported with significant sensitivity to heat. Young age and male gender were identified as significant risk factors associated with significant heat-sensitivity among MHS patients. Further research, including additional longitudinal clinical studies and controlled preclinical experiments, is needed to confirm this finding and to elucidate the underlying mechanism for the pediatric and male predominance. Nevertheless, the identified the risk factors should provide insights for prevention and management of the life-threatening response to heat in MHS patients.

Funding

This study was supported in part by National Institutes of Health (NIH) research project grant (R01) to Susan L. Hamilton from the National Institute of Arthritis and Musculoskeletal and Skin Diseases (NIAMS). Sheila Riazi was supported by the Department of Anesthesia Merit Award. Hui J. Wang was supported in part by the predoctoral fellowship award from the American Heart Association (AHA) and the Howard Hughes Medical Institutes (HHMI) Med into Grad Initiative awarded to the Translational Biology and Molecular Medicine (TBMM) Graduate Program.

Reference

PubMed Identifiers (PMID) are provided for all references.

Supplementary Note 2

STROBE Statement—checklist of items that should be included in reports of observational studies

	Item No	Recommendation	Page no.
**Title and abstract**	1	(*a*) Indicate the study's design with a commonly used term in the title or the abstract	N/A
		(*b*) Provide in the abstract an informative and balanced summary of what was done and what was found	SN1
**Introduction**			
Background/rationale	2	Explain the scientific background and rationale for the investigation being reported	SN2
Objectives	3	State specific objectives, including any prespecified hypotheses	SN2
**Methods**			
Study design	4	Present key elements of study design early in the paper	SN3
Setting	5	Describe the setting, locations, and relevant dates, including periods of recruitment, exposure, follow-up, and data collection	SN3
Participants	6	(*a*) *Cohort study*—Give the eligibility criteria, and the sources and methods of selection of participants. Describe methods of follow-up<br><br>*Case-control study*—Give the eligibility criteria, and the sources and methods of case ascertainment and control selection. Give the rationale for the choice of cases and controls<br><br>*Cross-sectional study*—Give the eligibility criteria, and the sources and methods of selection of participants	SN3<br><br>Cohort study
		(*b*) *Cohort study*—For matched studies, give matching criteria and number of exposed and unexposed<br><br>*Case-control study*—For matched studies, give matching criteria and the number of controls per case	N/A
Variables	7	Clearly define all outcomes, exposures, predictors, potential confounders, and effect modifiers. Give diagnostic criteria, if applicable	SN3
Data sources/ measurement	8*	For each variable of interest, give sources of data and details of methods of assessment (measurement). Describe comparability of assessment methods if there is more than one group	SN3
Bias	9	Describe any efforts to address potential sources of bias	SN4
Study size	10	Explain how the study size was arrived at	N/A
Quantitative variables	11	Explain how quantitative variables were handled in the analyses. If applicable, describe which groupings were chosen and why	SN4
Statistical methods	12	(*a*) Describe all statistical methods, including those used to control for confounding	SN4
		(*b*) Describe any methods used to examine subgroups and interactions	N/A
		(*c*) Explain how missing data were addressed	N/A
		(*d*) *Cohort study*—If applicable, explain how loss to follow-up was addressed<br><br>*Case-control study*—If applicable, explain how matching of cases and controls was addressed<br><br>*Cross-sectional study*—If applicable, describe analytical methods taking account of sampling strategy	N/A
		(*e*) Describe any sensitivity analyses	N/A

Results			
Participants	13*	(*a*) Report numbers of individuals at each stage of study – eg numbers potentially eligible, examined for eligibility, confirmed eligible, included in the study, completing follow-up, and analysed	Table 1
		(*b*) Give reasons for non-participation at each stage	N/A
		(c) Consider use of a flow diagram	N/A
Descriptive data	14*	*(a)* Give characteristics of study participants (eg demographic, clinical, social) and information on exposures and potential confounders	Table 1
		(b) Indicate number of participants with missing data for each variable of interest	N/A
		(c) Cohort study – Summarise follow-up time (eg, average and total amount)	N/A
Outcome data	15*	*(a) Cohort study* – Report numbers of outcome events or summary measures over time	SN5
		(b) Case-control study - Report numbers in each exposure category, or summary measures of exposure	N/A
		(c) Cross-sectional study - Report numbers of outcomes events or summary measures	N/A
Main results	16	*(a)* Give unadjusted estimates and, if applicable, confounder-adjusted estimates and their precision (eg, 95% confidence interval). Make clear which confounders were adjusted for and why they were included	SN5-9
		(b) Report category boundaries when continuous variables were categorized	N/A
		(c) If relevant, consider translating estimates of relative risk and absolute risk for a meaningful time period	N/A
Other analyses	17	Report other analyses done – eg analyses of subgroups and interactions, and sensitivity analyses	N/A
**Discussion**			
Key results	18	Summarise key results with reference to study objectives	SN9
Limitations	19	Discuss limitations of the study, taking into account sources of potential bias or imprecision. Discuss both direction and magnitude of any potential bias	SN9
Interpretation	20	Give a cautious overall interpretation of results considering objectives, limitations, multiplicity of analyses, results from similar studies, and other relevant incidence	SN9
Generalisability	21	Discuss the generalisability (external validity) of the study results	SN9
**Other information**			
Funding	22	Give the source of funding and the role of the funders for the present study and, if applicable, for the original study on which the present article is based	SN9

*Give information separately for cases and controls in case-control studies and, if applicable, for exposed and unexposed groups in cohort and cross-sectional studies.

Note: An Explanation and Elaboration article discusses each checklist item and gives methodological background and published examples of transparent reporting. The STROBE checklist is best used in conjunction with this article (freely available on the Web sites of PLoS Medicine at http://www.plosmedicine.org/, Annals of Internal Medicine at http://www.annals.org/, and Epidemiology at http://www.epidem.com/). Information on the STROBE Initiative is available at www.strobe-statement.org.

Supplementary Note 3

PRISMA-IPD Checklist of items to include when reporting a systematic review and meta-analysis of individual participant data (IPD)

PRISMA-IPD Section/topic	Item No	Checklist item	Reported on page
**Title**			
Title	1	Identify the report as a systematic review and meta-analysis of individual participant data.	SN1
**Abstract**			
Structured summary	2	Provide a structured summary including as applicable:	SN1
		**Background**: state research question and main objectives, with information on participants, interventions, comparators and outcomes.	
		**Methods**: report eligibility criteria; data sources including dates of last bibliographic search or elicitation, noting that IPD were sought; methods of assessing risk of bias.	
		**Results**: provide number and type of studies and participants identified and number (%) obtained; summary effect estimates for main outcomes (benefits and harms) with confidence intervals and measures of statistical heterogeneity. Describe the direction and size of summary effects in terms meaningful to those who would put findings into practice.	
		**Discussion:** state main strengths and limitations of the evidence, general interpretation of the results and any important implications.	
		**Other:** report primary funding source, registration number and registry name for the systematic review and IPD meta-analysis.	
**Introduction**			
Rationale	3	Describe the rationale for the review in the context of what is already known.	SN2
Objectives	4	Provide an explicit statement of the questions being addressed with reference, as applicable, to participants, interventions, comparisons, outcomes and study design (PICOS). Include any hypotheses that relate to particular types of participant-level subgroups.	SN2
**Methods**			
Protocol and registration	5	Indicate if a protocol exists and where it can be accessed. If available, provide registration information including registration number and registry name. Provide publication details, if applicable.	SN3
Eligibility criteria	6	Specify inclusion and exclusion criteria including those relating to participants, interventions, comparisons, outcomes, study design and characteristics (e.g. years when conducted, required minimum follow-up). Note whether these were applied at the study or individual level i.e. whether eligible participants were included (and ineligible participants excluded) from a study that included a wider population than specified by the review inclusion criteria. The rationale for criteria should be stated.	SN3
Identifying studies -	7	Describe all methods of identifying published and unpublished studies including, as applicable: which bibliographic databases were searched with dates of coverage; details of any hand searching including of conference proceedings; use of study registers	SN3

information sources		and agency or company databases; contact with the original research team and experts in the field; open adverts and surveys. Give the date of last search or elicitation.	SN3
Identifying studies - search	8	Present the full electronic search strategy for at least one database, including any limits used, such that it could be repeated.	SN3
Study selection processes	9	State the process for determining which studies were eligible for inclusion.	SN4
Data collection processes	10	Describe how IPD were requested, collected and managed, including any processes for querying and confirming data with investigators. If IPD were not sought from any eligible study, the reason for this should be stated (for each such study).	SN4
		If applicable, describe how any studies for which IPD were not available were dealt with. This should include whether, how and what aggregate data were sought or extracted from study reports and publications (such as extracting data independently in duplicate) and any processes for obtaining and confirming these data with investigators.	
Data items	11	Describe how the information and variables to be collected were chosen. List and define all study level and participant level data that were sought, including baseline and follow-up information. If applicable, describe methods of standardising or translating variables within the IPD datasets to ensure common scales or measurements across studies.	SN4
IPD integrity	A1	Describe what aspects of IPD were subject to data checking (such as sequence generation, data consistency and completeness, baseline imbalance) and how this was done.	SN4
Risk of bias assessment in individual studies.	12	Describe methods used to assess risk of bias in the individual studies and whether this was applied separately for each outcome. If applicable, describe how findings of IPD checking were used to inform the assessment. Report if and how risk of bias assessment was used in any data synthesis.	SN4
Specification of outcomes and effect measures	13	State all treatment comparisons of interests. State all outcomes addressed and define them in detail. State whether they were pre-specified for the review and, if applicable, whether they were primary/main or secondary/additional outcomes. Give the principal measures of effect (such as risk ratio, hazard ratio, difference in means) used for each outcome.	SN4
Synthesis methods	14	Describe the meta-analysis methods used to synthesise IPD. Specify any statistical methods and models used. Issues should include (but are not restricted to):<br>• Use of a one-stage or two-stage approach.<br>• How effect estimates were generated separately within each study and combined across studies (where applicable).<br>• Specification of one-stage models (where applicable) including how clustering of patients within studies was accounted for.<br>• Use of fixed or random effects models and any other model assumptions, such as proportional hazards.<br>• How (summary) survival curves were generated (where applicable).<br>• Methods for quantifying statistical heterogeneity (such as I^2 and τ^2).<br>• How studies providing IPD and not providing IPD were analysed together (where applicable).<br>• How missing data within the IPD were dealt with (where applicable).	SN4

Exploration of variation in effects	A2	If applicable, describe any methods used to explore variation in effects by study or participant level characteristics (such as estimation of interactions between effect and covariates). State all participant-level characteristics that were analysed as potential effect modifiers, and whether these were pre-specified.	NA
Risk of bias across studies	15	Specify any assessment of risk of bias relating to the accumulated body of evidence, including any pertaining to not obtaining IPD for particular studies, outcomes or other variables.	NA
Additional analyses	16	Describe methods of any additional analyses, including sensitivity analyses. State which of these were pre-specified.	NA
**Results**			
Study selection and IPD obtained	17	Give numbers of studies screened, assessed for eligibility, and included in the systematic review with reasons for exclusions at each stage. Indicate the number of studies and participants for which IPD were sought and for which IPD were obtained. For those studies where IPD were not available, give the numbers of studies and participants for which aggregate data were available. Report reasons for non-availability of IPD. Include a flow diagram.	SN5
Study characteristics	18	For each study, present information on key study and participant characteristics (such as description of interventions, numbers of participants, demographic data, unavailability of outcomes, funding source, and if applicable duration of follow-up). Provide (main) citations for each study. Where applicable, also report similar study characteristics for any studies not providing IPD.	SN5
IPD integrity	A3	Report any important issues identified in checking IPD or state that there were none.	NA
Risk of bias within studies	19	Present data on risk of bias assessments. If applicable, describe whether data checking led to the up-weighting or down-weighting of these assessments. Consider how any potential bias impacts on the robustness of meta-analysis conclusions.	SN6
Results of individual studies	20	For each comparison and for each main outcome (benefit or harm), for each individual study report the number of eligible participants for which data were obtained and show simple summary data for each intervention group (including, where applicable, the number of events), effect estimates and confidence intervals. These may be tabulated or included on a forest plot.	SN6-9
Results of syntheses	21	Present summary effects for each meta-analysis undertaken, including confidence intervals and measures of statistical heterogeneity. State whether the analysis was pre-specified, and report the numbers of studies and participants and, where applicable, the number of events on which it is based.	SN6-9
		When exploring variation in effects due to patient or study characteristics, present summary interaction estimates for each characteristic examined, including confidence intervals and measures of statistical heterogeneity. State whether the analysis was pre-specified. State whether any interaction is consistent across trials.	
		Provide a description of the direction and size of effect in terms meaningful to those who would put findings into practice.	
Risk of bias across studies	22	Present results of any assessment of risk of bias relating to the accumulated body of evidence, including any pertaining to the	SN6

		availability and representativeness of available studies, outcomes or other variables.	SN6
Additional analyses	23	Give results of any additional analyses (e.g. sensitivity analyses). If applicable, this should also include any analyses that incorporate aggregate data for studies that do not have IPD. If applicable, summarise the main meta-analysis results following the inclusion or exclusion of studies for which IPD were not available.	NA
**Discussion**			
Summary of evidence	24	Summarise the main findings, including the strength of evidence for each main outcome.	SN9
Strengths and limitations	25	Discuss any important strengths and limitations of the evidence including the benefits of access to IPD and any limitations arising from IPD that were not available.	SN9
Conclusions	26	Provide a general interpretation of the findings in the context of other evidence.	SN9
Implications	A4	Consider relevance to key groups (such as policy makers, service providers and service users). Consider implications for future research.	SN9
**Funding**			
Funding	27	Describe sources of funding and other support (such as supply of IPD), and the role in the systematic review of those providing such support.	SN9

A1 – A3 denote new items that are additional to standard PRISMA items. A4 has been created as a result of re-arranging content of the standard PRISMA statement to suit the way that systematic review IPD meta-analyses are reported.

© Reproduced with permission of the PRISMA IPD Group, which encourages sharing and reuse for non-commercial purposes

Supplementary Note 4

University Health Network
Research Ethics Board
10th Floor, Room 1056
700 University Ave.
Toronto, Ontario, M5G 1Z5
Phone: (416) 581-7849

NOTIFICATION OF REB INITIAL APPROVAL

Date: May 12, 2019

To: Sheila Riazi
Toronto General Hospital, Eaton Building, 200
Elizabeth St., 3rd Floor, Eaton Building, Room 323,
Toronto, Ontario, Canada, M5G 2C4

Re: 19-5365
Analysis of Malignant Hyperthermia (MH) Cases from the Malignant Hyperthermia Investigation Unit (MHIU) database in Toronto

REB Review Type: Delegated
REB Initial Approval Date: May 12, 2019
REB Expiry Date: May 12, 2020

Documents Approved:

Document Name	Version Date	Version ID
Consent Form Addendum	April 30, 2019	
Data Collection Form	April 18, 2019	
Protocol	April 4, 2019	

Documents Acknowledged:

Document Name	Version Date	Version ID
Consent Form CAPCR/UHN REB# 18-5553	July 26, 2018	

The University Health Network Research Ethics Board approves the above mentioned study as it has been found to comply with relevant research ethics guidelines, as well as the Ontario Personal Health Information Protection Act (PHIPA), 2004.

Best wishes on the successful completion of your project.

Sincerely,
Morris Sherman
Co-Chair, University Health Network Research Ethics Board

The UHN Research Ethics Board operates in compliance with the Tri-Council Policy Statement; ICH Guideline for Good Clinical Practice E6(R1); Ontario Personal Health Information Protection Act (2004); Part C Division 5 of the Food and Drug Regulations; Part 4 of the Natural Health Products Regulations and the Medical Devices Regulations of Health Canada. The approval and the views of the REB have been documented in writing.
Furthermore, members of the Research Ethics Board who are named as Investigators in research studies do not participate in discussions related to, nor vote on such studies when they are presented to the REB.
