# Supplementary material for: Adaptive thermogenesis enhances the life-threatening response to heat in mice with an Ryr1 mutation
Source: Nat Commun. 2020 Oct 9;11:5099. doi: 10.1038/s41467-020-18865-z (PMC7547078; doi:10.1038/s41467-020-18865-z)
Supplement: Supplementary file 2 — Description of Additional Supplementary Files [file 41467_2020_18865_MOESM2_ESM.docx]

Description of Additional Supplementary Files

Title: Supplementary Data 1.

Description: Heat-sensitive patients with MHS associated variants in RYR1

from the MHIU cohort. A retrospective cohort study for patients referred to malignant

hyperthermia investigation unit (MHIU) in Toronto during 1994-2019 was performed under the

guidelines from STrengthening the Reporting of OBervational studies in Epidemiology

(STROBE).

Title: Supplementary Data 2.

Description: Heat-sensitive patients with MHS associated variants in RYR1

from published case reports. A systematic review of case reports published during 1980-2019

on heat-sensitivity in patients with MHS was performed under the guidelines from Preferred

Reporting Items for Systematic reviews and Meta-Analysis (PRISMA).

Title: Supplementary Data 3.

Description: Functional proteomic in skeletal muscle of Y524S mice

assessed with RPPA. Reverse phase protein arrays (RPPA) in soleus, EDL, and diaphragm

muscles of the YS mice and wildtype littermate controls (n=3 pairs) under baseline

conditions. Reverse phase protein array (RPPA) of skeletal muscle tissues were performed at

the Functional Proteomics RPPA Core Facility at the University of Texas MD Anderson Cancer

Center. P values are indicated as analyzed by two-sided unpaired t-test without adjustment for

multiple comparisons.

Title: Supplementary Data 4.

Description: Proteomics in filtered serum of Y524S mice assessed with MS.

Mass spectrometry (MS) for peptides from the 10 kDa-filtered serum samples of YS mice and

wildtype littermate controls (n=3 pairs). MS proteomic analysis of filtered serum were performed

at the Mass Spectrometry Proteomics Core at Baylor College of Medicine. P values are

indicated as analyzed by two-sided unpaired t-test without adjustment for multiple comparisons.
