# Supplementary material for: Adaptive thermogenesis enhances the life-threatening response to heat in mice with an Ryr1 mutation
Source: Nat Commun. 2020 Oct 9;11:5099. doi: 10.1038/s41467-020-18865-z (PMC7547078; doi:10.1038/s41467-020-18865-z)
Supplement: Supplementary file 7 — Supplementary Data 5 [file 41467_2020_18865_MOESM7_ESM.zip › Manuscript Source Data/Section 7 Model.pptx]

## Slide 1
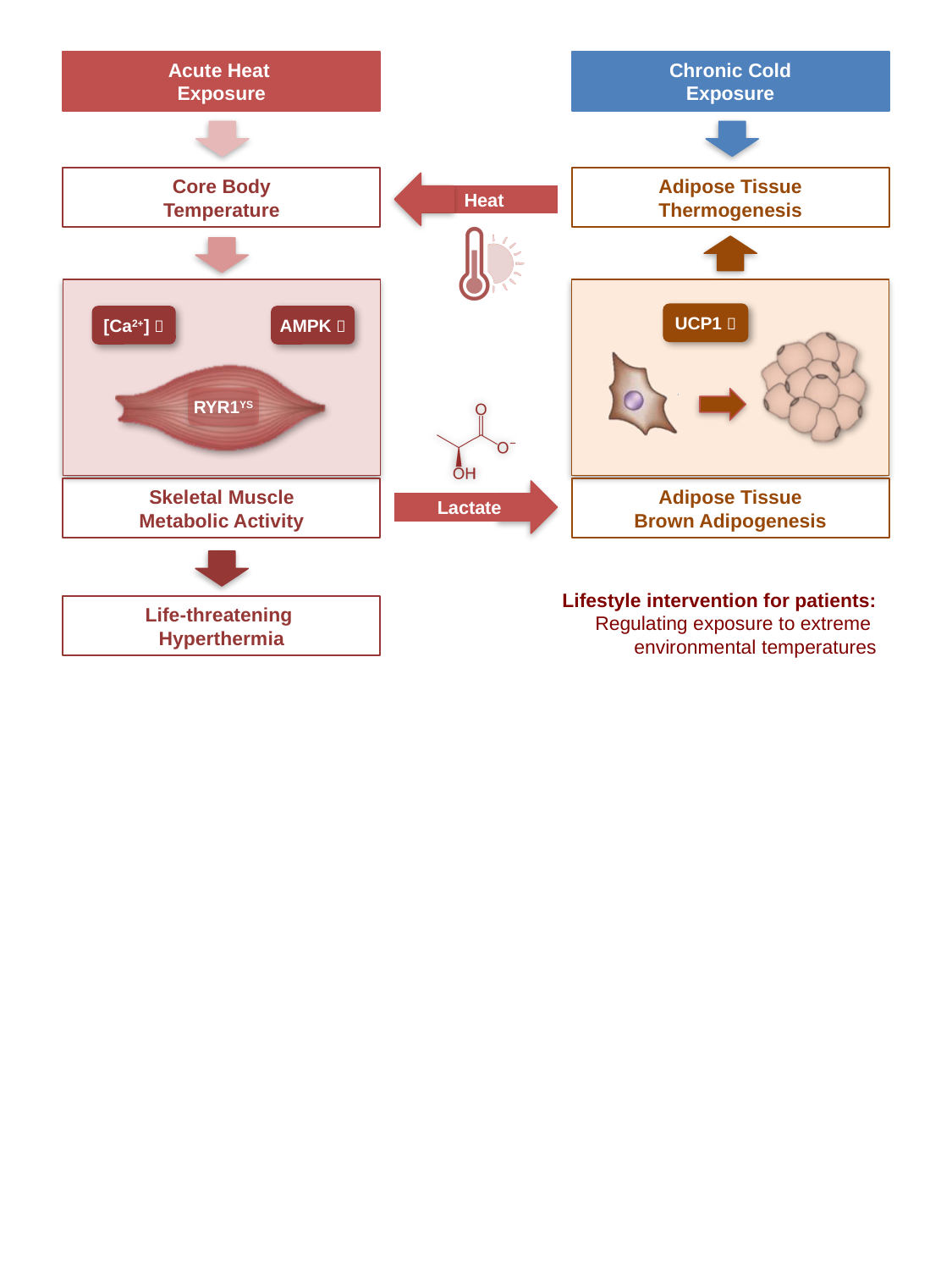

Acute Heat
Exposure
Chronic Cold
Exposure
Core Body
Temperature
Adipose Tissue
Thermogenesis
Heat
UCP1 
[Ca2+] 
AMPK 
RYR1YS
Skeletal Muscle
Metabolic Activity
Adipose Tissue
Brown Adipogenesis
Lactate
Lifestyle intervention for patients:
Regulating exposure to extreme
environmental temperatures
Life-threatening
Hyperthermia

## Slide 2
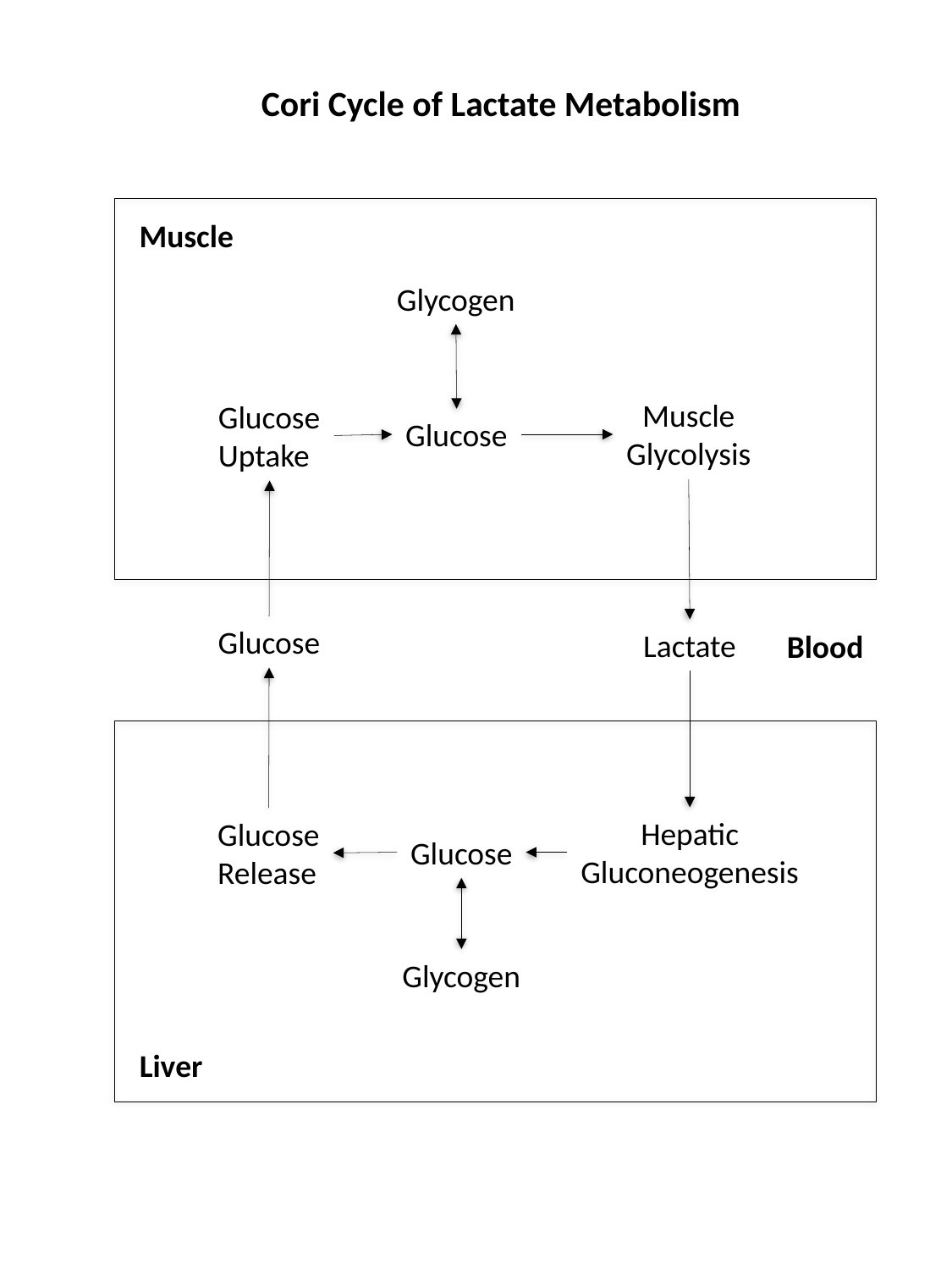

Cori Cycle of Lactate Metabolism
Muscle
Glycogen
Muscle
Glycolysis
Glucose
Uptake
Glucose
Glucose
Lactate
Blood
Hepatic
Gluconeogenesis
Glucose
Release
Glucose
Glycogen
Liver
