# Supplementary material for: Adaptive thermogenesis enhances the life-threatening response to heat in mice with an Ryr1 mutation
Source: Nat Commun. 2020 Oct 9;11:5099. doi: 10.1038/s41467-020-18865-z (PMC7547078; doi:10.1038/s41467-020-18865-z)
Supplement: Supplementary file 7 — Supplementary Data 5 [file 41467_2020_18865_MOESM7_ESM.zip › Manuscript Source Data/Others/RYR1 Structures.pptx]

## Slide 1
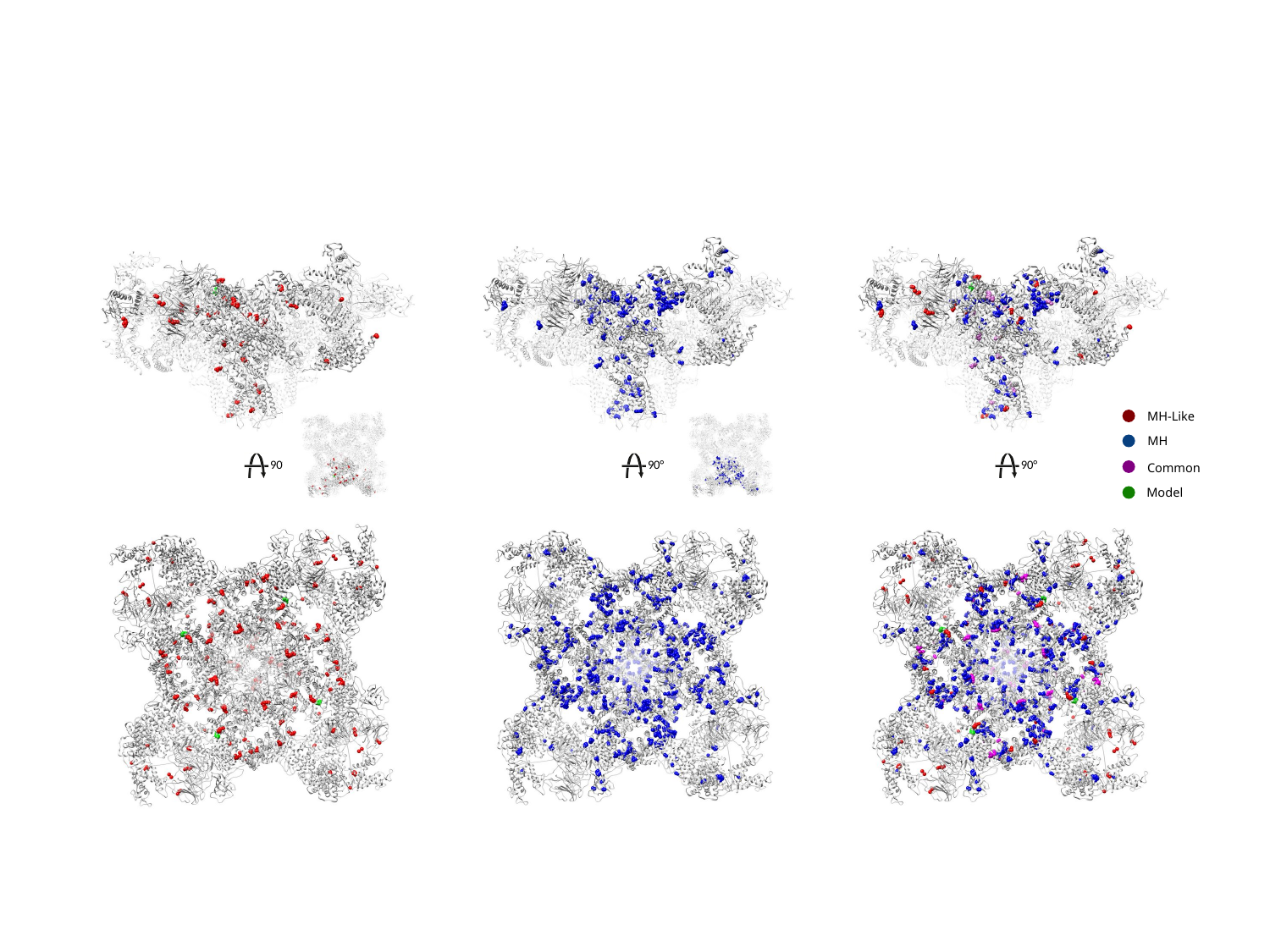

MH-Like
MH
90°
90°
90°
Common
Model

## Slide 2
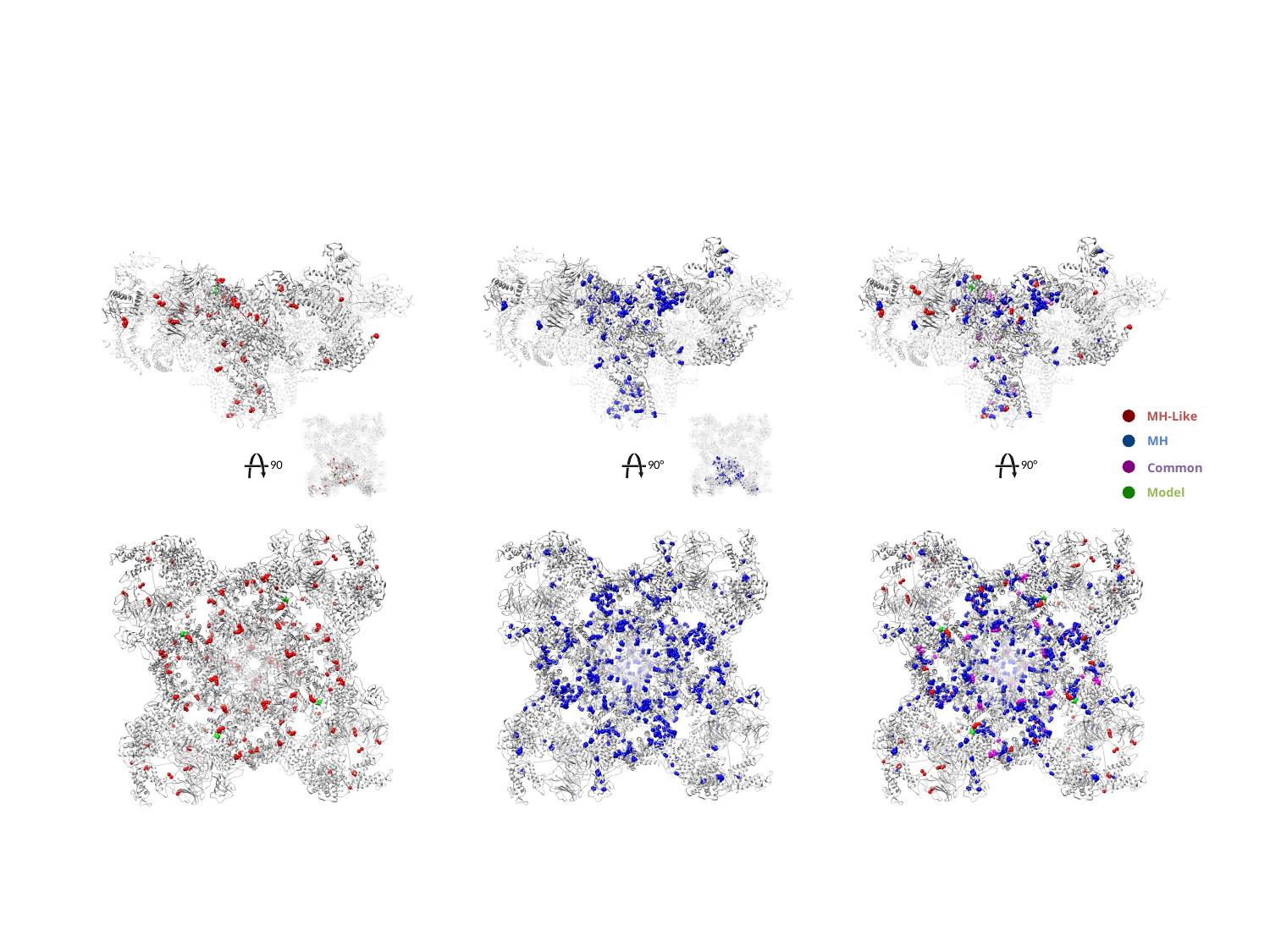

MH-Like
MH
90°
90°
90°
Common
Model

## Slide 3
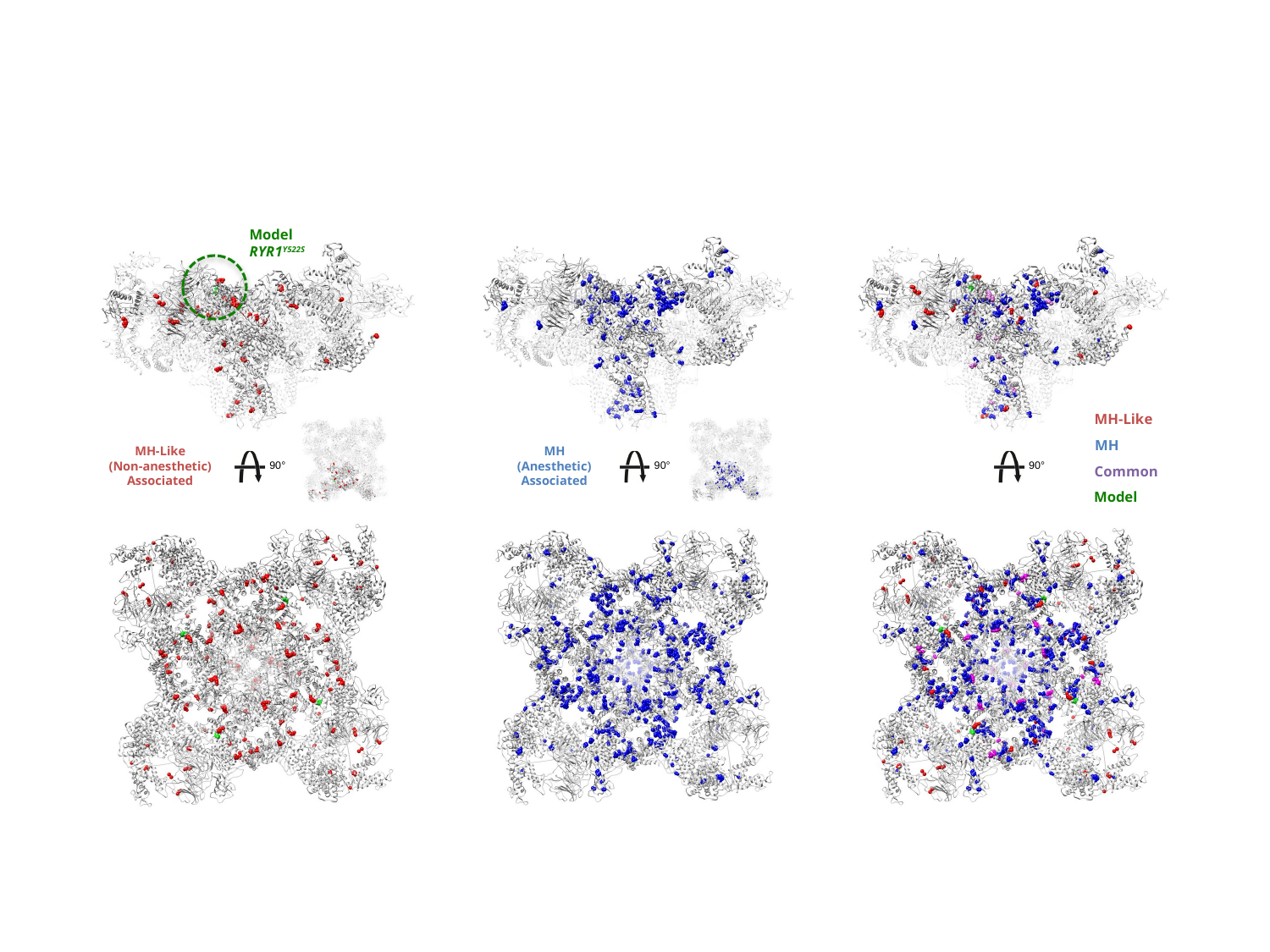

MH-Like
MH
Common
Model
90°
90°
90°
Model
RYR1Y522S
MH-Like
(Non-anesthetic)
Associated
MH
(Anesthetic)
Associated

## Slide 4
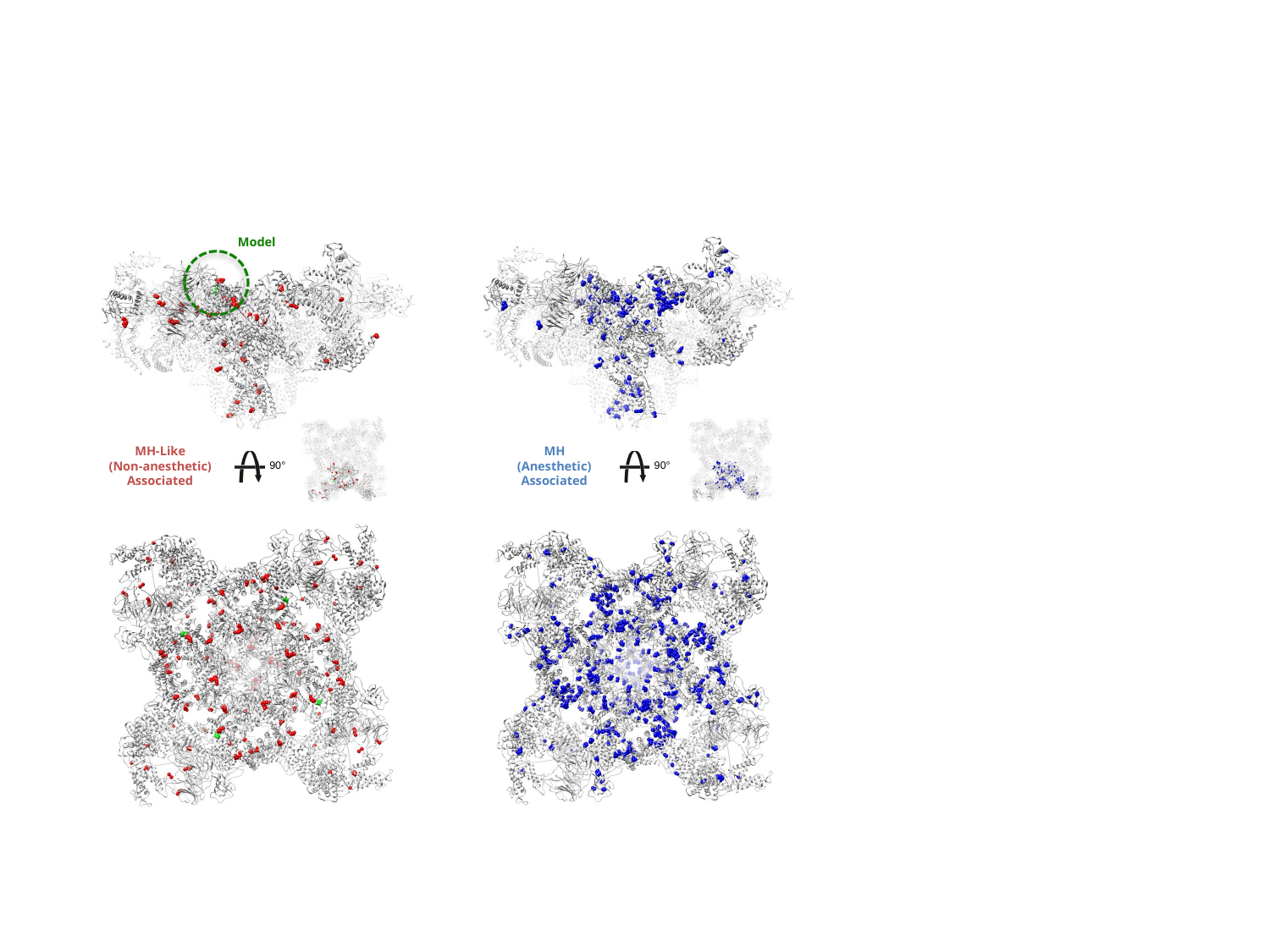

Model
MH-Like
(Non-anesthetic)
Associated
MH
(Anesthetic)
Associated
90°
90°

## Slide 5
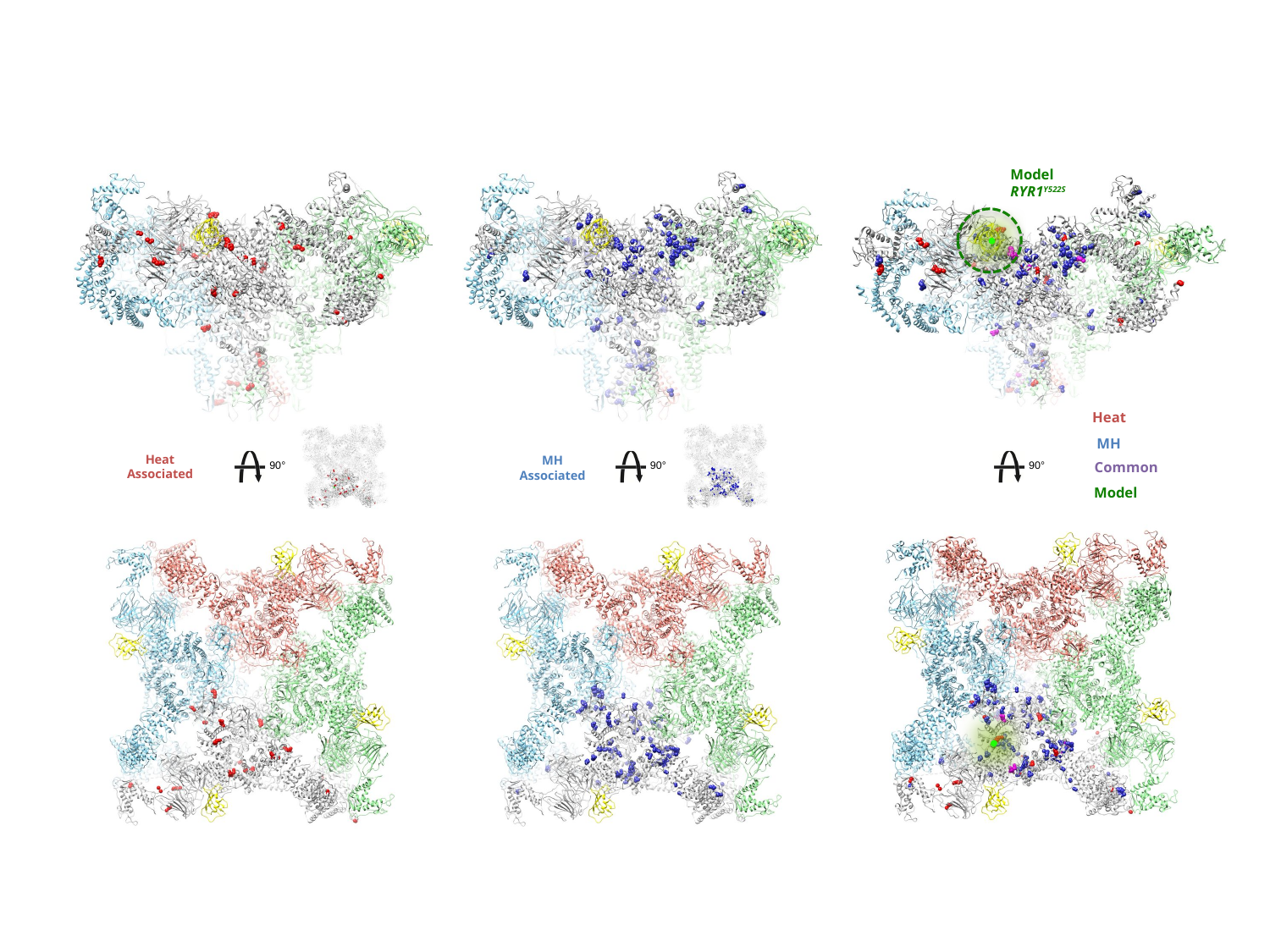

Model
RYR1Y522S
Heat
MH
Heat
Associated
MH
Associated
90°
90°
90°
Common
Model

## Slide 6
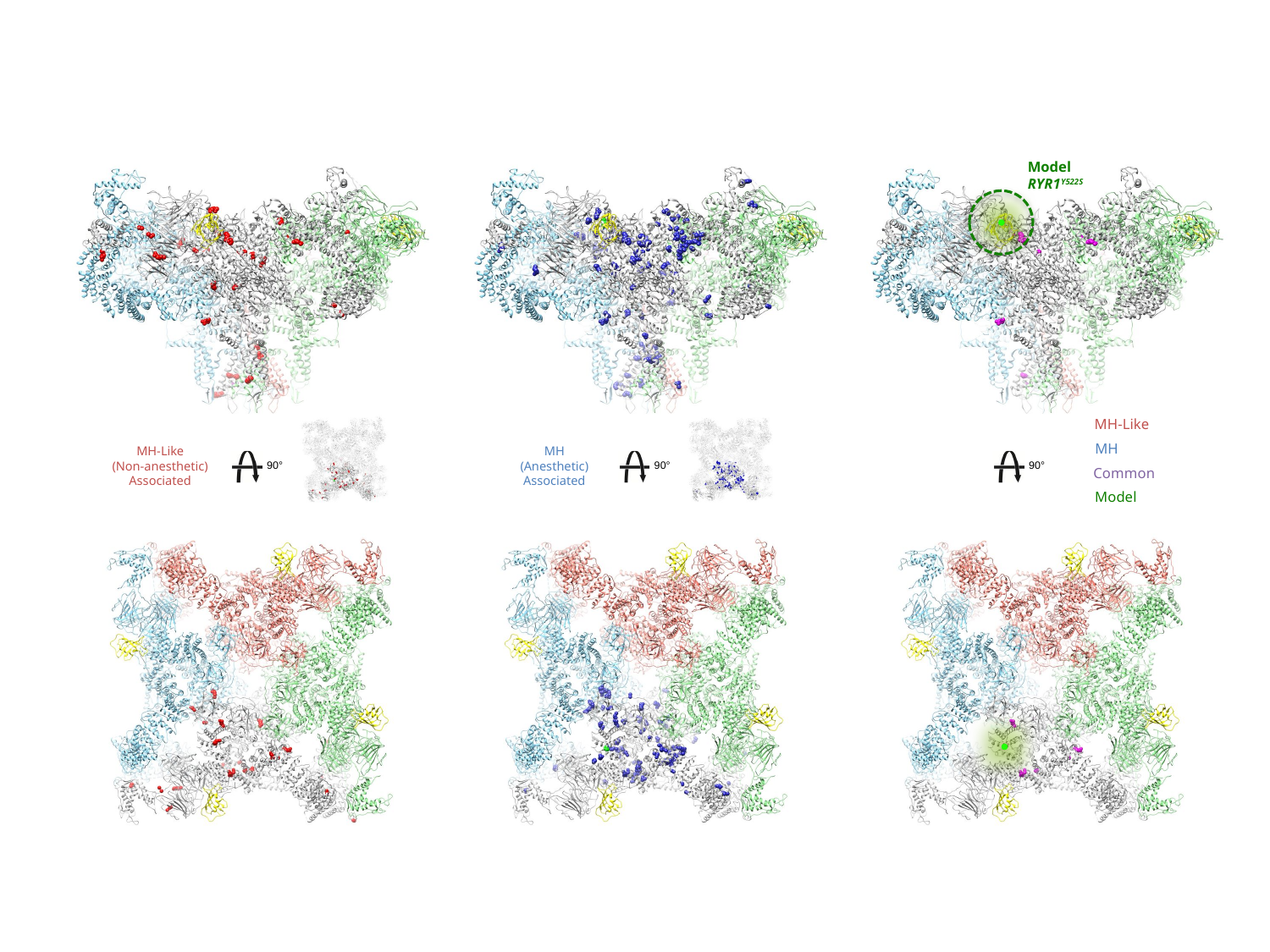

Model
RYR1Y522S
MH-Like
MH
MH-Like
(Non-anesthetic)
Associated
MH
(Anesthetic)
Associated
90°
90°
90°
Common
Model

## Slide 7
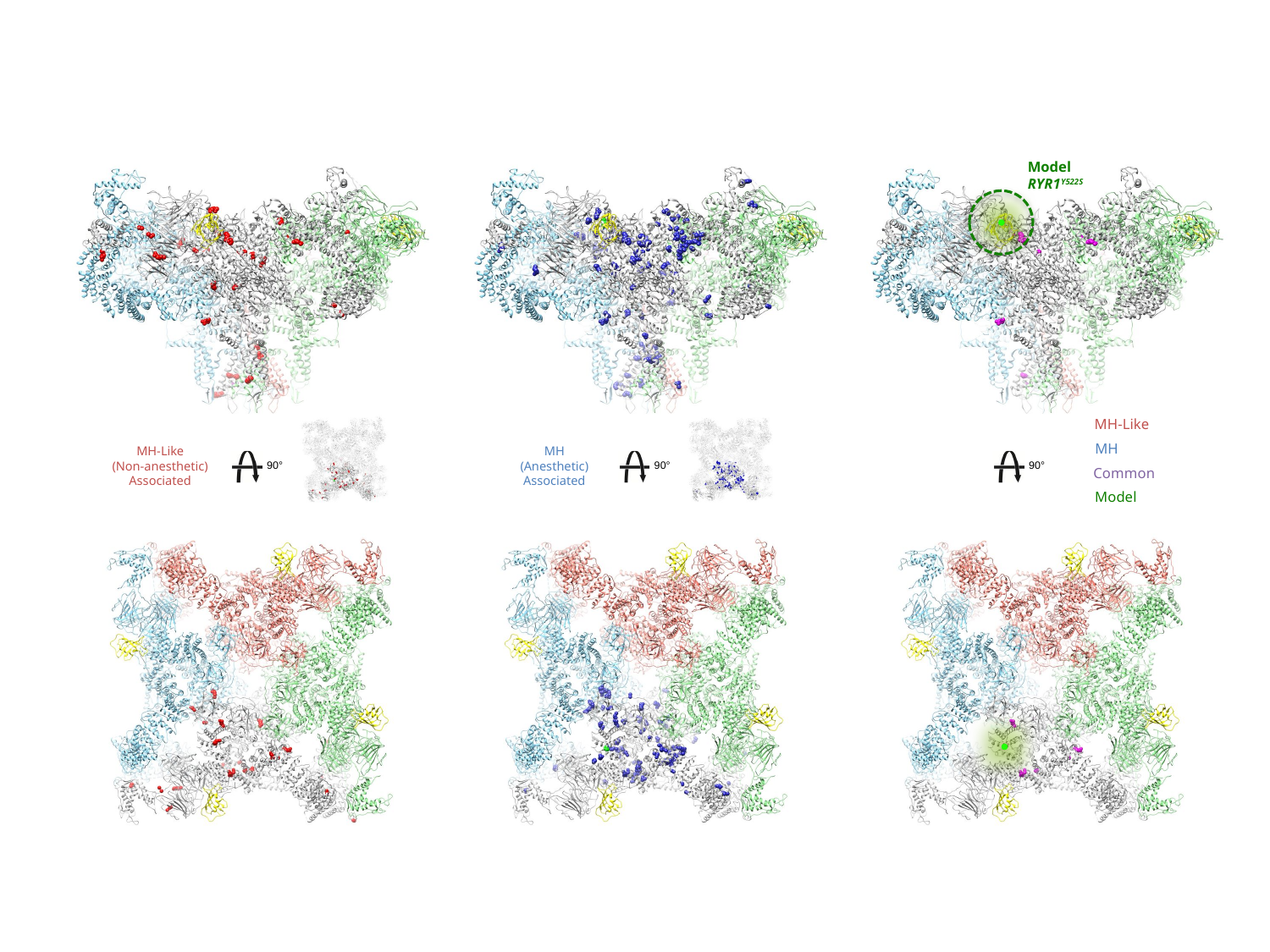

Model
RYR1Y522S
MH-Like
MH
MH-Like
(Non-anesthetic)
Associated
MH
(Anesthetic)
Associated
90°
90°
90°
Common
Model

## Slide 8
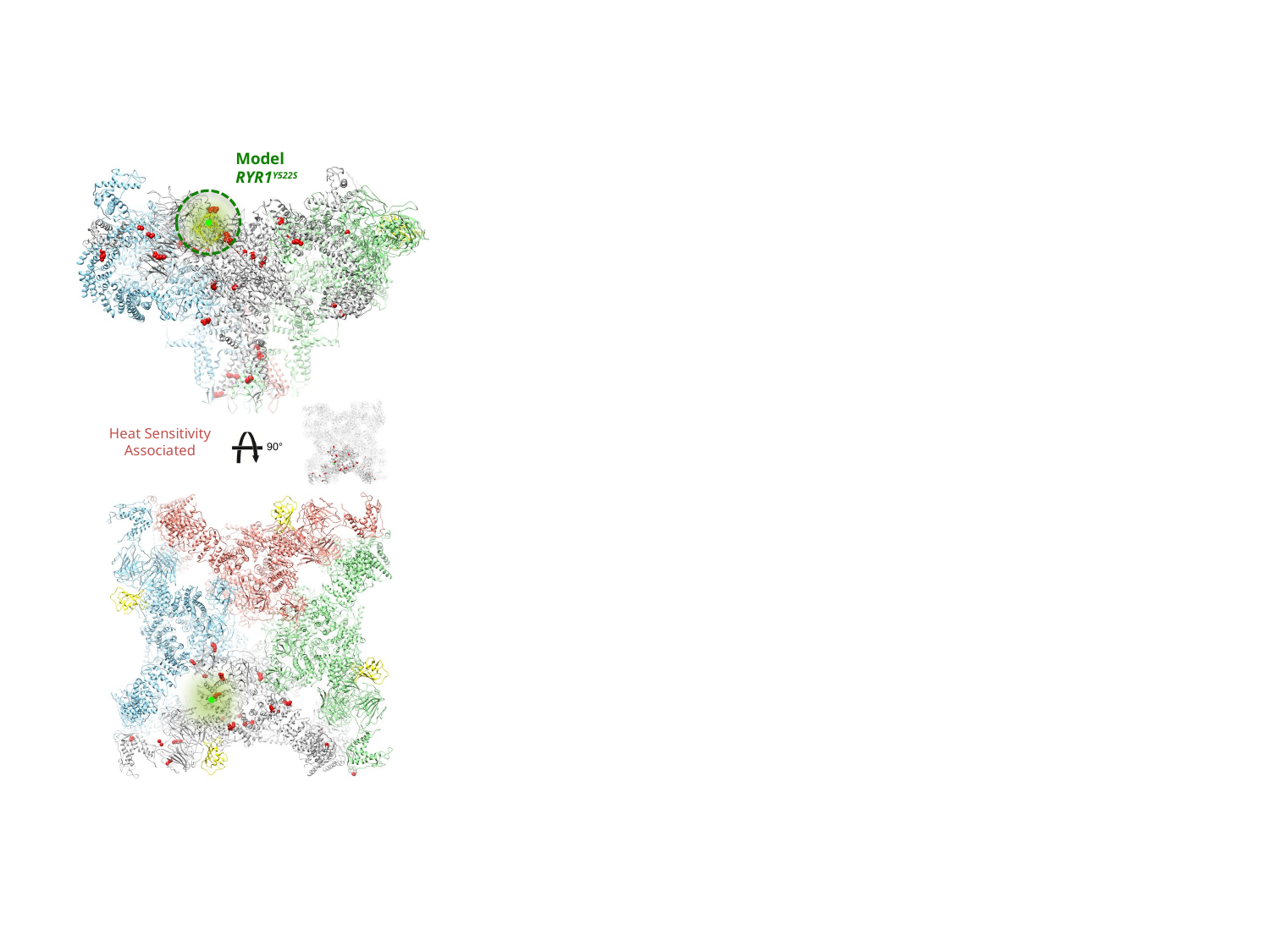

Model
RYR1Y522S
Heat Sensitivity
Associated
90°
