# Supplementary material for: Adaptive thermogenesis enhances the life-threatening response to heat in mice with an Ryr1 mutation
Source: Nat Commun. 2020 Oct 9;11:5099. doi: 10.1038/s41467-020-18865-z (PMC7547078; doi:10.1038/s41467-020-18865-z)
Supplement: Supplementary file 8 — Reporting Summary [file 41467_2020_18865_MOESM8_ESM.pdf]

nature research

Corresponding author(s): Susan L. Hamilton

Last updated by author(s): Aug 10, 2020

Reporting Summary

Nature Research wishes to improve the reproducibility of the work that we publish. This form provides structure for consistency and transparency in reporting. For further information on Nature Research policies, see Authors & Referees and the Editorial Policy Checklist .

Statistics

For all statistical analyses, confirm that the following items are present in the figure legend, table legend, main text, or Methods section.

n/a	Confirmed	
☐	☒	The exact sample size (*n*) for each experimental group/condition, given as a discrete number and unit of measurement
☐	☒	A statement on whether measurements were taken from distinct samples or whether the same sample was measured repeatedly
☐	☒	The statistical test(s) used AND whether they are one- or two-sided<br>*Only common tests should be described solely by name; describe more complex techniques in the Methods section.*
☐	☒	A description of all covariates tested
☐	☒	A description of any assumptions or corrections, such as tests of normality and adjustment for multiple comparisons
☐	☒	A full description of the statistical parameters including central tendency (e.g. means) or other basic estimates (e.g. regression coefficient) AND variation (e.g. standard deviation) or associated estimates of uncertainty (e.g. confidence intervals)
☐	☒	For null hypothesis testing, the test statistic (e.g. *F*, *t*, *r*) with confidence intervals, effect sizes, degrees of freedom and *P* value noted<br>*Give P values as exact values whenever suitable.*
☒	☐	For Bayesian analysis, information on the choice of priors and Markov chain Monte Carlo settings
☒	☐	For hierarchical and complex designs, identification of the appropriate level for tests and full reporting of outcomes
☐	☒	Estimates of effect sizes (e.g. Cohen's *d*, Pearson's *r*), indicating how they were calculated

Our web collection on statistics for biologists contains articles on many of the points above.

Software and code

Policy information about availability of computer code

Data collection	CLAMS/IDC (Oxymax, Columbus Instruments), PET/CT (Inveon Acquisition Workplace, Siemens), NMR spectroscopy (ISIS, Bruker), Immunoblot (ChemiDoc, BioRad), RT-PCR (ViiA7, Applied Biosystems), Optical density (Gen5, BioTek), MILIPLEX (Luminex, EMD Millipore), Bright-field microscopy (NIS, Nikon), Fluorescent microscopy (IX81, Olympus), Polychrome V monochromator-based illumination system (TILL Photonics), Flow cytometry (FACSDiva, BD), and Mass Spectrometry (Nano-HPLC 1200, Thermo Scientific)
Data analysis	PET/CT (Inveon Research Workplace 4.0, Siemens), NMR spectroscopy (Paravision 5.1, Bruker), Immunoblot (ImageJ 1.5, NIH), Variant pathogenicity (PolyPhen 2.0, Harvard), Protein Structure (Chimera 1.1, UCSF), Flow cytometry (FlowJo 10.7, BD), Mass Spectrometry (Xcalibur 4.1.50, Thermo Fisher Scientific), RefSeq Search (Proteome Discoverer 2.1 interface, Thermo Fisher & Mascot 2.4, Matrix Science), Statistics for null hypothesis testing (Prism 8.0, GraphPad) and power calculation (RStudio 1.0.143, R)

For manuscripts utilizing custom algorithms or software that are central to the research but not yet described in published literature, software must be made available to editors/reviewers. We strongly encourage code deposition in a community repository (e.g. GitHub). See the Nature Research guidelines for submitting code & software for further information.

Data

Policy information about availability of data

All manuscripts must include a data availability statement. This statement should provide the following information, where applicable:

- Accession codes, unique identifiers, or web links for publicly available datasets
- A list of figures that have associated raw data
- A description of any restrictions on data availability

All data generated or analyzed during this study are included in this published article and its supplementary information files. The mass spectrometry data have been deposited via the Mass Spectrometry Interactive Virtual Environment (MassIVE) repository. Source data are provided with this paper and open for the public: MSV000085803 [ftp://massive.ucsd.edu/MSV000085803]. Diagnostic MH mutations are obtained from European Malignant Hyperthermia Group (www.emhg.org) and are publicly available. Structures of RYR1 are generated based on data from protein data bank (PDB ID: 5T15) and are publicly available.

Field-specific reporting

Please select the one below that is the best fit for your research. If you are not sure, read the appropriate sections before making your selection.

- [x] Life sciences
- [ ] Behavioural & social sciences
- [ ] Ecological, evolutionary & environmental sciences

For a reference copy of the document with all sections, see nature.com/documents/nr-reporting-summary-flat.pdf

Life sciences study design

All studies must disclose on these points even when the disclosure is negative.

Sample size

Sample sizes were determined based on power analysis of preliminary results or estimated based on similar previous studies when preliminary results were not available.

Data exclusions

No samples were excluded from the statistical analysis unless specifically stated otherwise. The following data points were excluded based on pre-established criteria:

One data point (YS vehicle MaxVO2 = 161 ml/kg/min) identified as a statistical outlier (more than 2.57 σ above the group mean) was excluded from the analysis based on Grubb's method (Supplementary Figure 6 f).

One data point (WT [Pi] = 21.94 a.u.) identified as a statistical outlier (more than 2.41 σ above the group mean) was excluded from the analysis based on Grubb's method (Supplementary Figure 9 h).

One data point (Lactate + ARC = 182.6% of control) identified as a statistical outlier (more than 2.04 σ above the group mean) was excluded from the analysis based on Grubb's method (Figure 6 d).

Replication

The number of biological replicates (sample sizes) for each experiment was indicated. All key data were retrieved from at least 3 independent experiments, except for retrospective studies.

Further, the results from key studies were independently confirmed by at least 2 contributing investigators:

Age-dependent heat sensitivity (HJW, DKG), Gender-dependent heat sensitivity (HJW, RSY), Ambient temperature-dependent heat sensitivity (HJW, RSY), Elevated baseline core body temperature (HJW, RSY), Genetic brown fat modulation on heat sensitivity (HJW, CSL), Pharmacological brown fat modulations on heat sensitivity (HJW, CSL), Enhanced brown fat thermogenic capacities (HJW, CSL), Enhanced white fat thermogenic capacities (HJW, CSL), Elevated muscle glycolysis (HJW, JH), Elevated circulating lactate levels (HJW, JH), and Brown adipogenic effect of lactate (HJW, CSL).

Randomization

For experiment involving animal subjects, mice were allocated into experimental groups with a specifically designed randomization mechanism.

Briefly, unique animal identifiers (mating cage number/litter number/earmark/ID) were assigned to each mouse at weaning based on a fully randomized order of selection from a mating cage. After genotypes are identified, mice of each genotype were allocated into treatment groups by the order of the unique animal identifiers alternately prior to any data collection, such that mice of each genotype were close to evenly distributed among all treatment groups without bias.

Littermate pairs were required for all experiments in our study to control for covariates influenced by any genetic and environmental factors.

For experiment involving cultured cells, samples were alternately allocated into experimental groups, such that technical variabilities in sample processing are randomized among experimental groups.

Blinding

We confirm that the investigators were blinded for the following studies, where statistical outlier(s) had been excluded:

Muscle inorganic phosphate levels (Supplementary Figure 9 e) were assessed by the investigators (JR, RGP) who were blinded during data collection. Blinding was done by an independent investigator (HJW) who randomized and masked the animal information prior to assessment. The statistical outlier excluded was confirmed by all investigators who collected (JR, RGP), analyzed (RGP, HJW) and approved (SLH) the data.

Blinding was not possible for the drug treatment experiments (Figure 7 d and Supplementary Figure 6 f) as genotype and treatment were revealed for group assignment (randomized but not blinded) prior to assessment. The statistical outlier excluded was confirmed by all investigators who collected (HJW, CSL), analyzed (HJW, CSL) and approved (SLH) the data.

Investigators were also blinded for other studies during data collection whenever possible. However, blinding was not relevant as no statistical outliers were excluded from these studies.

Reporting for specific materials, systems and methods

We require information from authors about some types of materials, experimental systems and methods used in many studies. Here, indicate whether each material, system or method listed is relevant to your study. If you are not sure if a list item applies to your research, read the appropriate section before selecting a response.

Materials & experimental systems

n/a	Involved in the study
☐	☒ Antibodies
☐	☒ Eukaryotic cell lines
☒	☐ Palaeontology
☐	☒ Animals and other organisms
☐	☒ Human research participants
☒	☐ Clinical data

Methods

n/a	Involved in the study
☒	☐ ChIP-seq
☐	☒ Flow cytometry
☒	☐ MRI-based neuroimaging

Antibodies

Antibodies used

The information on the list of antibodies used can be found in Supplementary Table 1.

Validation

The following primary antibody validation information can be found in the manufacturer's website:

1. Anti-UCP1-m antibody (AB10983) from Abcam: Rabbit polyclonal anti-UCP1 antibody for mouse. Validated for immunoblotting and immunofluorescence applications. Cited in 255 publications.

2. Anti-EBF2 antibody (BS-11740R-A647) from Bioss: Rabbit polyclonal anti-EBF2-AlexaFluor647 conjugated antibody for human, and mouse. Validated for immunofluorescence applications. Validated with controls.

3. Anti-VDAC antibody (4866S) from Cell Signaling: Rabbit polyclonal anti-VDAC antibody for human and mouse. Validated for immunoblotting applications. Cited in 130 publications.

4. Anti-SIRT3 antibody (5490S) from Cell Signaling: Rabbit monoclonal anti-SIRT3 antibody for human and mouse. Validated for immunoblotting applications. Cited in 69 publications.

5. Anti-MFN2 antibody (9482S) from Cell Signaling: Rabbit monoclonal anti-MFN2 antibody for human and mouse. Validated for immunoblotting applications. Cited in 53 publications.

6. Anti-CYC antibody (4280) from Cell Signaling: Rabbit monoclonal anti-CYC antibody for human and mouse. Validated for immunoblotting applications. Cited in 83 publications.

7. Anti-Tubulin antibody (6G7) from DSHB: Mouse monoclonal anti-tubulin-β-III antibody for human and mouse. Validated for immunoblotting applications. Cited in 8 publications.

8. Anti-CD140a (12-1401-81) from eBioscience: Rat monoclonal anti-CD140a antibody for human and mouse. Validated for immunofluorescence applications. Cited in 38 publications.

9. Anti-PGC1-alpha antibody (NBP1-04676) from NOVUS Biologicals: Rabbit polyclonal anti-PGC1-alpha antibody for human and mouse. Validated for immunoblotting applications. Cited in 93 publications.

10. Anti-CD36 (NB110-59724) from NOVUS Biologicals: Mouse monoclonal anti-CD36 antibody for mouse. Validated for immunoblotting and immunofluorescence. Cited in 9 publications.

11. Anti-MnSOD (SC-133254) from Santa Cruz: Mouse monoclonal anti-MnSOD2 antibody for human and mouse. Validated for immunoblotting applications. Cited in 12 publications.

12. Anti-FATP1 (SC-25541) from Santa Cruz: Rabbit polyclonal anti-FATP1 antibody for human and mouse. Validated for immunoblotting applications. Cited in 14 publications.

13. Anti-UCP1-h antibody (PA1-24894) from Invitrogen: Rabbit polyclonal anti-UCP1 antibody for human and mouse. Validated for immunoblotting applications. Cited in 11 publications.

14. Anti-CaMKK-beta antibody (GTX108305) from GeneTex: Rabbit polyclonal anti-CaMKK-beta antibody for human and mouse. Validated for immunoblotting applications. Validated with controls.

15. Anti-phospho-CaMKK-beta (12818S) from Cell Signaling: Rabbit polyclonal anti-phospho-CaMKK-beta antibody for human and mouse. Validated for immunoblotting applications. Cited in 2 publications.

Additional validation data for antibodies with positive and negative controls were also provided in Supplementary Information.

Eukaryotic cell lines

Policy information about cell lines

Cell line source(s)	The mouse 3T3-L1 cell line used in this study was directly purchased (ATCC® CL-173™) from the American Type Culture Collection (ATCC).<br><br>The human hTERT-A41hBAT-SVF cell line used in this study was directly purchased (ATCC® CRL-3385™) from the American Type Culture Collection (ATCC).
Authentication	The mouse 3T3-L1 cells used in this study have been “thoroughly tested and authenticated” prior to shipping on February 28, 2019 (ATCC Delivery ID: 1515A), according to the certificate of analysis (ATCC LOT No.: 70013338) from American Type Culture Collection (ATCC). Techniques for authentication involve COI assay and adipocyte formation.<br><br>The human hTERT-A41hBAT-SVF cells used in this study have been “thoroughly tested and authenticated” prior to shipping on April 14, 2020 (ATCC Delivery ID: 1515A), according to the certificate of analysis (ATCC LOT No.: 70008125) from American Type Culture Collection (ATCC). Techniques for authentication involve COI assay, STR analysis and Karyotyping.
Mycoplasma contamination	The mouse 3T3-L1 cells used in this study have been “thoroughly tested and authenticated” prior to shipping on February 28, 2019 (ATCC Delivery ID: 1515A), according to the certificate of analysis (ATCC LOT No.: 70013338) from American Type Culture Collection (ATCC). Mycoplasma contamination was NOT detected based on Hoechst DNA stain (indirect) method, Agar culture (direct) method, and PCR-based assay.<br><br>The human hTERT-A41hBAT-SVF cells used in this study have been “thoroughly tested and authenticated” prior to shipping on April 14, 2020 (ATCC Delivery ID: 1515A), according to the certificate of analysis (ATCC LOT No.: 70008125) from American Type Culture Collection (ATCC). Mycoplasma contamination was NOT detected based on Hoechst DNA stain (indirect) method, Agar culture (direct) method, and PCR-based assay.
Commonly misidentified lines<br>(See ICLAC register)	The mouse 3T3-L1 cell line used in this study was NOT found to be one of the Misidentified Cell Lines curated by the international cell line authentication committee (ICLAC), based on the result of a recent search from the most up-to-date NCBI BioSample database (Version 9).<br><br>The human hTERT-A41hBAT-SVF cell line used in this study was NOT found to be one of the Misidentified Cell Lines curated by the international cell line authentication committee (ICLAC), based on the result of a recent search from the most up-to-date NCBI BioSample database (Version 9).

Animals and other organisms

Policy information about studies involving animals; ARRIVE guidelines recommended for reporting animal research

Laboratory animals	Mouse (mus musculus) maintained on C57BL6/J genetic background were used in this study. Detailed gender and age information of mice were specified for each experiment in the manuscript.
Wild animals	This study did NOT involve wild animals.
Field-collected samples	This study did NOT involve samples collected from the field.
Ethics oversight	The institutional animal care and use committee (IACUC) at Baylor College of Medicine approved all protocols for animal experiments in this study.

Note that full information on the approval of the study protocol must also be provided in the manuscript.

Human research participants

Policy information about studies involving human research participants

Population characteristics	Detailed population characteristics of human research participants on age, gender, genotypic information, past and current diagnosis and treatment, were included in the Supplementary Information.<br><br>Among the malignant hyperthermia susceptible (MHS) patients referred to MH investigation unit (MHIU) between 1994 and 2019, at least 443 of 783 MHS patients (56.6%) complained of having one or more symptoms of heat-sensitivity. In contrast, only 26 out of 1008 MH negative (MHN) patients (2.5%) reported any heat-sensitivity symptoms, and none displayed significantly heat-sensitivity symptoms. In this retrospective cohort study, we focused on 87 MHS patients from 33 families, consisting of 48 patients who had one or more significant heat-sensitivity symptoms as well as their 34 asymptomatic and five mildly

symptomatic family members who are carriers of the same RYR1 variants. Symptoms of heat-sensitivity include profuse sweating, heat cramps, intolerance to hot temperature, heat exhaustion, heat-induced rhabdomyolysis, and heatstroke. Significant heat-sensitivity symptoms were defined as suffering from heat exhaustion, heat-induced rhabdomyolysis, or occurrence of heatstroke. All patients included in the MHIU cohort are provided with complete age, gender, symptom, and survival outcome information.

The clinical and genetic information for all patients identified in the MHIU cohort is presented in Supplementary Table 1.

Recruitment

Detailed inclusion criteria of human research participants were included in the Supplementary Information.

Patients referred to the malignant hyperthermia investigation unit (MHIU) at Toronto General Hospital between 1994 and 2019 were included in the retrospective cohort study performed under the STrengthening the Reporting of OBervational studies in Epidemiology (STROBE) guidelines.

Eligibility Criteria. MHS Patients who had significant heat-sensitivity symptoms and carried a variant in RYR1 were included. The significant heat sensitivity was defined as per patient's report as suffering from heat exhaustion, the occurrence of heatstroke or heat-induced rhabdomyolysis. Family members of patients who carried the same RYR1 variants were also included.

Variables. Collected data included RYR1 variants, gender, age at the time of assessment of heat sensitivity, description of symptoms, information on MH reaction if existed, such as clinical grading scale score (CGS), caffeine and halothane contracture results if available, blood lactate levels where available, and previous anesthetic history. The same information from family members, who carried the same RYR1 variants, was collected.

Ethics oversight

Notification of REB approval is provided in the Supplementary Information.

The institutional research ethics board (REB) at the University of Toronto approved this retrospective clinical study and waived the need for informed consent.

Note that full information on the approval of the study protocol must also be provided in the manuscript.

Flow Cytometry

Plots

Confirm that:

- [x] The axis labels state the marker and fluorochrome used (e.g. CD4-FITC).
- [x] The axis scales are clearly visible. Include numbers along axes only for bottom left plot of group (a 'group' is an analysis of identical markers).
- [x] All plots are contour plots with outliers or pseudocolor plots.
- [x] A numerical value for number of cells or percentage (with statistics) is provided.

Methodology

Sample preparation

Detailed procedures for sample preparation are provided in the method section.

For primary stromal vascular fraction (SVF) cells, adipose tissue from interscapular region was excised from neonates at P2.5, minced in phosphate buffered saline (PBS), digested with collagenase, and filtered through 70 µm cell strainer. For cultured SVF cells, adherent cells in dish were harvested by gentle scrapping without the use of trypsin. Collected cells were immediately resuspended in standard flow cytometry (FC) staining buffer containing 0.1% sodium azide. Directly conjugated antibodies (anti-PDGFRα/CD140a-PE, 1:400) was added to cell pallet to stain surface antigens in dark at 4°C for 45 minutes. After washing the cell pallets with FC staining buffer, 100µl of 4% paraformaldehyde was added to fix the cells for 15 minutes, and cells were subsequently permeabilized with 100µl 0.2% Triton X-100. After additional washing with FC staining buffer, directly conjugated antibodies (anti-EBF2-AlexaFluor647, 1:50) and DAPI (1µg/mL) was added to cell pallet to stain nuclear targets in dark at 4°C for 45 minutes. Stained cells were analyzed on flow cytometer within 24 hours.

Instrument

BD LSRFortessa Cell Analyzer was used to acquire flow cytometry data.

Software

BD FACSDiva was used for experiment setup and acquisition of flow cytometry data from BD LSRFortessa workstation. BD FlowJo was used for flow cytometry data analysis. Graphpad Prism8 was used for further statistical analysis on relative cell frequencies and fluorescence intensities.

Cell population abundance

Flow cytometry was used for cell quantification only. Post-sort fraction from cell sorting was not collected.

Gating strategy

Detailed gating strategies for all relevant experiments are provided in the Supplementary Figure 15.

Briefly, forward scatter (FCS) and side scatter (SSC) gates of starting cell population, gates for singlets screening, and threshold for positive and negative populations are indicated. Identical gating threshold was applied to all samples within the experiment.

- [x] Tick this box to confirm that a figure exemplifying the gating strategy is provided in the Supplementary Information.
